# Supplementary material for: Precise cooperative sulfur placement leads to semi-crystallinity and selective depolymerisability in CS2/oxetane copolymers
Source: Nat Commun. 2023 Jul 27;14:4525. doi: 10.1038/s41467-023-39951-y (PMC10374558; doi:10.1038/s41467-023-39951-y)
Supplement: Supplementary file 1 — Supplementary Information [file 41467_2023_39951_MOESM1_ESM.pdf]

Supplementary Information for

Precise cooperative sulfur placement leads to semi-crystallinity and selective depolymerisability in CS_2/oxetane copolymers

Table of contents

Supplementary Notes 1: Supplementary methods ... 3
Supplementary Notes 2: Catalyst synthesis and characterisation ... 5
Supplementary Notes 3: CS_2/oxetane ROCOP with LCrK ... 7
Supplementary Notes 4: CS_2/oxetane ROCOP with other catalysts ... 16
Supplementary Notes 5: CS_2/OX^{Me} ROCOP with LCrK ... 22
Supplementary Notes 6: CS_2/OX^{OR} ROCOP with LCrK ... 27
Supplementary Notes 7: CS_2 ROCOP of epoxides with LCrK ... 42
Supplementary Notes 8: De- and repolymerisation experiments ... 49
Supplementary Notes 9: Mechanistic hypothesis explaining links and by-products ... 58
Supplementary Notes 10: Density functional theory ... 59
Supplementary References ... 60

Supplementary Notes 1: Supplementary methods

Solvents and reagents were obtained from commercial sources and used as received unless stated otherwise. Oxetane in particular was obtained from Sigma Aldrich as well as Fisher Scientific. NMR spectra were recorded by using a Jeol JNM-ECA 400II, Bruker Advance 600 and 700 MHz spectrometer. ^{1}H and $^{13}C\{^{1}H\}$ chemical shifts are referenced to the residual proton resonance of the deuterated solvents.

Oxetanes, CS_2, PO and CHO were dried over calcium hydride at room temperature for 3 days followed by vacuum transfer (for CHO fractionally distilled under static vacuum) and three freeze pump thaw degassing cycles and stored inside an argon filled glovebox prior to use. Oxetane employed in table 1 run #8, #9 and #10 was additionally dried over elemental sodium. PPNOAc, KOAc@18-crown-6 and A were prepared following literature methods.[1–3] Cyclohexanediamine was dried over 4Å molecular sieves and degassed prior to use. **A** and **L'** were synthesized according to the literature procedure.[2,4] DCM was dried by using an MBraun Solvent Purification System MB-SPS 800 filled with Al_2O_3 followed by drying over 4Å molecular sieves. THF was dried over K/benzophenone under argon followed by drying over 4Å molecular sieves. Benzylalcohol and mesitylene were dried over 4Å molecular sieves. All other reagents were used as received if not stated otherwise.

High-resolution mass spectra were obtained using a Waters UPLC-Synapt G2-S HDMS. Infrared spectra were measured using a Thermo-Nicolet Nexus 670 FTIR spectrometer with DuraSampl IR accessory in total reflection at room temperature. TGA data was measured using a Netzsch TG 209 (heating rate 10 K/min). DSC was measured on a Netzsch 204 F1 “Phoenix”. PXRD was measure in a Malvern Panalytical Empyrean diffractometer equipped with a PIXcel 1D detector using Cu-K_α radiation (λ = 1.54 Å) at room temperature in reflection mode. Films were prepared by hot-pressing 500 mg of polymer between two aluminium plates covered with Teflon sheets heated with two LLG hotplates (held in place by a 5 kg weight put on top) at 110ºC for 5 minutes followed by hardening at 70ºC for 2h. Storage modulus (E′, MPa), loss modulus (E″, MPa) and tan δ (E″/E′) measurements were performed on a DMA 242 C (NETZSCH) in tension mode on rectangular specimens (l = 7.650 mm, w = 3.810 mm, t ≈ 0.719 mm). The stamps were tightened to 10 Nm with a torque wrench. Temperature-ramp experiments were performed at 1 Hz between –100 and 90 °C at a heating rate of 1 K/min. Temperatures below ambient conditions were accessed via liquid nitrogen CC 200 supply system (NETZSCH). Post-run analysis was performed on a NETZSCH Proteus Thermal Analysis software. Uniaxial tensile testing was performed on a Wick/Roell Z010 instrument (ZwickRoell GmbH & Co., KG, Germany, 500 N load cell class 0.5, extensimeter multixtens class 0.5).

The molecular mass and polydispersity of the polymers were determined by a Waters 1515 gel permeation chromatography (GPC) instrument equipped with two linear PLgel columns (Mixed-C) following a guard column and a differential refractive index detector using tetrahydrofuran as the eluent at a flow rate of 1.0 mL/min at 30 °C and a series of narrow polystyrene standards for the calibration of the columns. Each polymer sample was dissolved in HPLC-grade THF (6 mg/mL) and filtered through a 0.20 μm porous filter frit prior to analysis.

Molecular periodic calculations for the systems in figure 5 were performed with the CRYSTAL17 program,[8] using the B3LYP DFT functional in combination with Grimme type

dispersion correctionand employing the Gaussian-type atomic basis set cc-pVDZ.[9–12] The first Brillouin zone was sampled using an 6×6×6 Monkhorst-Pack grid. To facilitate convergence, the Coulomb and exchange integral thresholds were sufficiently tightened with the TOLINTEG keyword to values of 8, 8, 8, 8 and 16. For the calculations of dimers of oligomer chains, the calculations were performed at the same level of theory but with the Gaussian programAIM analysis according to Bader was performed with the Multiwfn program.[13,14]

Supplementary Notes 2: Catalyst synthesis and characterisation

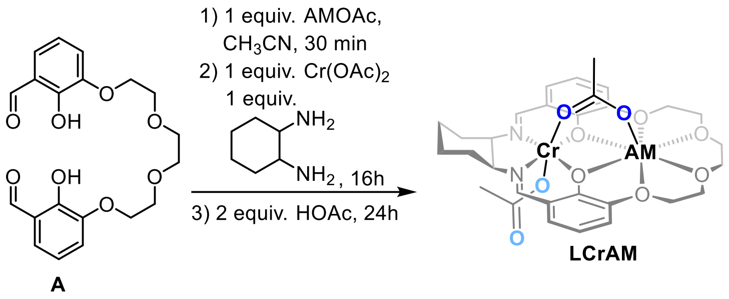

Supplementary Figure 1: Synthesis of **LCrAM**.

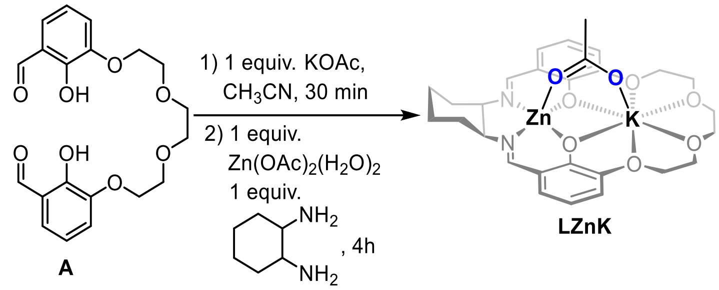

Supplementary Figure 2: Synthesis of **LZnK**.

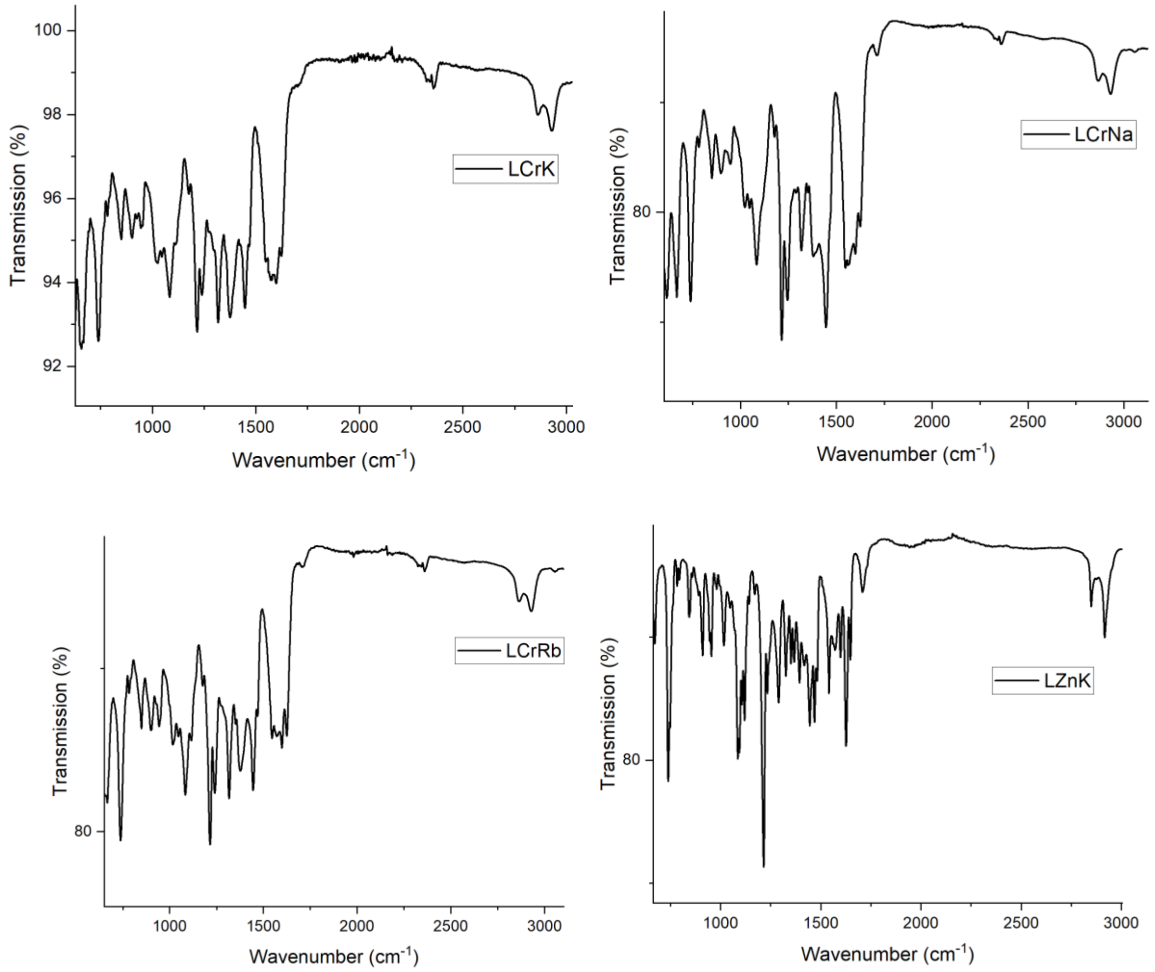

Supplementary Figure 3: IR spectra of **LCrNa**, **LCrK**, **LCrRb** and **LZnK**.

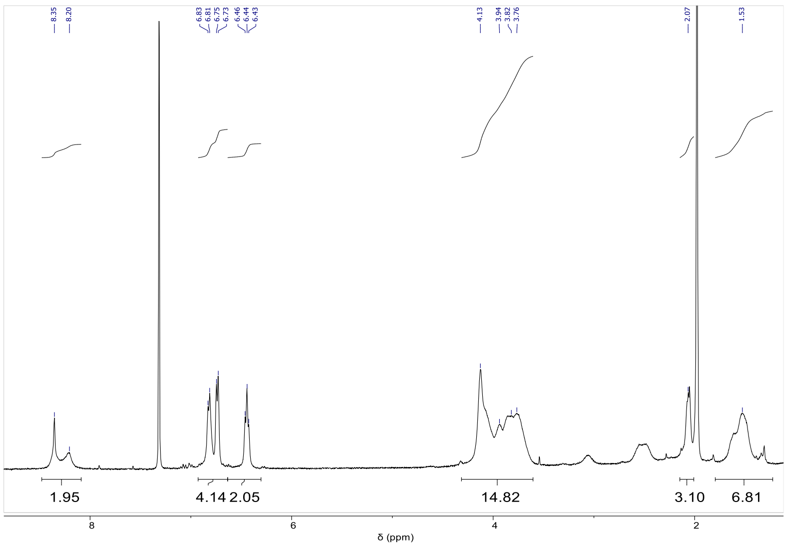

Supplementary Figure 4: ^{1}H NMR spectrum (400 MHz, $CDCl_3$, 25°C) spectrum of **LZnK.** Residual CH_3CN at 2.5 ppm.

Supplementary Notes 3: CS_2/oxetane ROCOP with LCrK

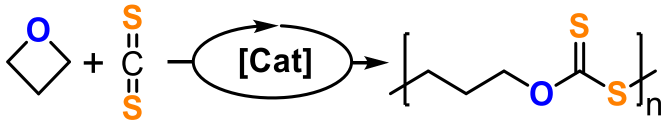

Supplementary Figure 5: Synthesis of CS_2/OX copolymer.

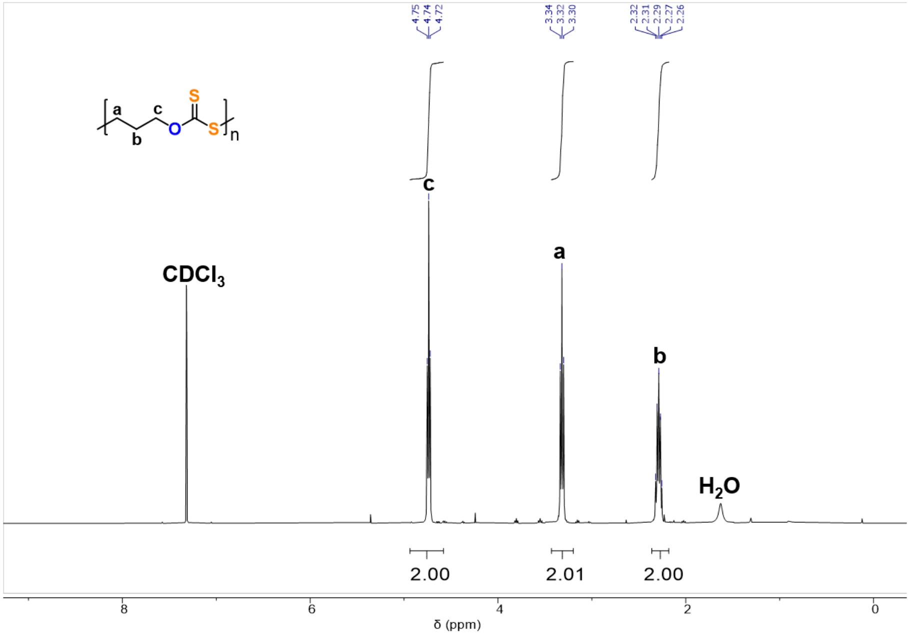

Supplementary Figure 6: ^{1}H NMR spectrum (400 MHz, $CDCl_3$, 25°C) of the polymer corresponding to table 1, run #1.

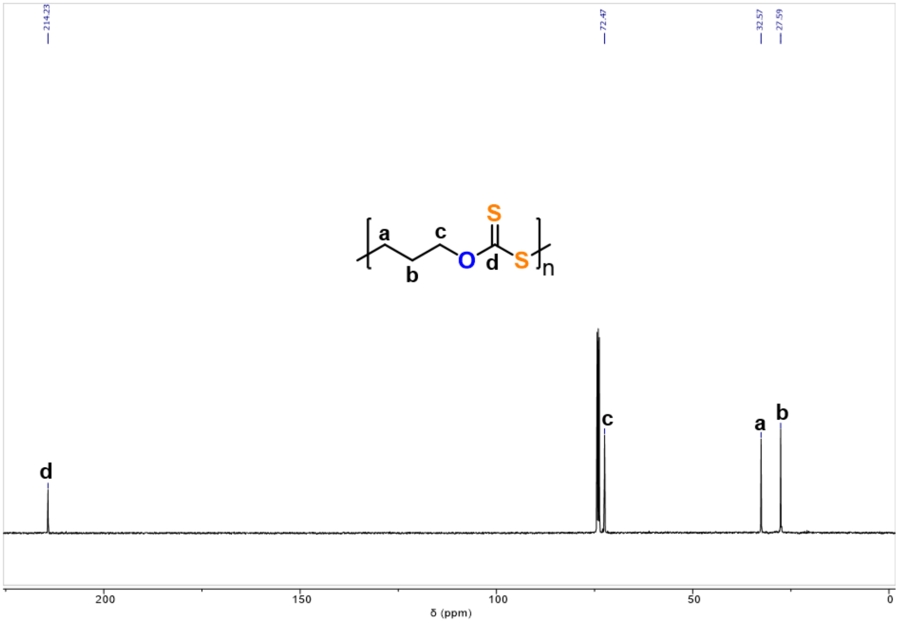

Supplementary Figure 7: ^{13}C NMR spectrum (126 MHz, $CDCl_3$, 25°C) spectrum of the polymer corresponding to table 1, run #1.

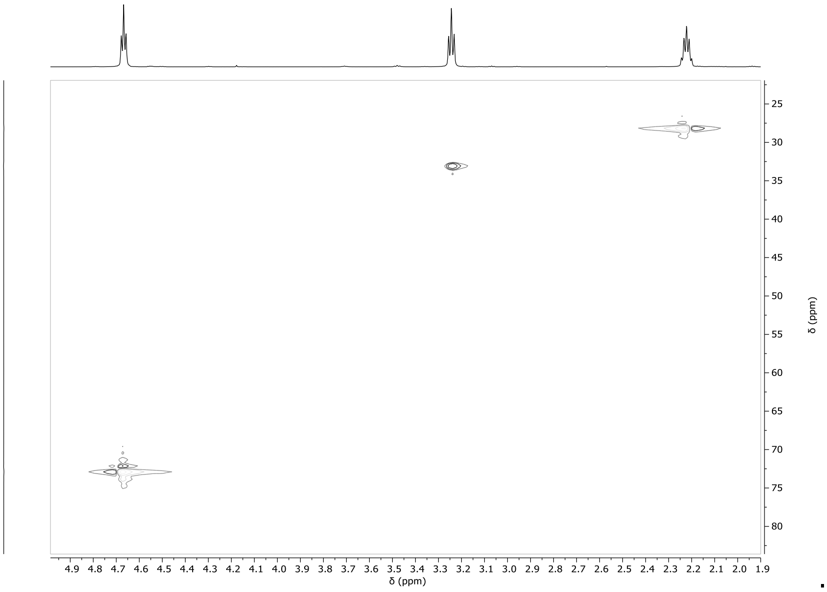

Supplementary Figure 8: ^{1}H-^{13}C HSQC NMR spectrum (d_2-TCE, 25°C) spectrum of the polymer corresponding to table 1, run #1.

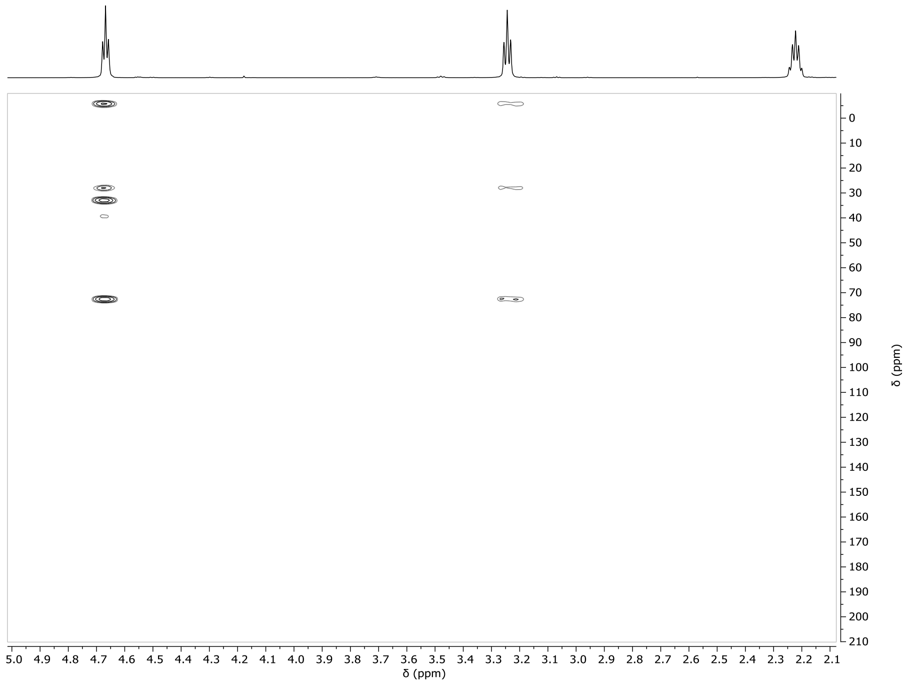

Supplementary Figure 9: ^{1}H-^{13}C HMBC NMR spectrum (d_2-TCE, 25°C) spectrum of the polymer corresponding to table 1, run #1 Signals above 210 ppm folded.

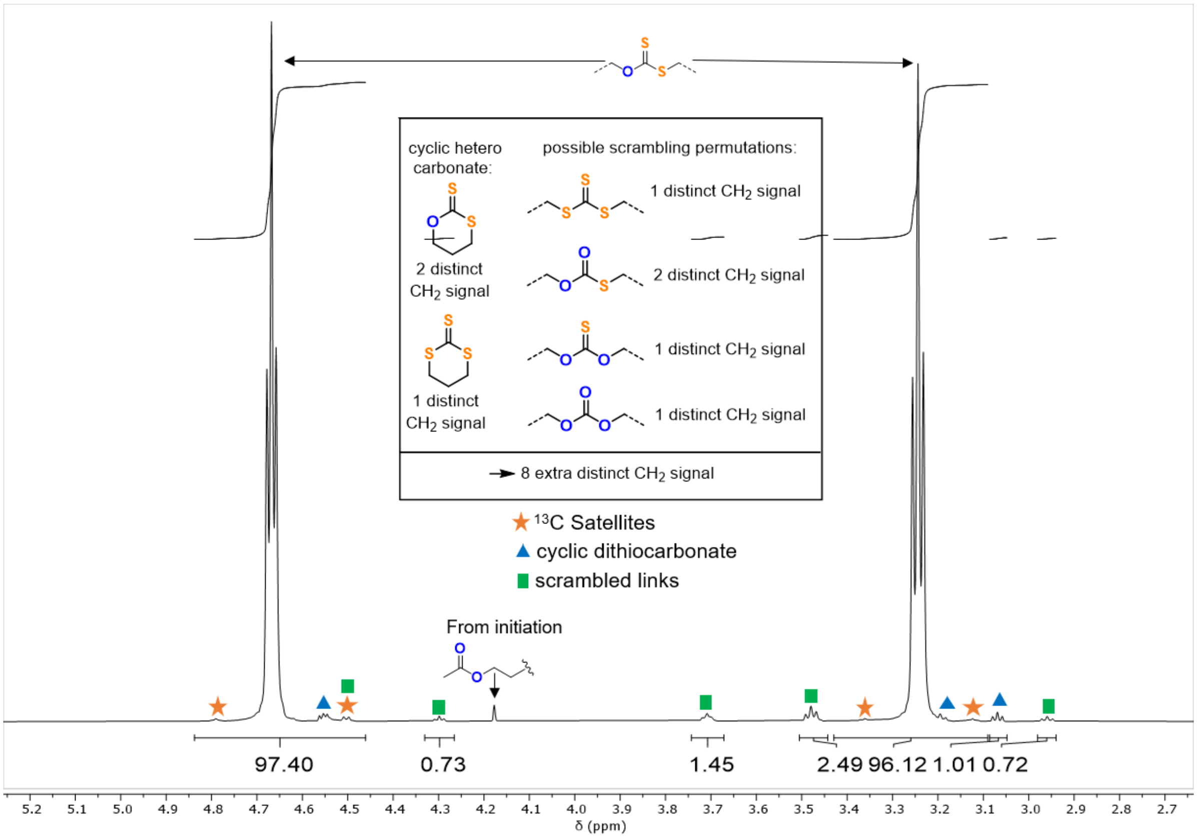

Supplementary Figure 10: Example ^{1}H NMR spectrum (600 MHz, d_2-TCE, 70°C) spectrum of the reaction mixture (Table 1, run #1), DCM was used to solubilize the reaction mixture out of the vial resulting in trace ammounts. Assignment according to 2D NMR spectra of scrambled polymers of Section S5 and in reference to [5].

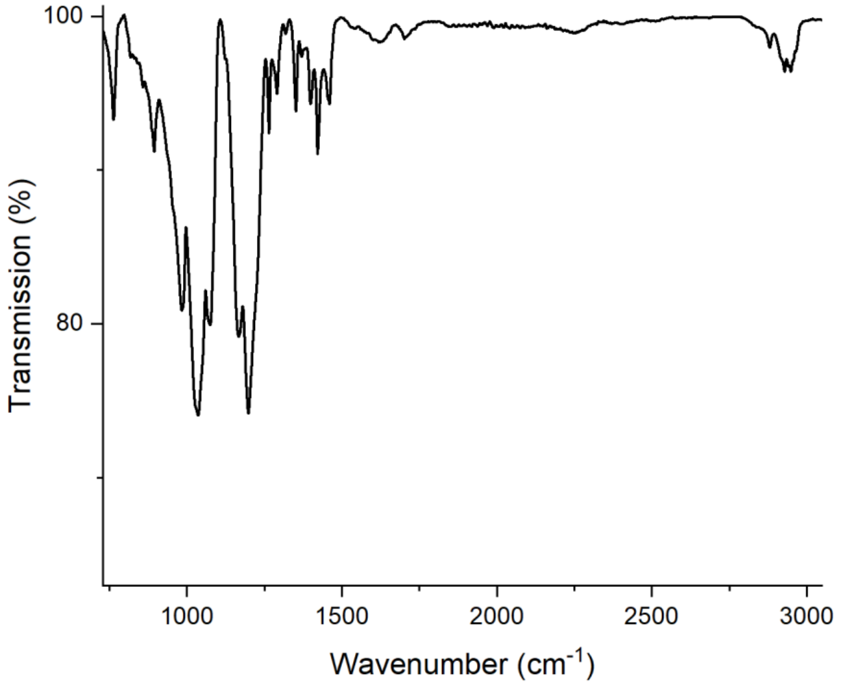

Supplementary Figure 11: IR spectrum of copolymer corresponding to table 1, run #1.

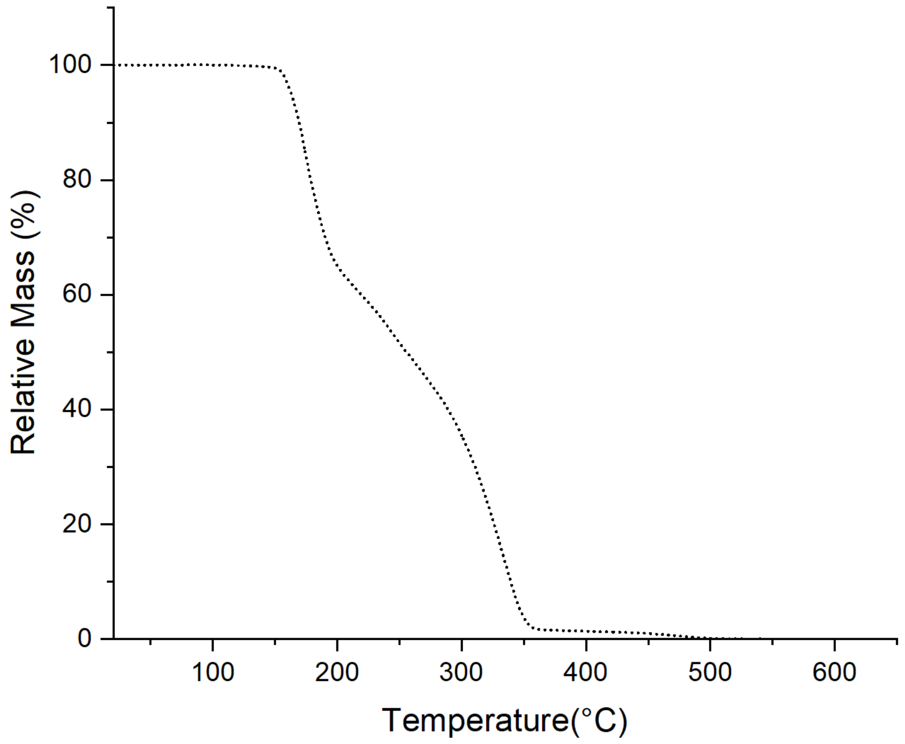

Supplementary Figure 12: TGA data of copolymer corresponding to table 1, run #1. $T_{d,5\%}$ = 163.2 ºC.

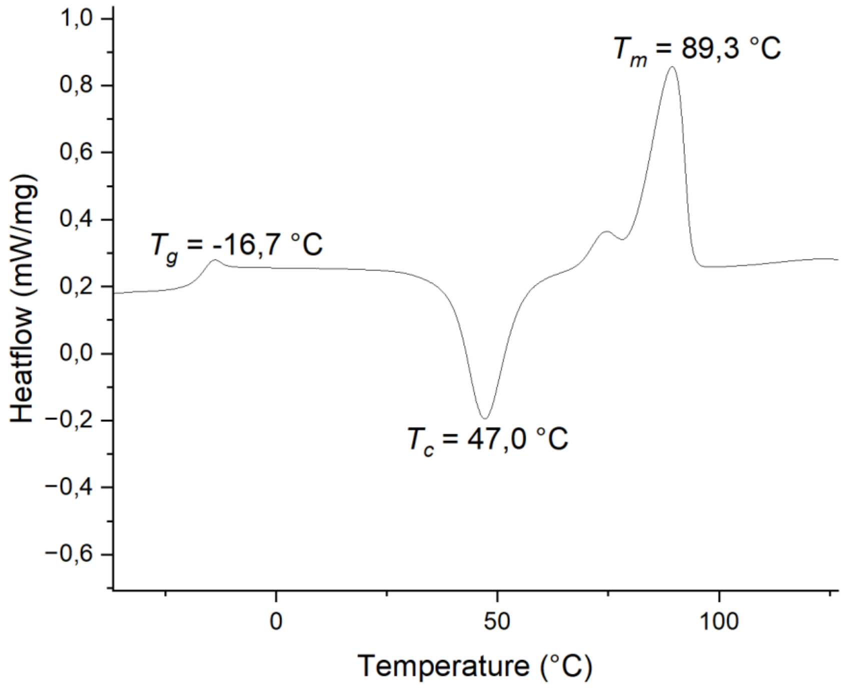

Supplementary Figure 13: DSC data from second heating cycle of copolymer corresponding to table 1, run #1.

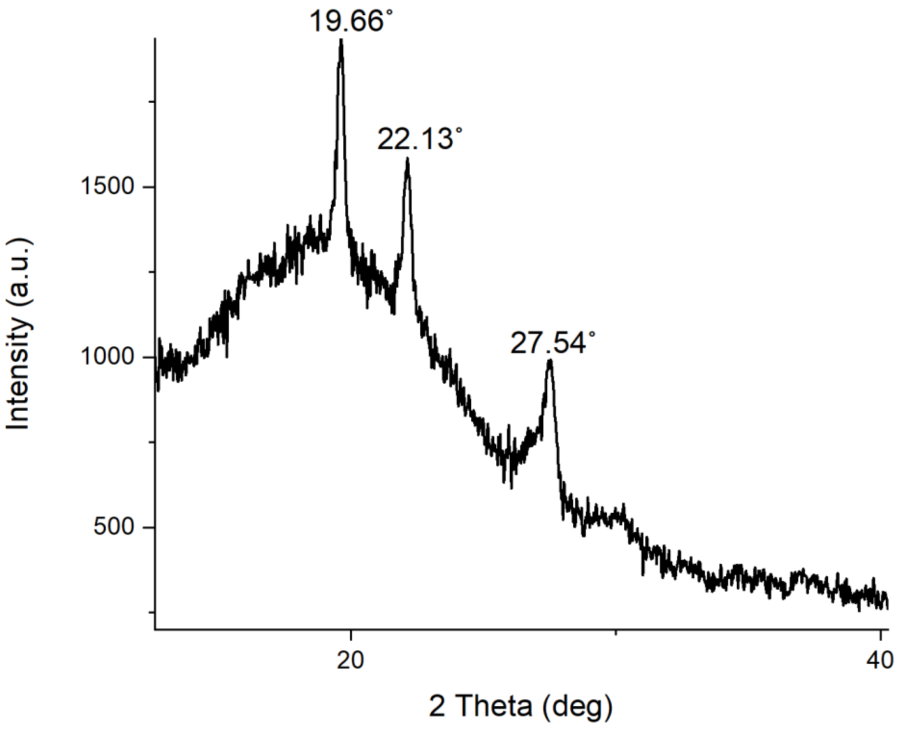

Supplementary Figure 14: PXRD data of copolymer corresponding to table 1, run #1.

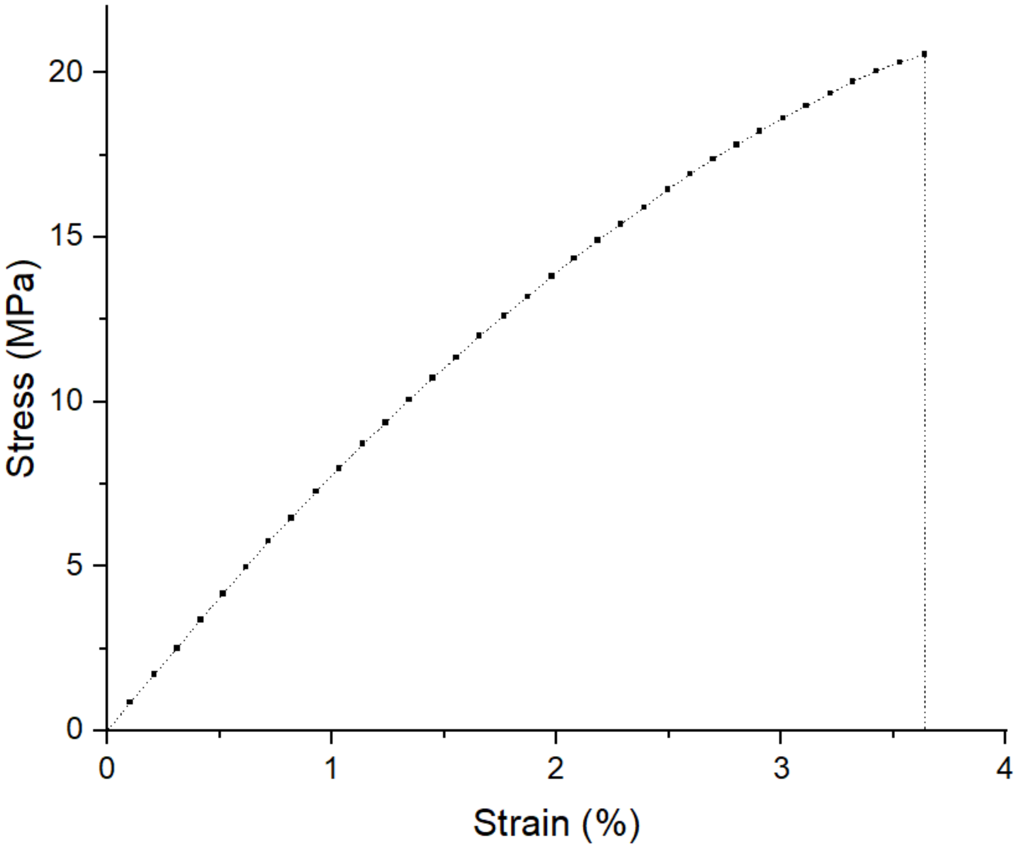

Supplementary Figure 15: Stress-strain curve of film prepared from copolymer corresponding to table 1, run #9.

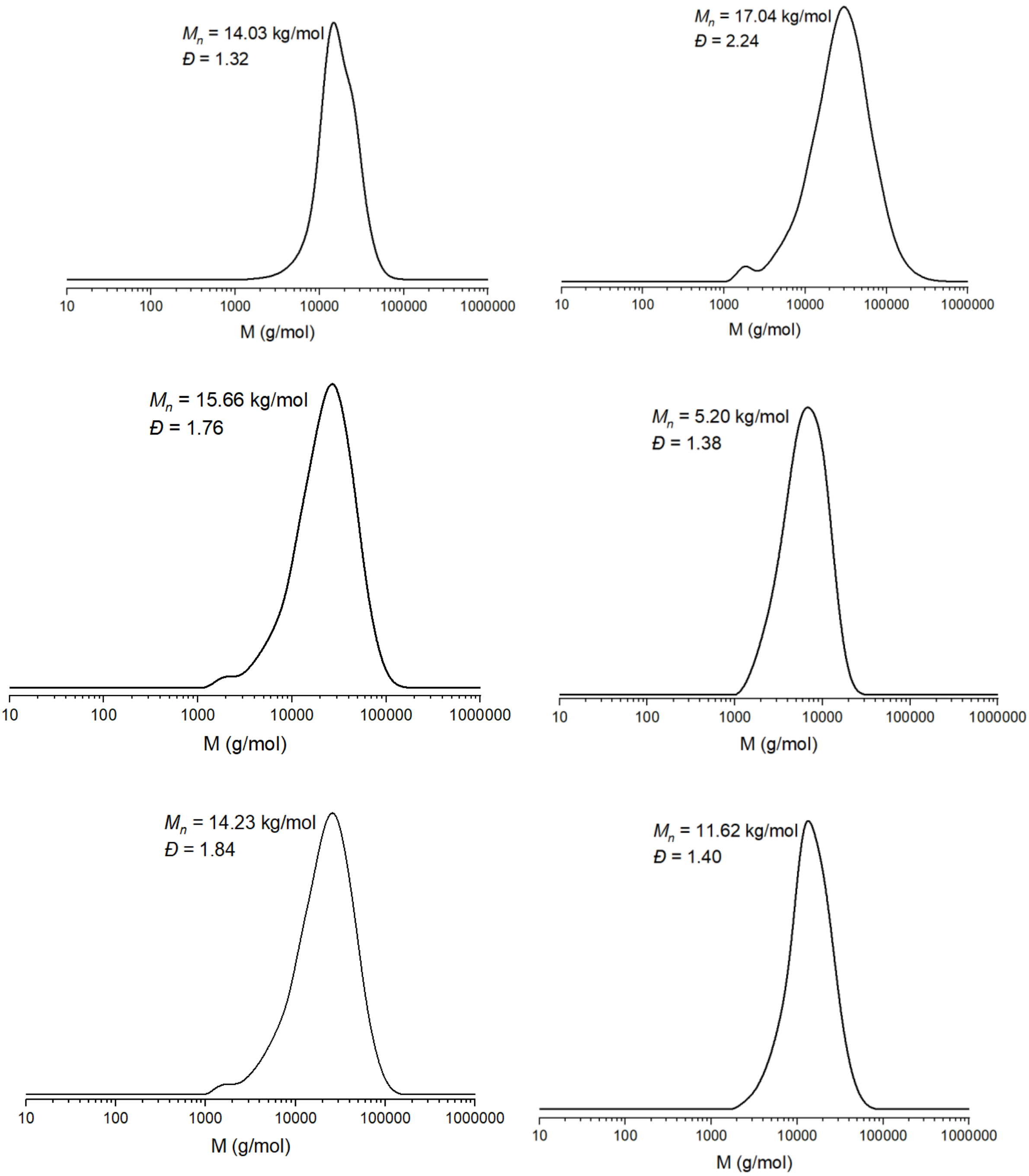

Supplementary Figure 16: GPC traces corresponding to table 1 run #1-#6.

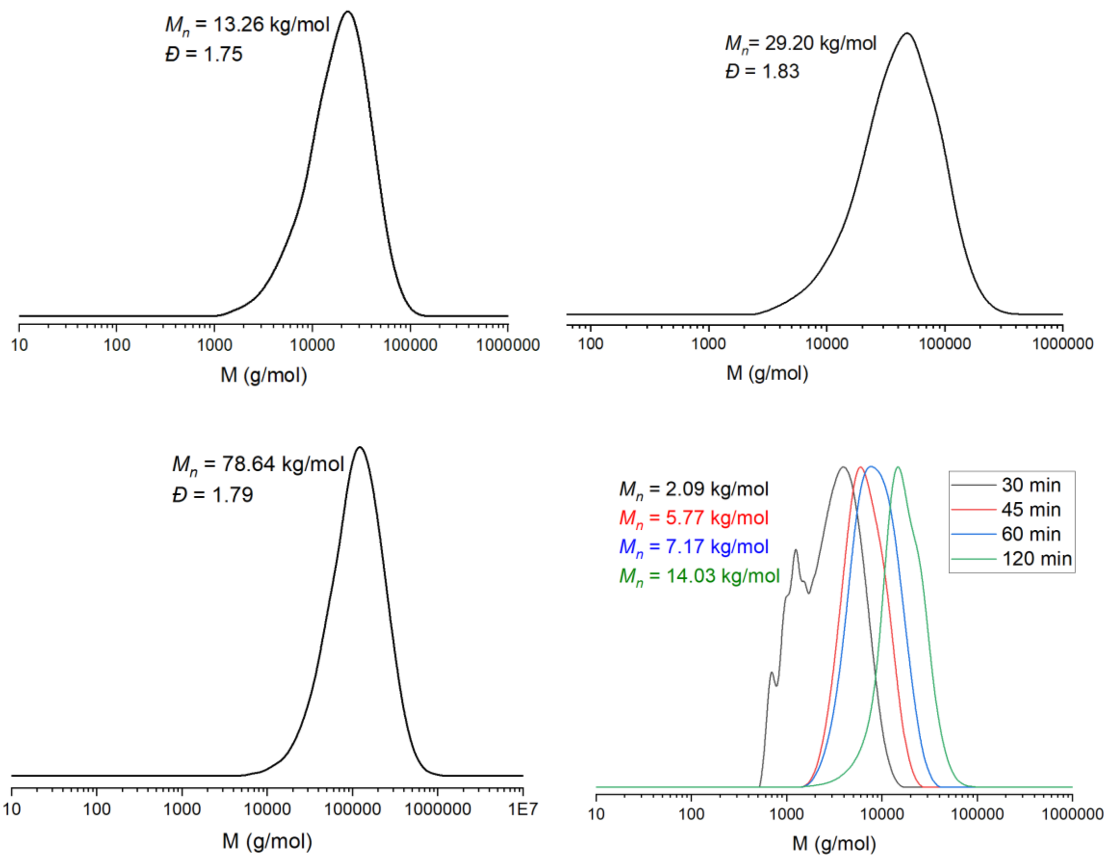

Supplementary Figure 17: GPC traces corresponding to table 1 run #7, #8, #9 and M_n versus reaction time development corresponding to table 1 run #1.

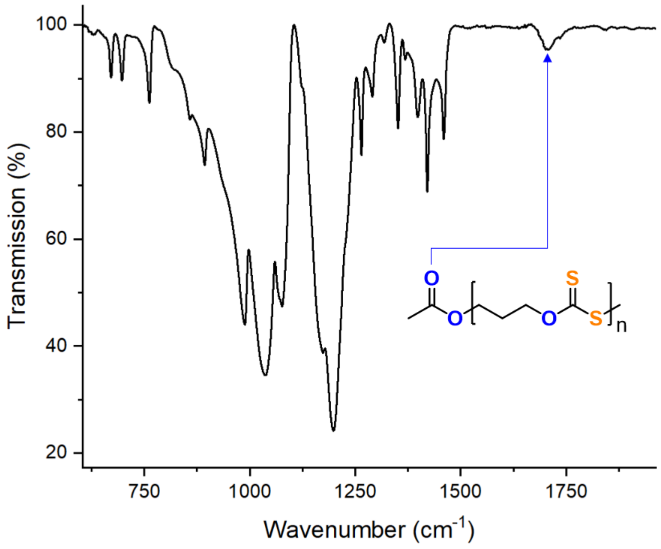

Supplementary Figure 18: IR spectrum of copolymer corresponding to table 1, run #4.

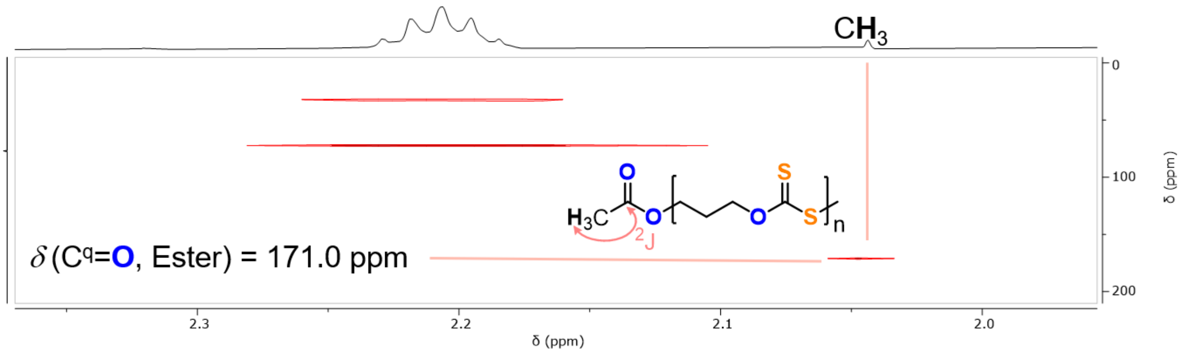

Supplementary Figure 19: Zoom into the ^{1}H-^{13}C HMBC NMR spectrum ($CDCl_3$, 25°C) spectrum of copolymer corresponding to table 1, run #4.

End-group analysis by ^{31}P NMR spectroscopy: A reported procedure by Marchessault (*Macromolecules* 1997, 30, 327-329) for the analysis of protic end-groups was followed. A polymer sample (20 mg of table 1 run #3) and a stock solution (40 µL, made of bisphenol A (400 mg), chromium(III)acetylacetonate (5.5 mg), and pyridine (10 mL)), in $CDCl_3$ (0.5 mL), were mixed in an NMR tube. Excess 2-chloro-4,4,5,5-tetramethyldioxaphospholane (40 µL) was then added to the NMR tube which was then shaken. The mixture was allowed to react for 30 min before analysis by ^{31}P NMR.

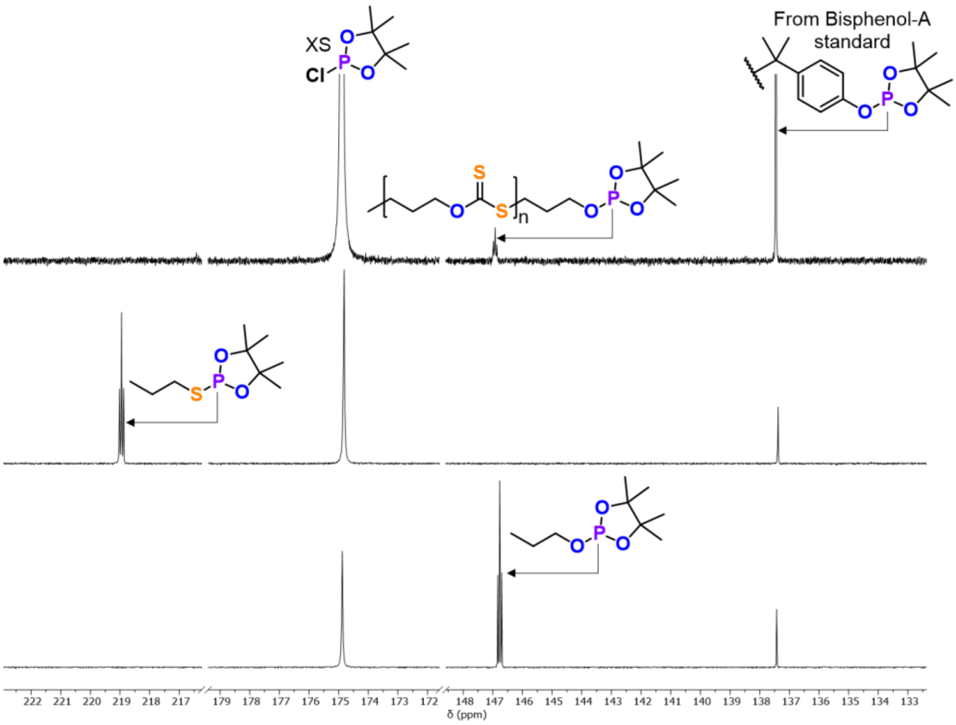

Supplementary Figure 20: ^{31}P NMR (162 MHz, $CDCl_3$, 25°C) end-group analysis of (top) CS_2/OX copolymer (table 1, run #3) and (middle, bottom) control experiments with propanol and propanthiol.

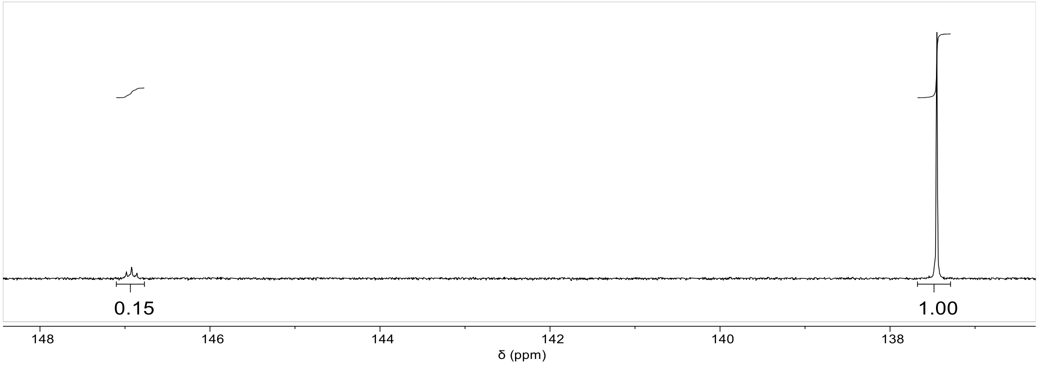

Supplementary Figure 21: Relative integrals in the ^{31}P (162 MHz, $CDCl_3$, 25°C) NMR end-group analysis of the copolymer (table 1, run #3) corresponding to 2.10 μmol protic end-groups in 20 mg polymer. Note that (assuming M_n = 15660 g/mol as per GPC and α-OAc,ω-OH-functional chains from acetate initiation followed by uniform propagation) 1.27 μmol polymer-chains would have been employed, hence more OH chain ends are formed than exclusive formation of α-OAc,ω-OH-functional chains would imply, which we infer to stem from the formation of some α,ω-OH-bifunctional telechelic chains.

Supplementary Notes 4: CS_2/oxetane ROCOP with other catalysts

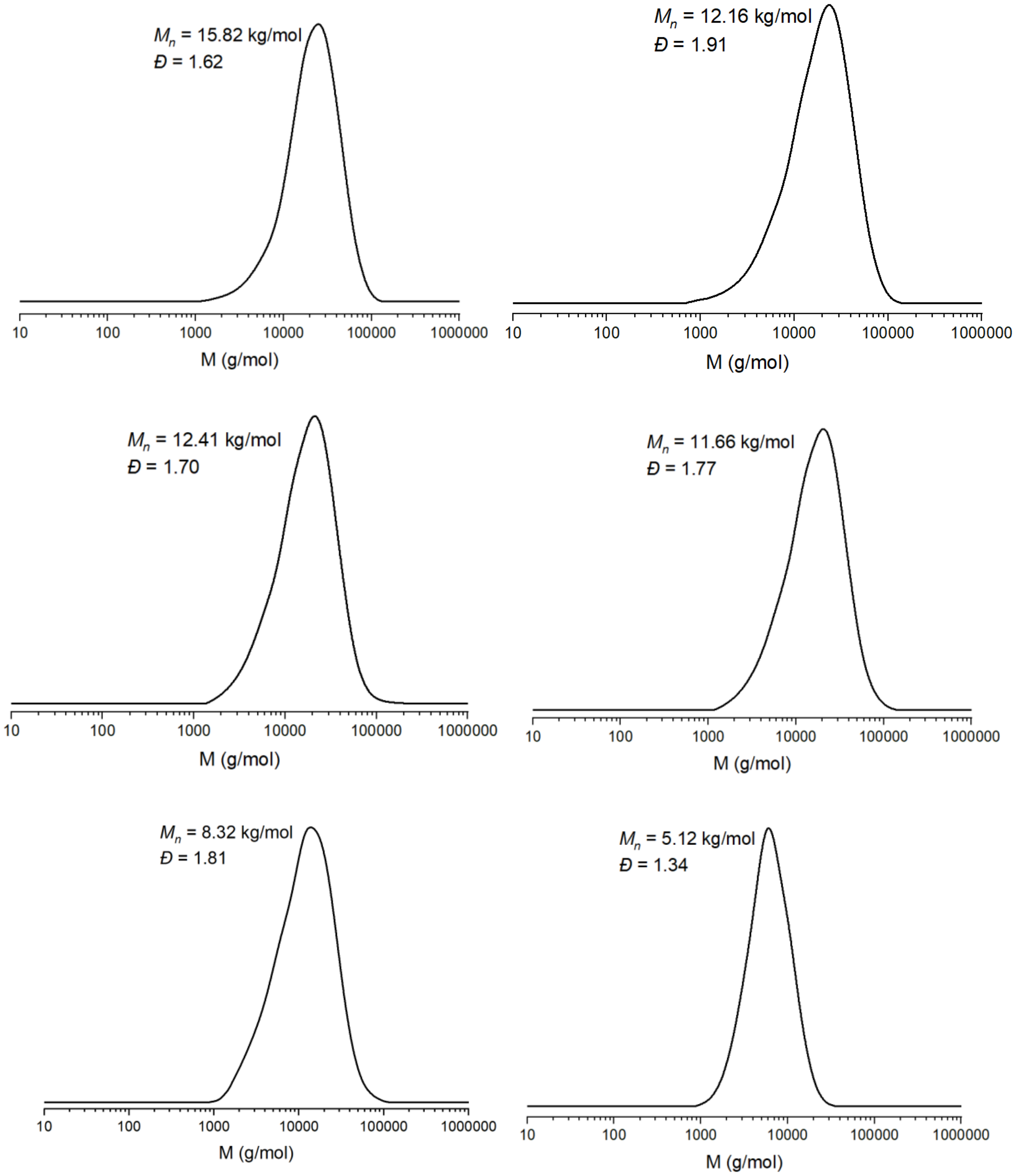

Supplementary Figure 22: GPC traces corresponding to table 2, run #1-#8.

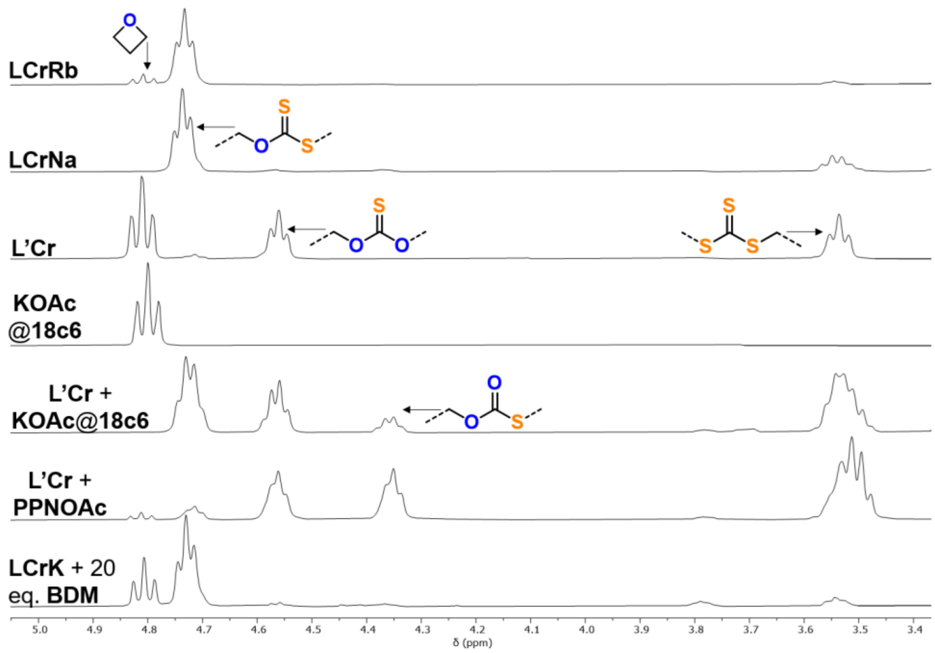

Supplementary Figure 23: Overlaid ^{1}H NMR spectrum (400 MHz, $CDCl_3$, 25°C) spectra of reaction mixtures corresponding to table 2. BDM denotes benzenedimethanol.

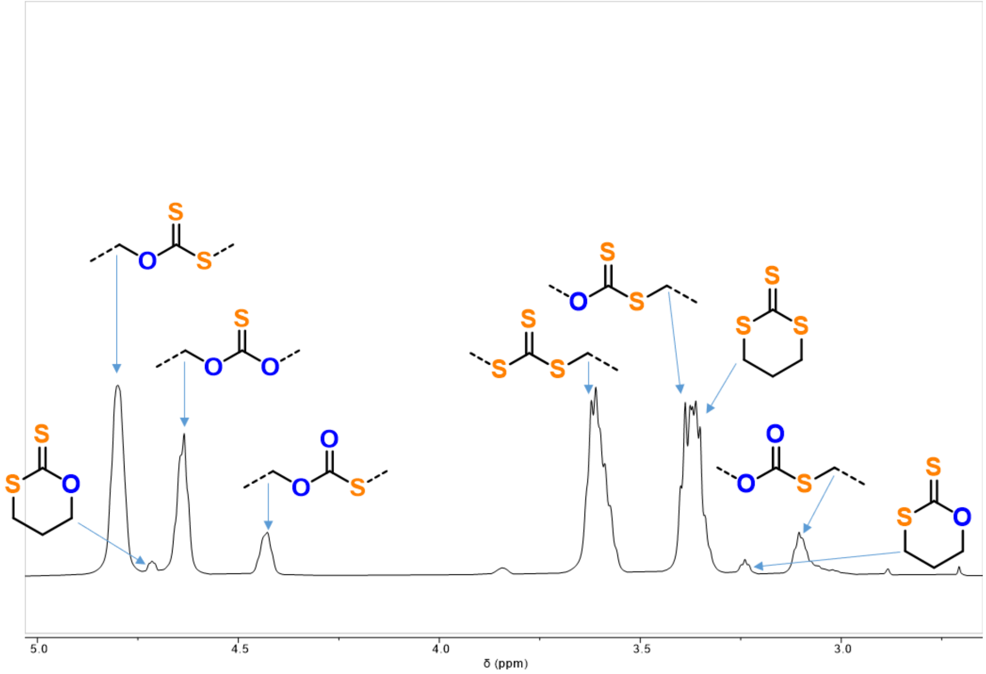

Supplementary Figure 24: Zoom into the heterocarbonate region of the ^{1}H NMR spectrum (600 MHz, $CDCl_3$, 25°C) spectrum of the product mixture corresponding to table 2, run #4. Assignment by HMBC (*vide infra*) and in reference to [5].

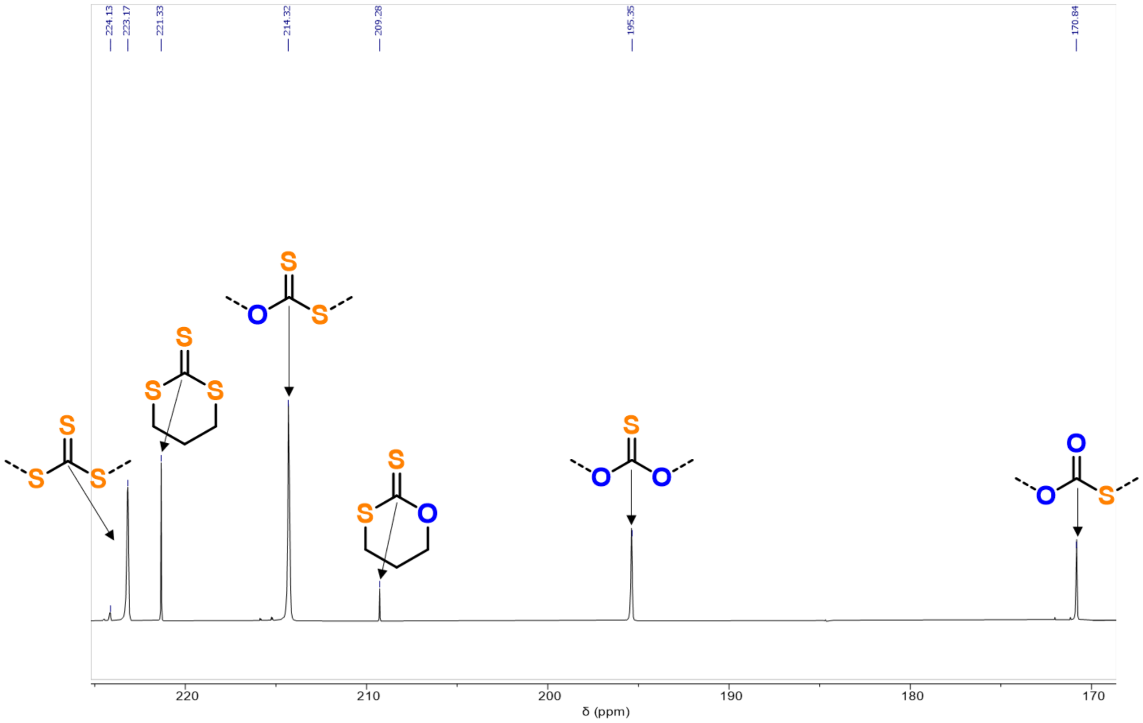

Supplementary Figure 25: Zoom into the heterocarbonate region of the ^{13}C NMR spectrum (126 MHz, $CDCl_3$, 25°C) spectrum of the product mixture corresponding to table 2, run #4. Assignment by HMBC (*vide infra*) and in reference to [5].

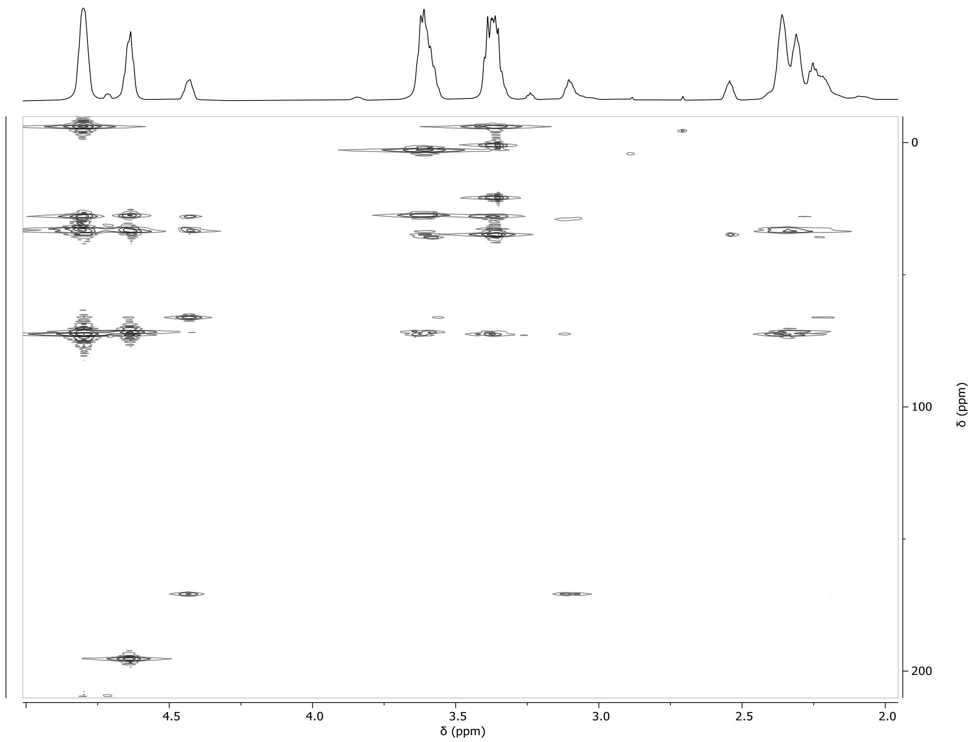

Supplementary Figure 26: ^{1}H-^{13}C HMBC NMR spectrum ($CDCl_3$, 25°C) spectrum of the product mixture corresponding to table 2, run #4. Signals above 210 ppm folded.

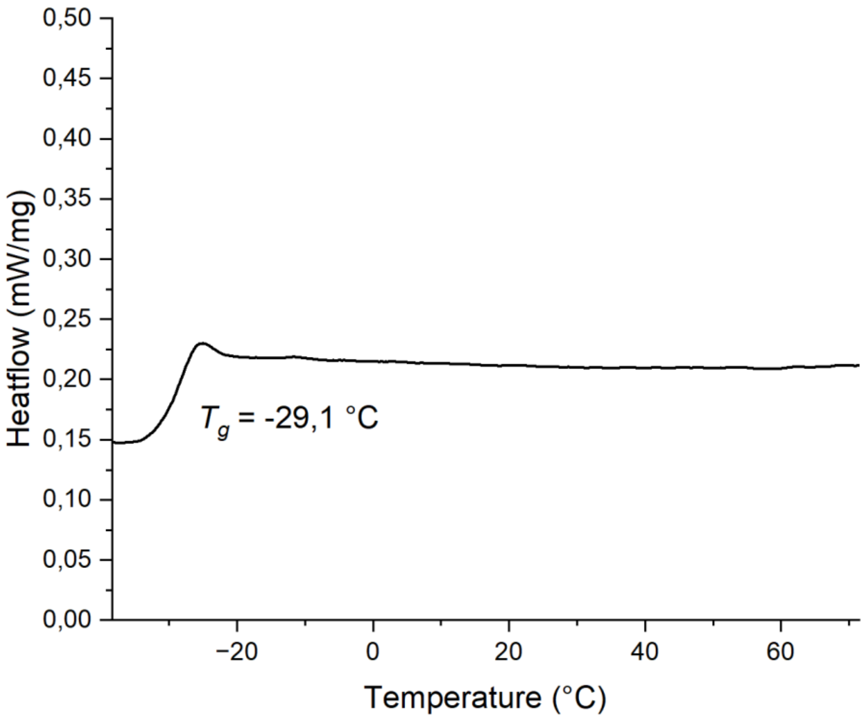

Supplementary Figure 27: DSC data from second heating cycle of copolymer corresponding to table 2, run #7.

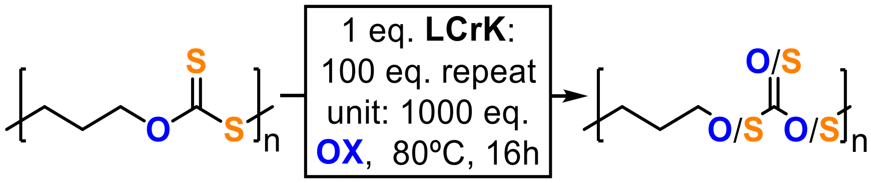

Supplementary Figure 28: Scrambling experiment.

Inside an argon filled glove-box, **LCrK** (1 eq.), poly(trimethylene dithiocarbonate) (100 eq. repeat unit prepared as per table 1, run #1) and oxetane (1000 eq.) were added to a flame dried vial equipped with a flame dried stirrer bar and sealed with a melamine cap containing a Teflon inlay. The vial was brought outside the glovebox and placed in a pre-heated aluminium block at 80ºC for 16h. Afterwards, oxetane was removed by air-drying, the reaction mixture was taken up in $CDCl_3$ and analysed by NMR. Furthermore, the products were analysed by GPC.

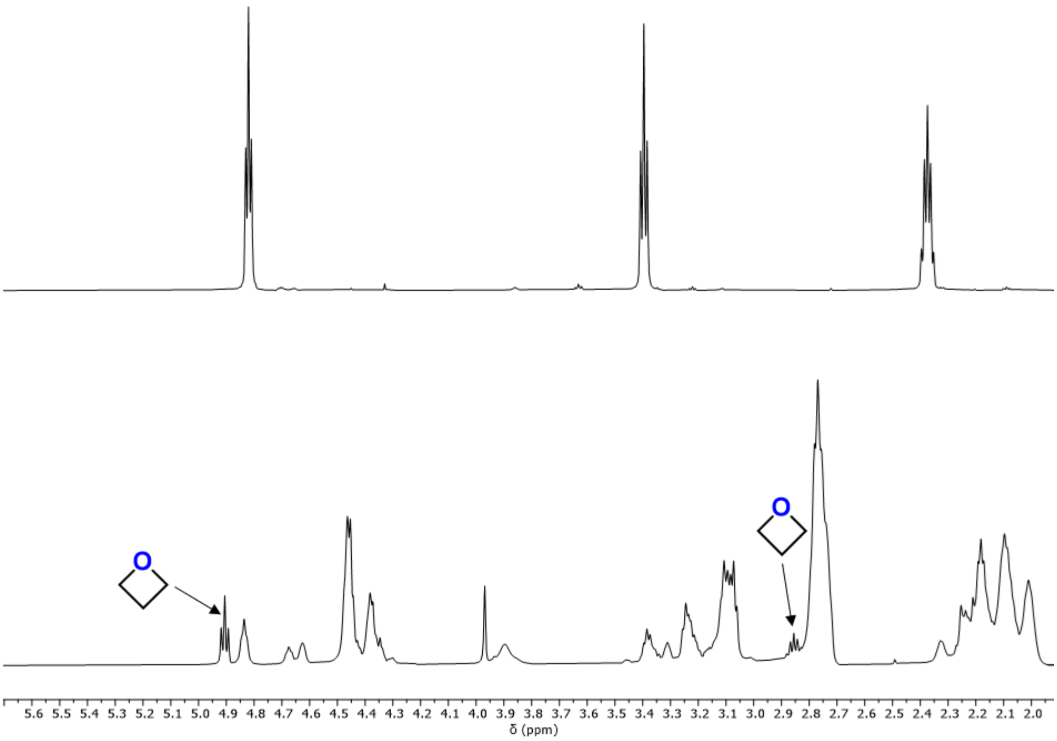

Supplementary Figure 29: ^{1}H NMR (400 MHz, $CDCl_3$, 25°C) spectrum of polymer (top) before and (bottom) after scrambling experiment as outlined above. Residual oxetane from reaction medium marked.

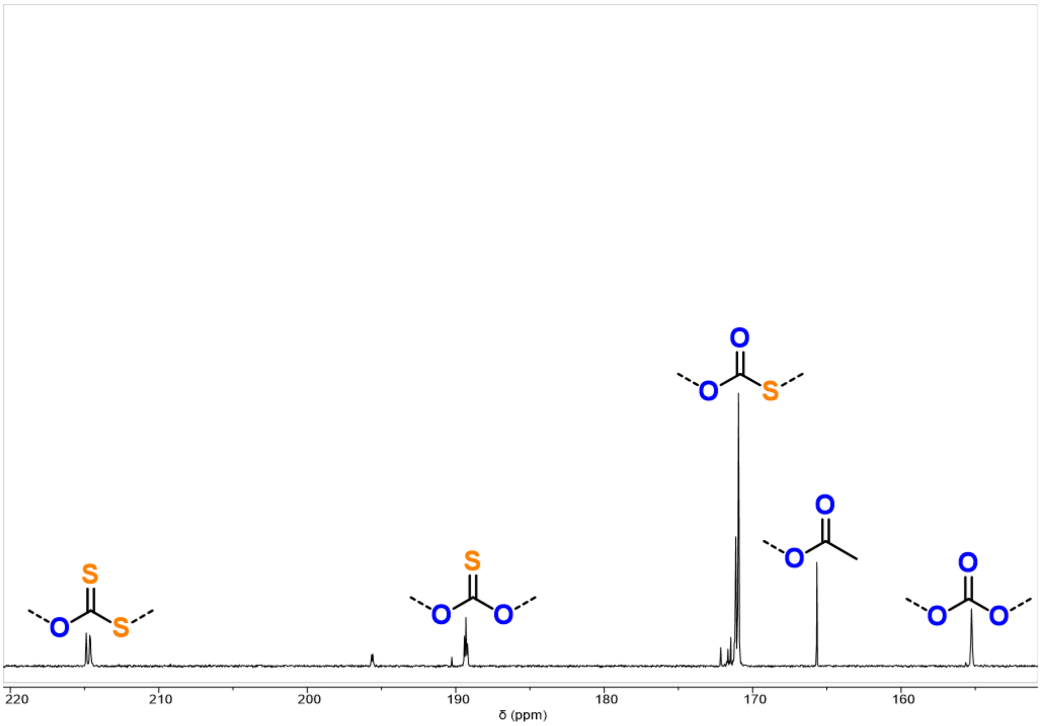

Supplementary Figure 30: Zoom into the heterocarbonate region of the ^{13}C NMR spectrum (126 MHz, $CDCl_3$, 25°C) of polymer after scrambling experiment. Ester units originating from OAc colligated initiators of **LCrK.**

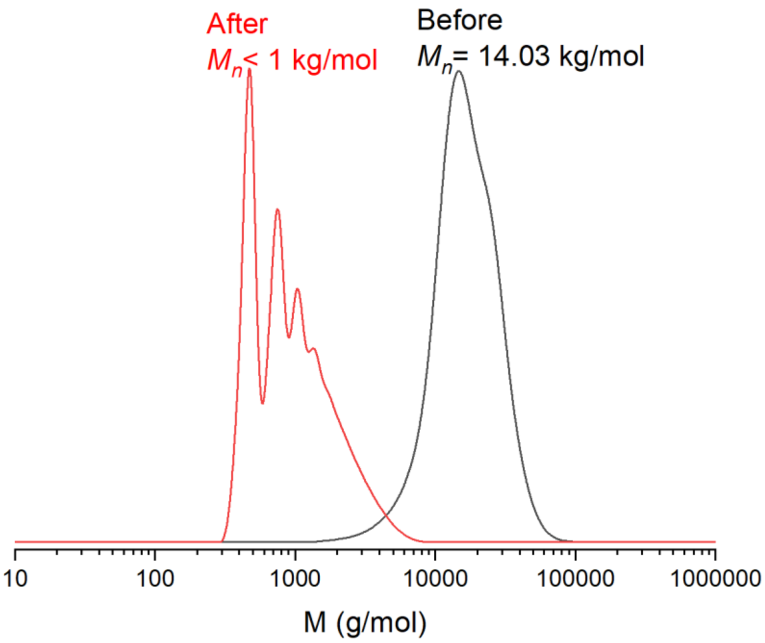

Supplementary Figure 31: Overlaid GPC traces before and after scrambling experiment outlined above.

Supplementary Notes 5: CS_2/OX^{Me} ROCOP with LCrK

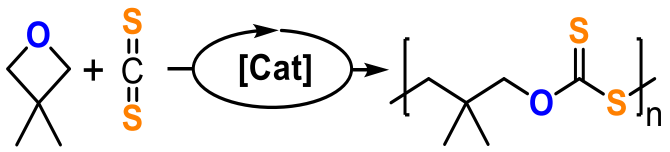

Supplementary Figure 32: Synthesis of CS_2/OX^{Me} copolymer.

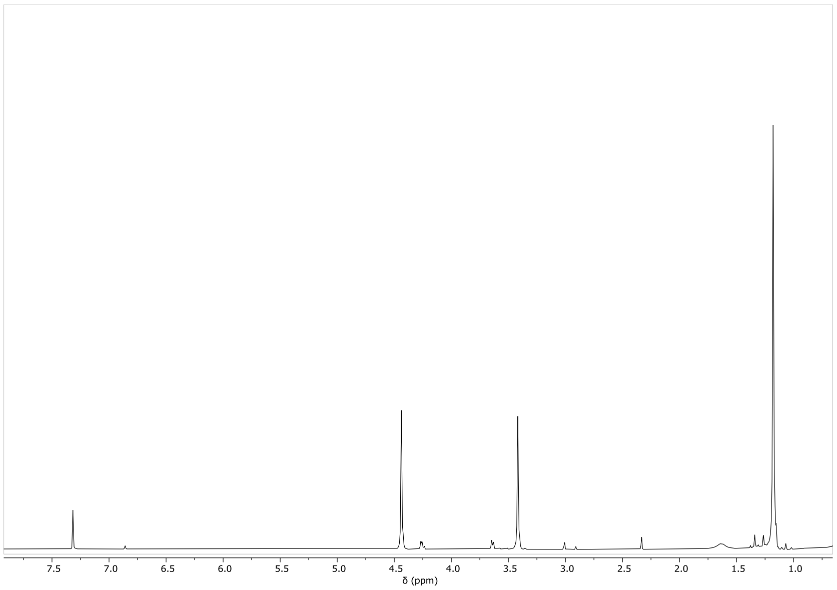

Supplementary Figure 33: ^{1}H NMR spectrum (400 MHz, $CDCl_3$, 25°C) spectrum of the crude reaction mixture showing the absence of cyclic carbonate byproducts corresponding to table 3, run #1.

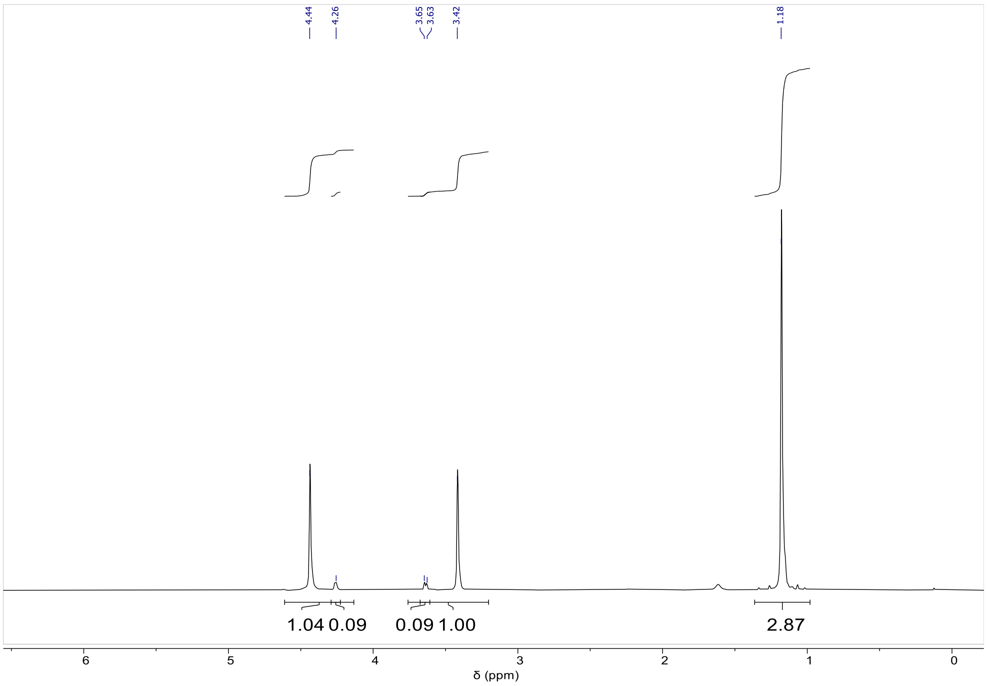

Supplementary Figure 34: ^{1}H NMR spectrum (400 MHz, $CDCl_3$, 25°C) of the isolated polymer corresponding to table 3, run #1.

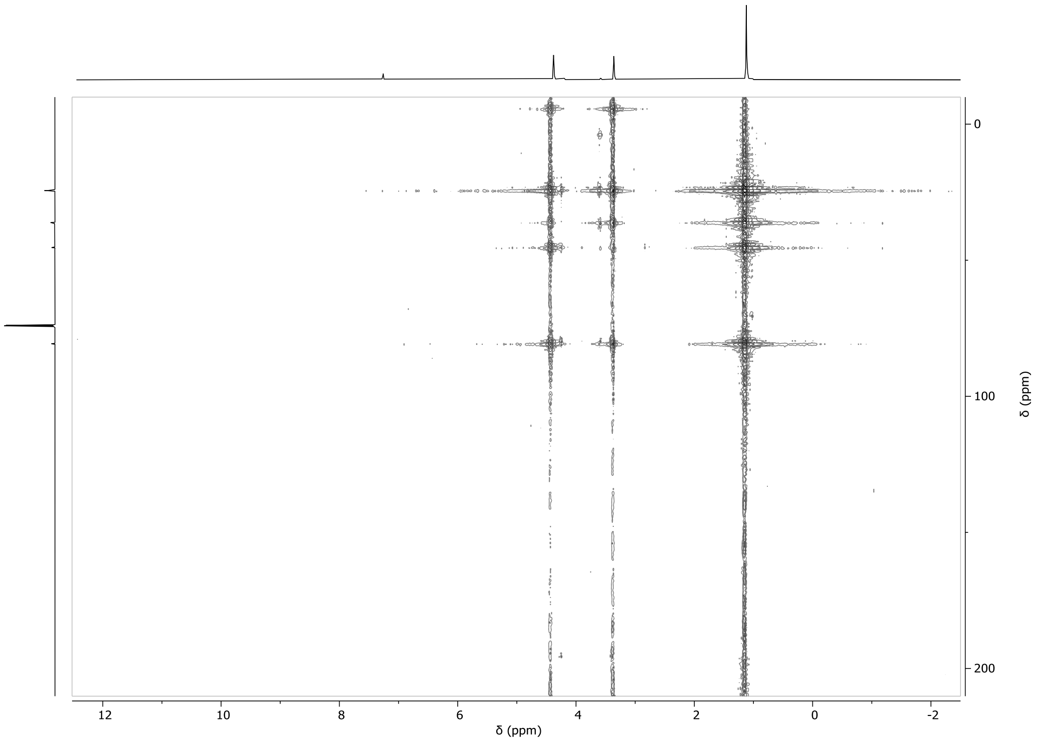

Supplementary Figure 35: ^{1}H-^{13}C HMBC NMR spectrum (d_2-TCE, 70°C) spectrum of the isolated polymer corresponding to table 3, run #1. Signals above 210 ppm folded.

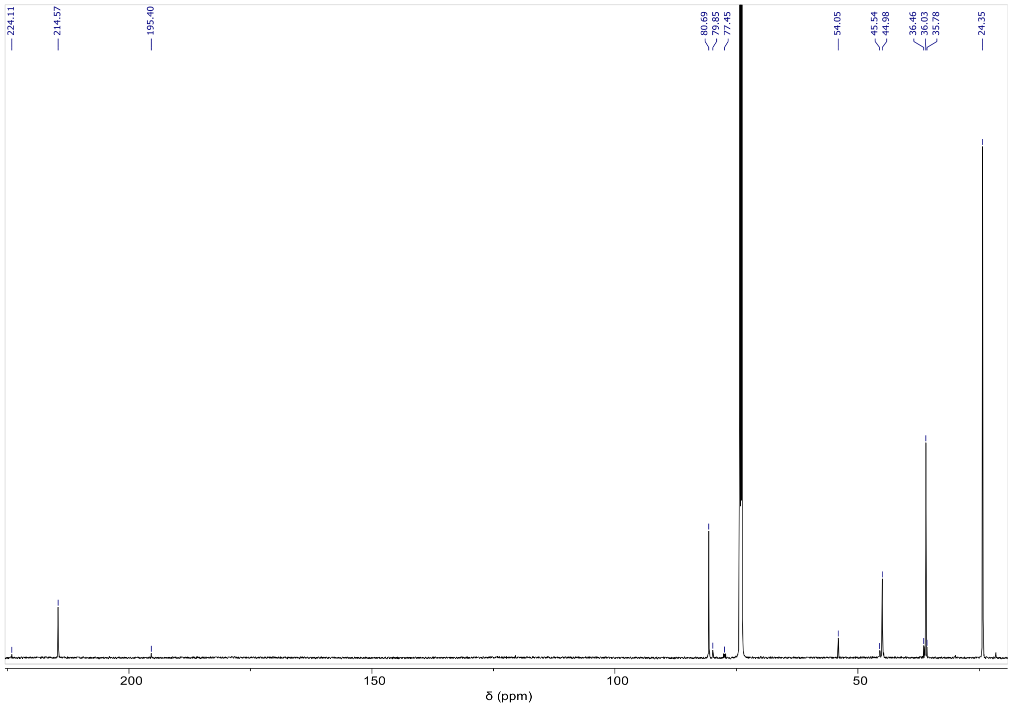

Supplementary Figure 36: ^{13}C NMR spectrum (126 MHz, $CDCl_3$, 25°C) of the isolated polymer corresponding to table 3, run #1

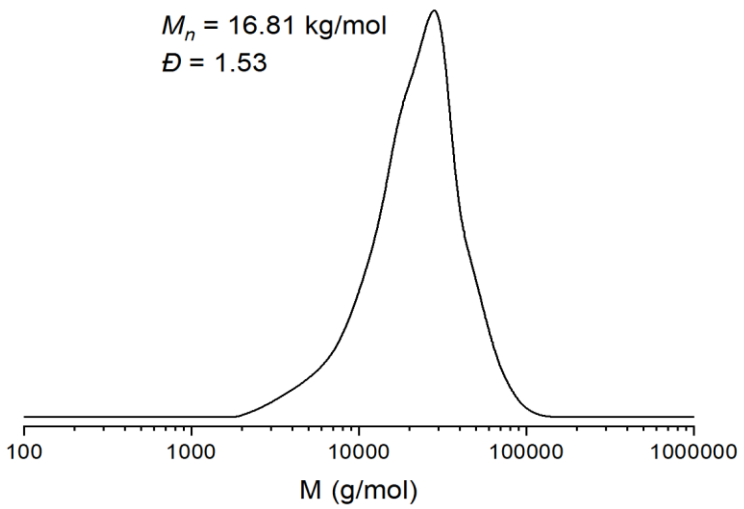

Supplementary Figure 37: GPC trace corresponding to table 3, run #1.

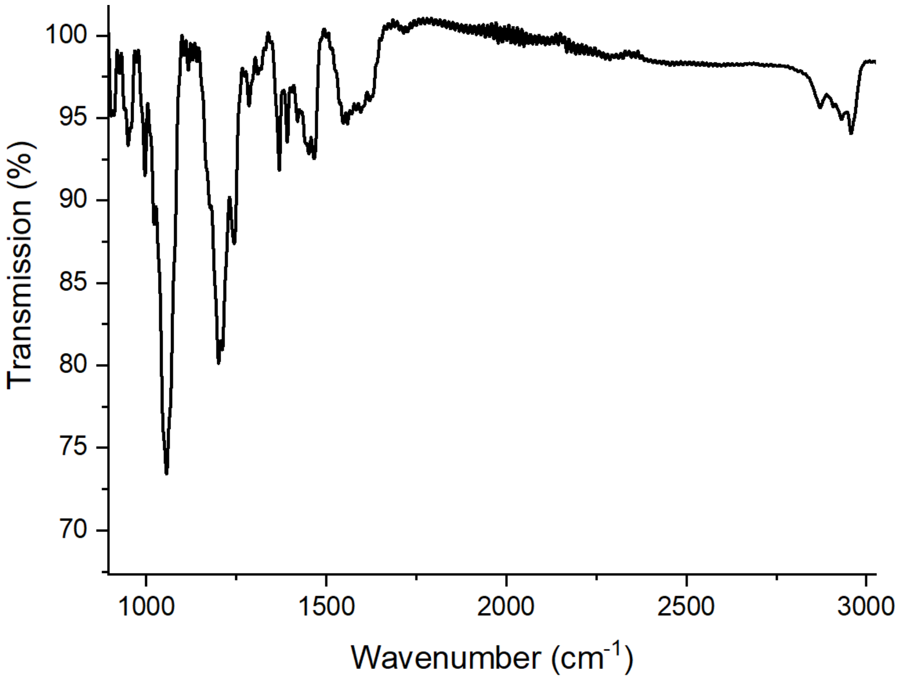

Supplementary Figure 38: IR spectrum corresponding to table 3, run #1.

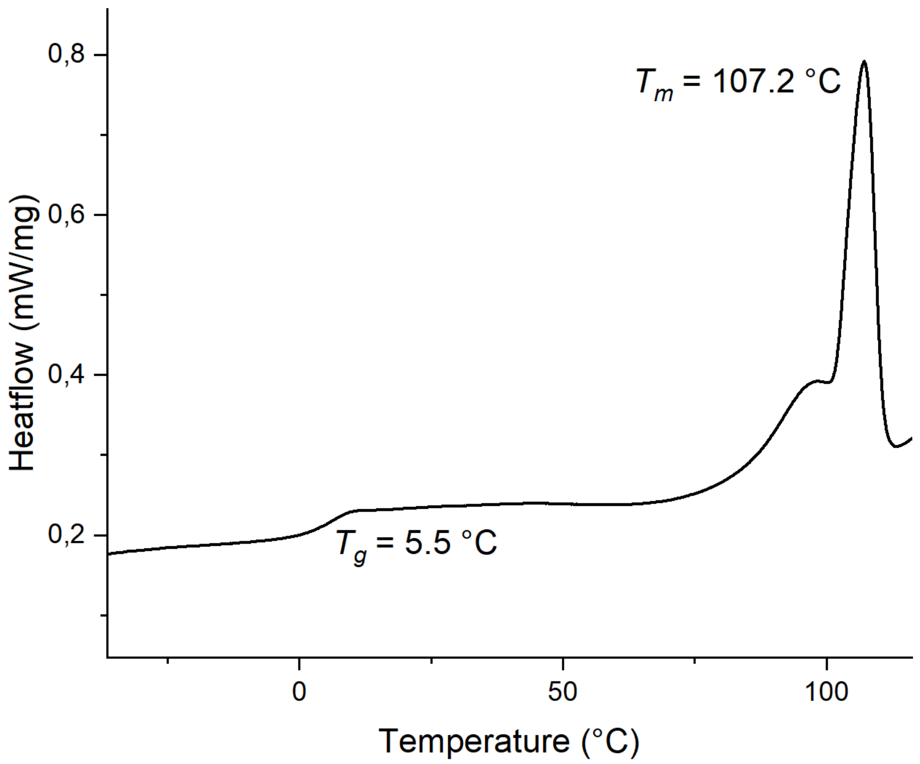

Supplementary Figure 39: DSC data from first heating cycle of copolymer corresponding to table 3, run #1.

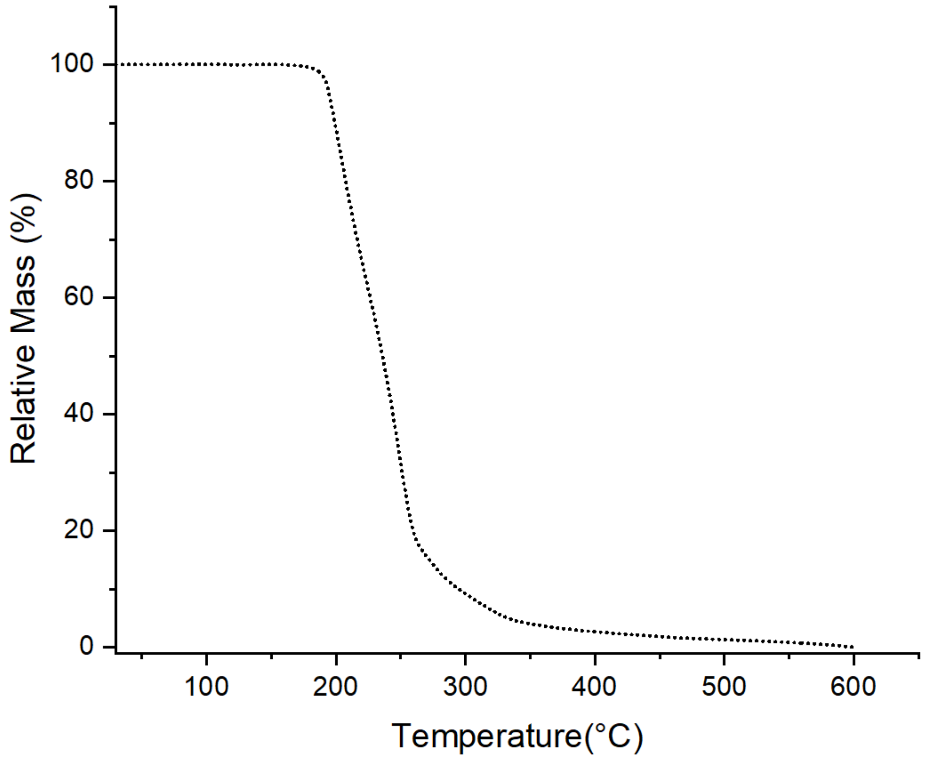

Supplementary Figure 40: TGA data corresponding to table 3, run #1. $T_{d,5\%}$ = 195.0 ºC.

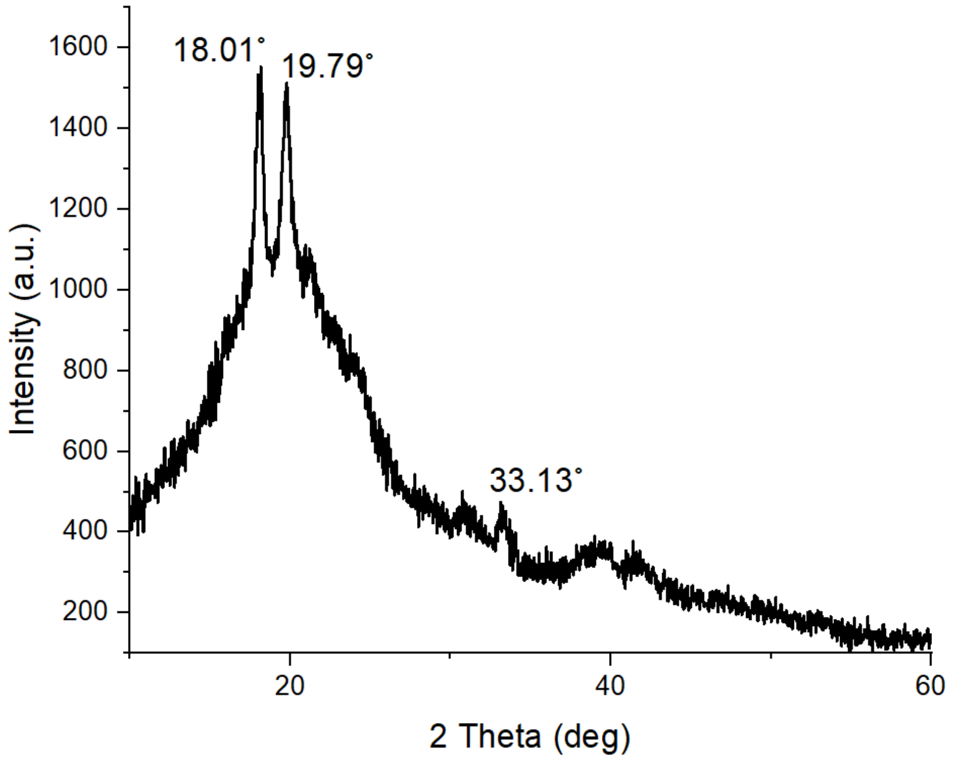

Supplementary Figure 41: PXRD data of copolymer corresponding to table 3, run #1.

Supplementary Notes 6: CS_2/OX^{OR} ROCOP with LCrK

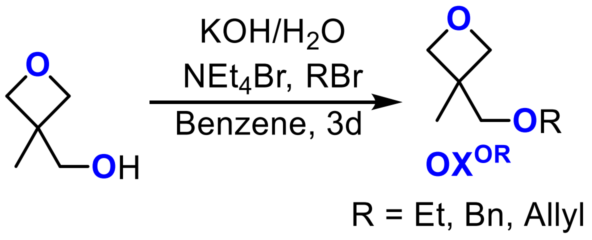

Supplementary Figure 42: Synthesis of OX^{OR}.

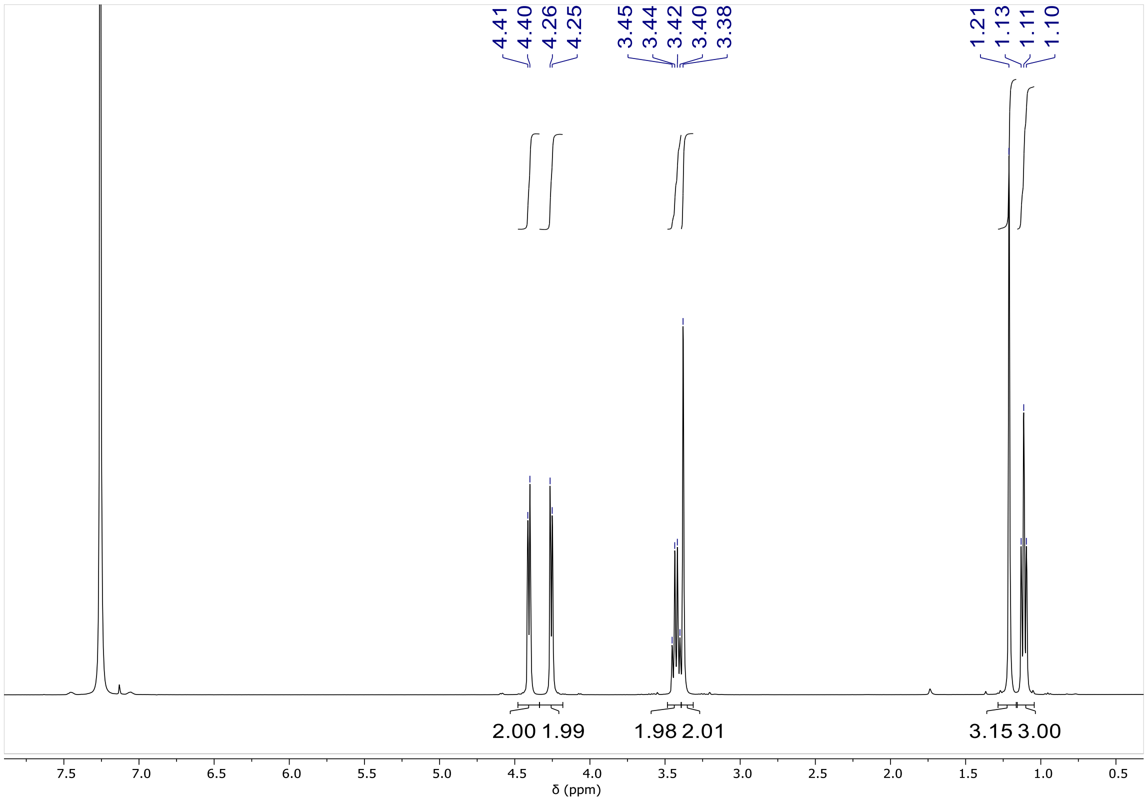

Supplementary Figure 43: ^{1}H NMR spectrum (400 MHz, $CDCl_3$, 25°C) of OX^{OEt}

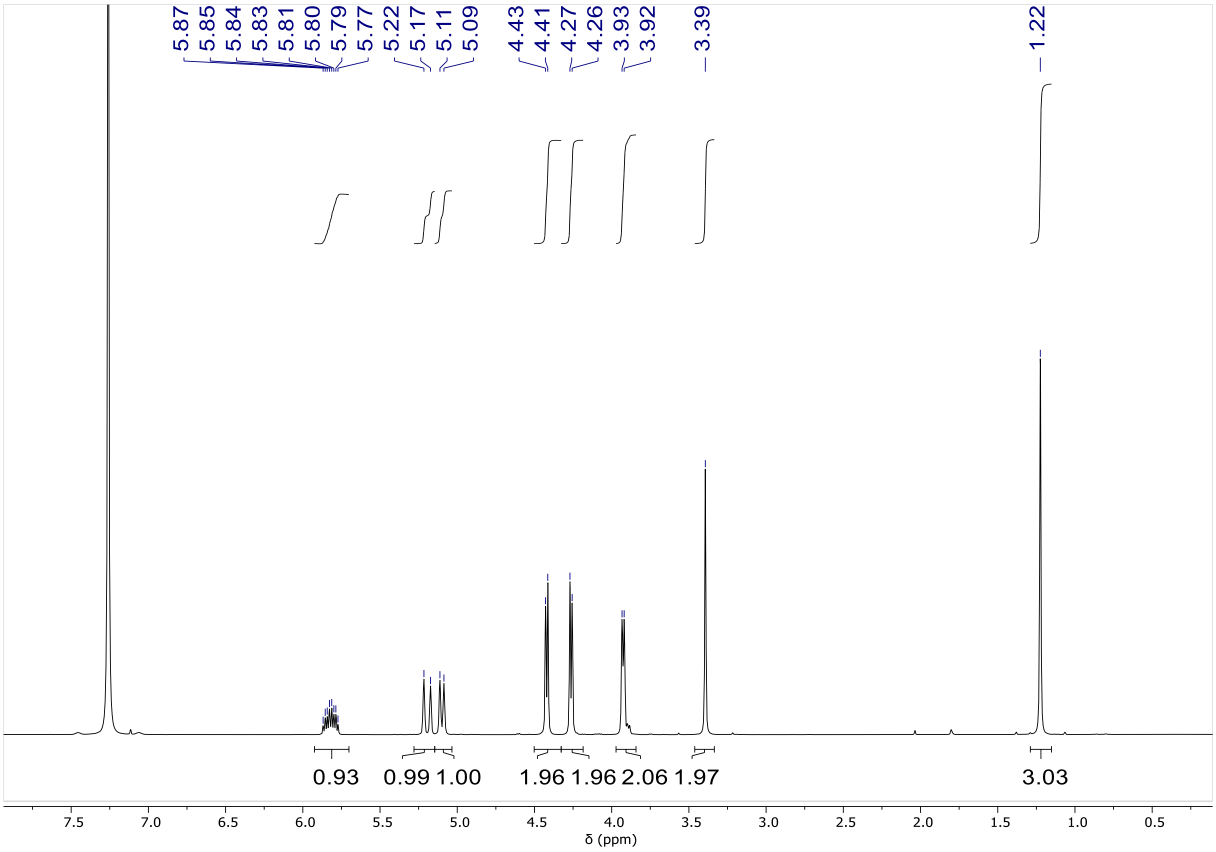

Supplementary Figure 44: ^{1}H NMR spectrum (400 MHz, $CDCl_3$, 25°C) of OX^{OAll}.

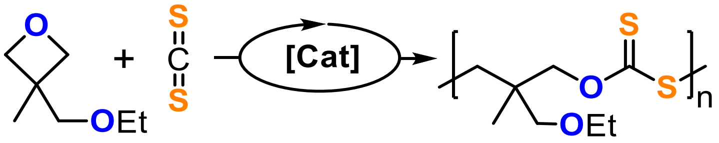

Supplementary Figure 45: Synthesis of CS_2/OX^{OEt} copolymer.

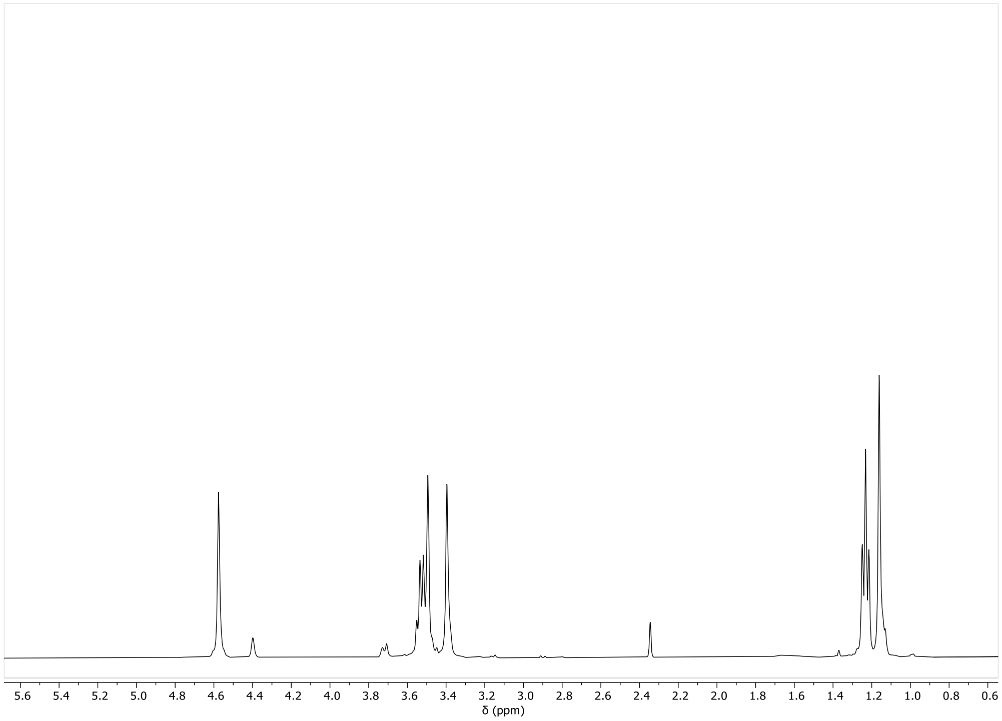

Supplementary Figure 46: ^{1}H NMR spectrum (400 MHz, $CDCl_3$, 25°C) of the crude reaction mixture showing the absence of cyclic carbonate byproducts corresponding to table 3, run #2.

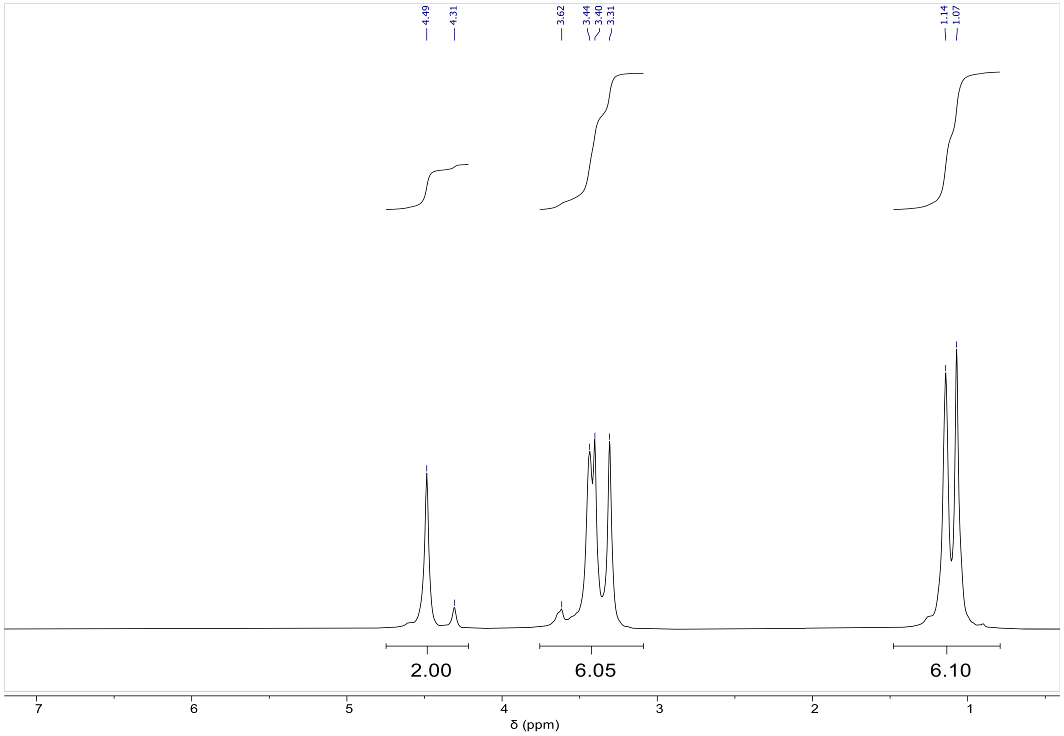

Supplementary Figure 47: ^{1}H NMR spectrum (600 MHz, $CDCl_3$, 25°C) of the isolated polymer corresponding to table 3, run #2.

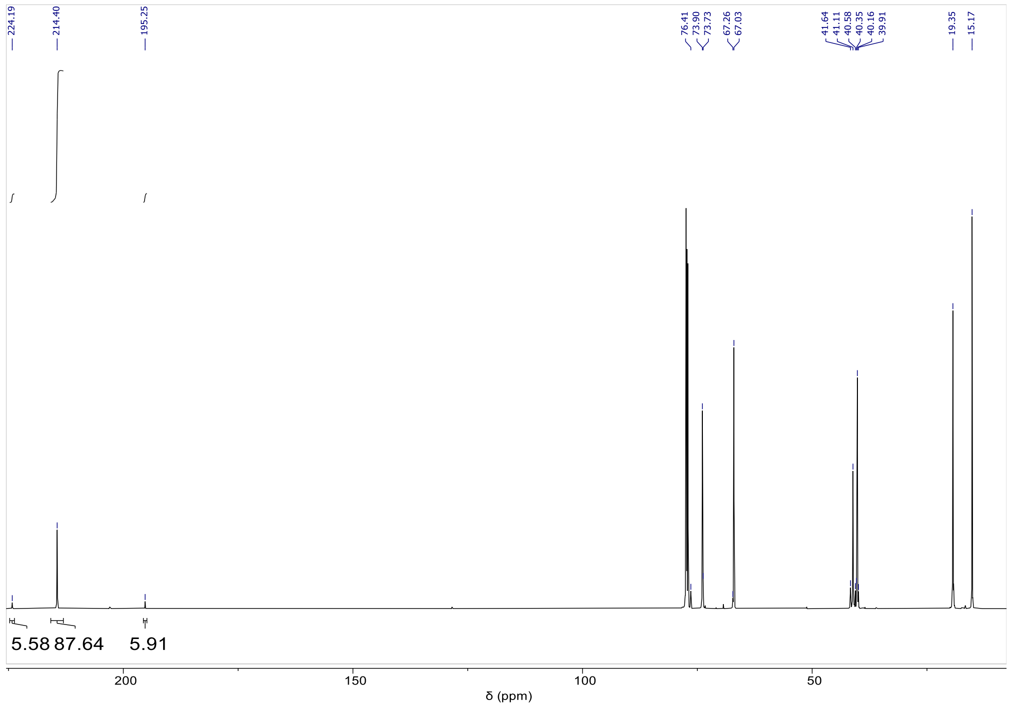

Supplementary Figure 48: $^{13}C\{^{1}H\}$ NMR spectrum (126 MHz, $CDCl_3$, 25°C) of the isolated polymer corresponding to table 3, run #2. Due to overlapping singals in the ^{1}H NMR linkage selectivity had to be approximated by the relative ^{13}C NMR integrals of the heterocarbonate resonances.[6]

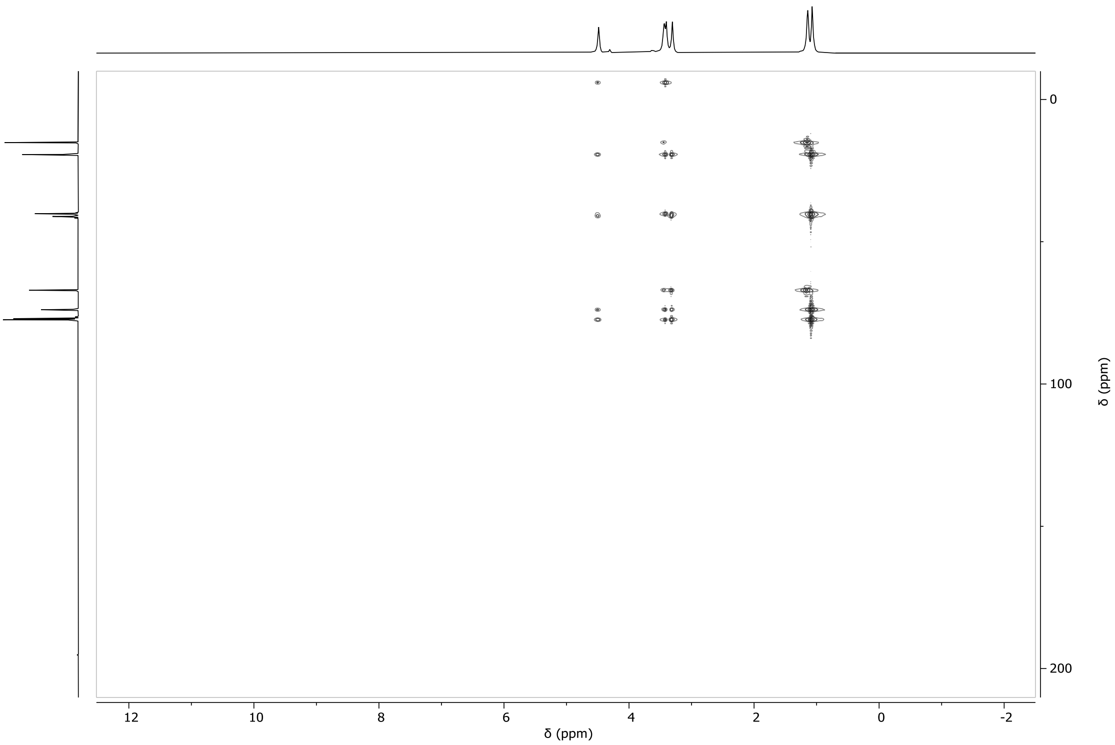

Supplementary Figure 49: ^{1}H-^{13}C HMBC NMR spectrum ($CDCl_3$, 25°C) spectrum of the isolated polymer corresponding to table 3, run #2. Signals above 210 ppm folded.

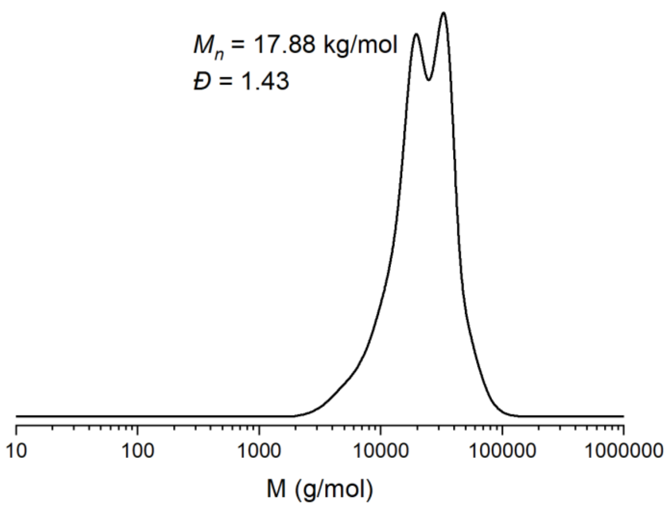

Supplementary Figure 50: GPC trace corresponding to table 3, run #2.

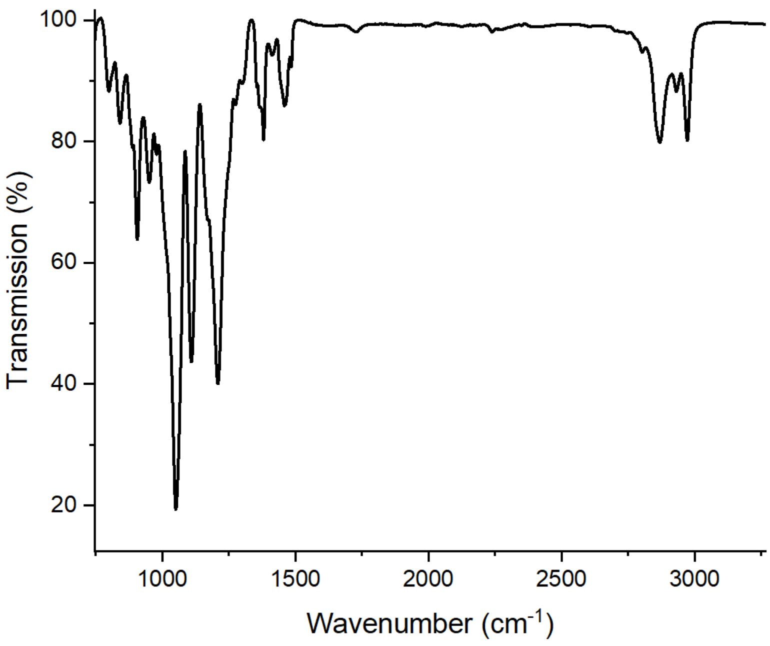

Supplementary Figure 51: IR spectrum corresponding to table 3, run #2.

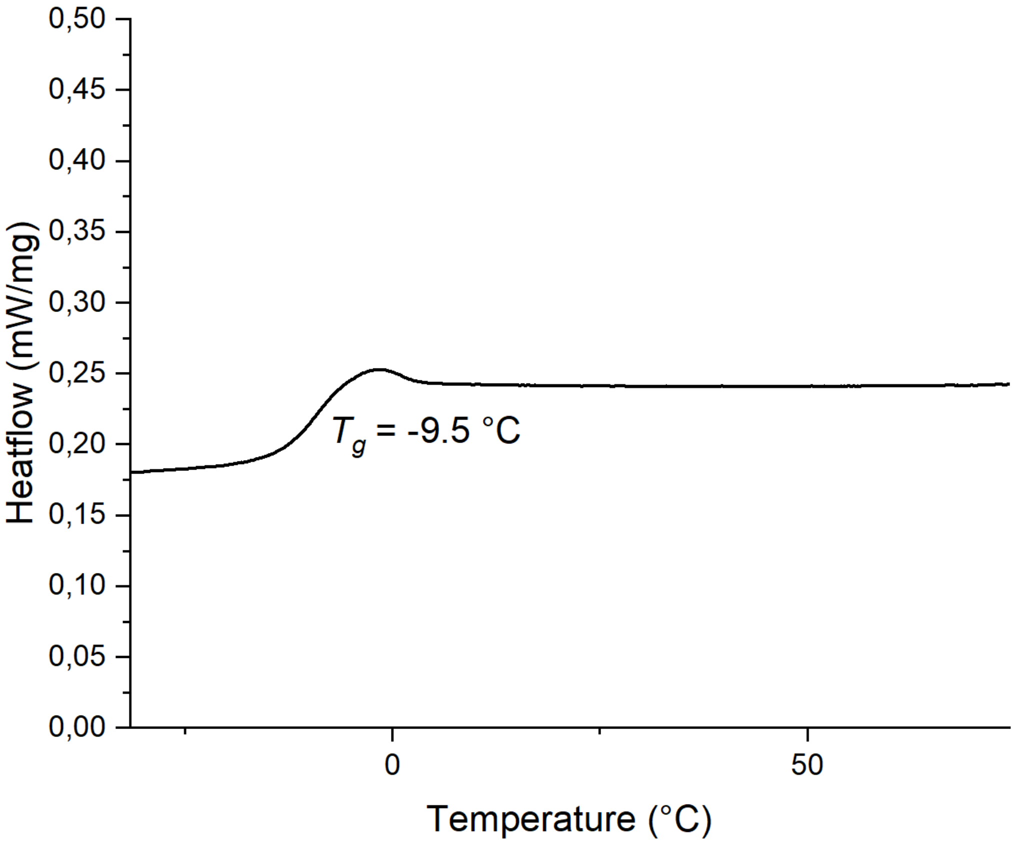

Supplementary Figure 52: DSC data from second heating cycle of copolymer corresponding to table 3, run #2.

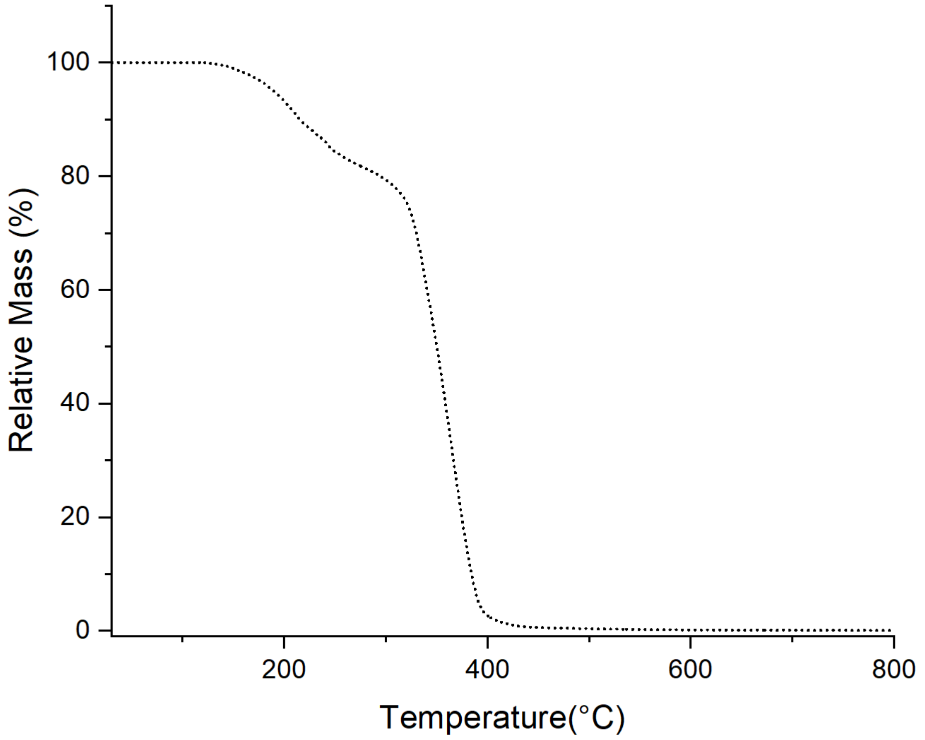

Supplementary Figure 53: TGA data corresponding to table 3, run #2. $T_{d,5\%}$ = 190.5 ºC.

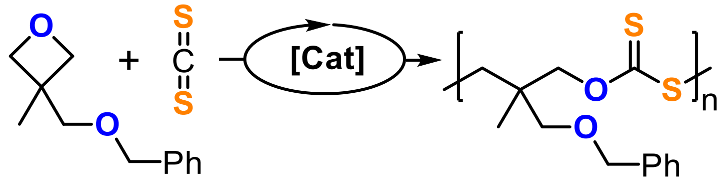

Supplementary Figure 54: Synthesis of CS_2/OX^{OBn} copolymer.

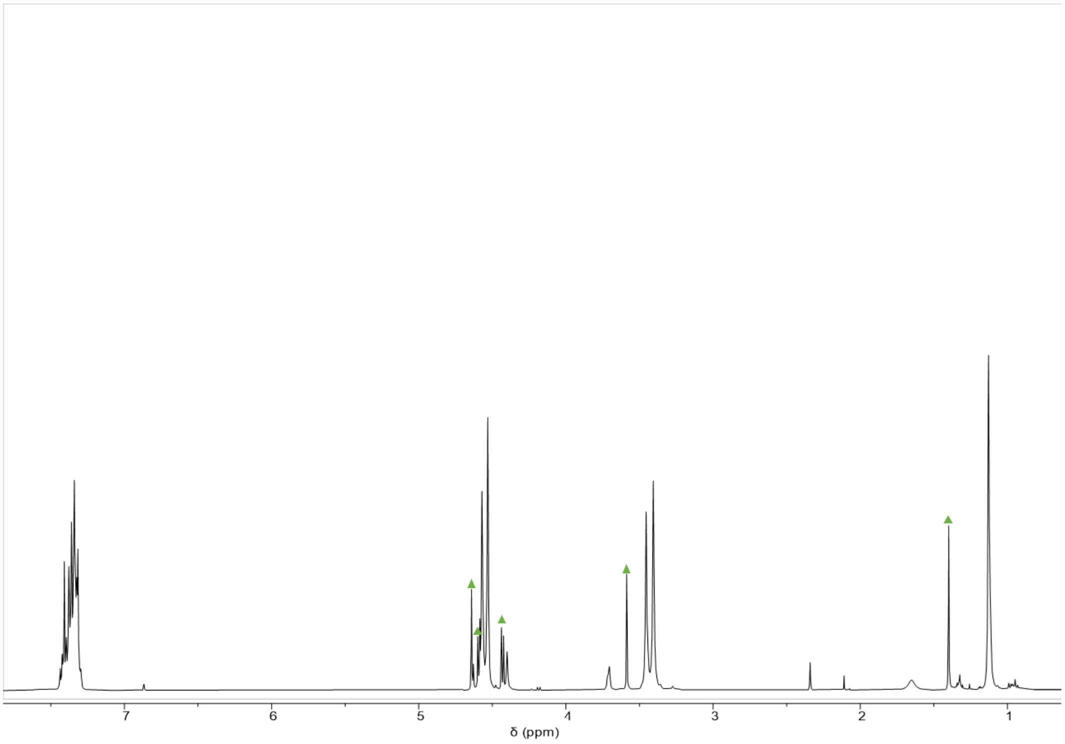

Supplementary Figure 55: ^{1}H NMR spectrum (400 MHz, $CDCl_3$, 25°C) of the crude reaction mixture showing the absence of cyclic carbonate byproducts corresponding to table 3, run #3. Peaks labelled with green triangle which are not part of the polymer (*vide infra*) correspond to residual unconsumed OX^{OBn}.

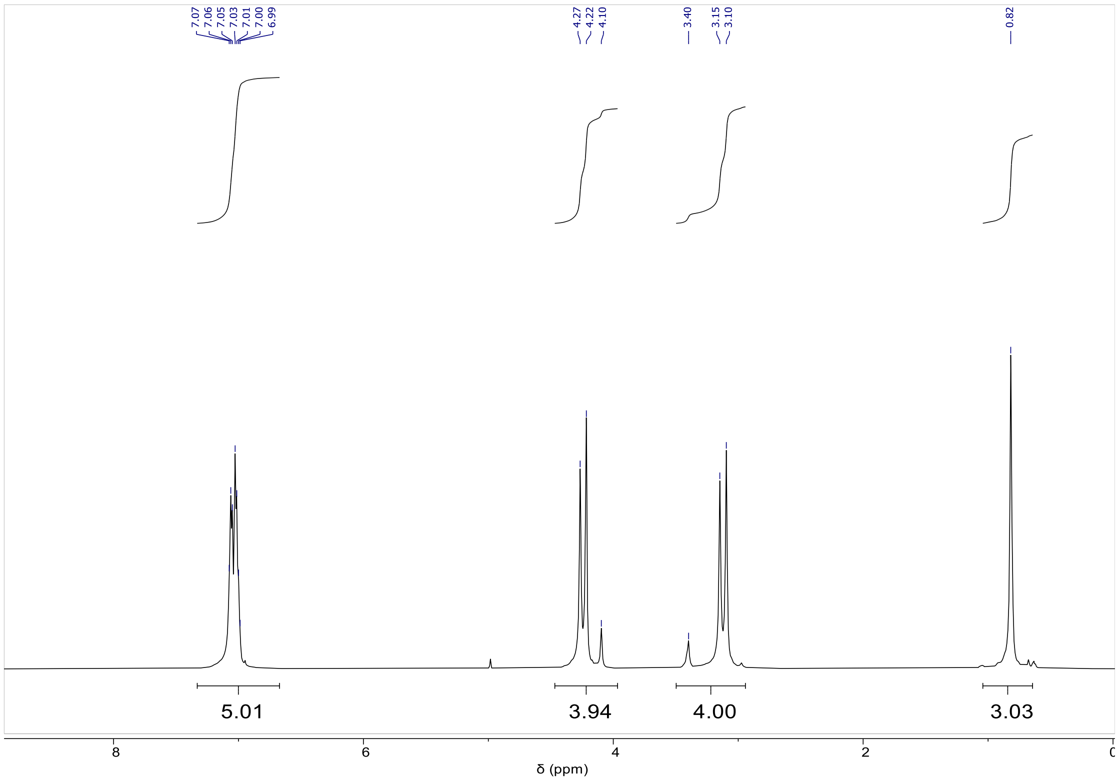

Supplementary Figure 56: ^{1}H NMR spectrum (400 MHz, $CDCl_3$, 25°C) of the isolated polymer corresponding to table 3, run #3.

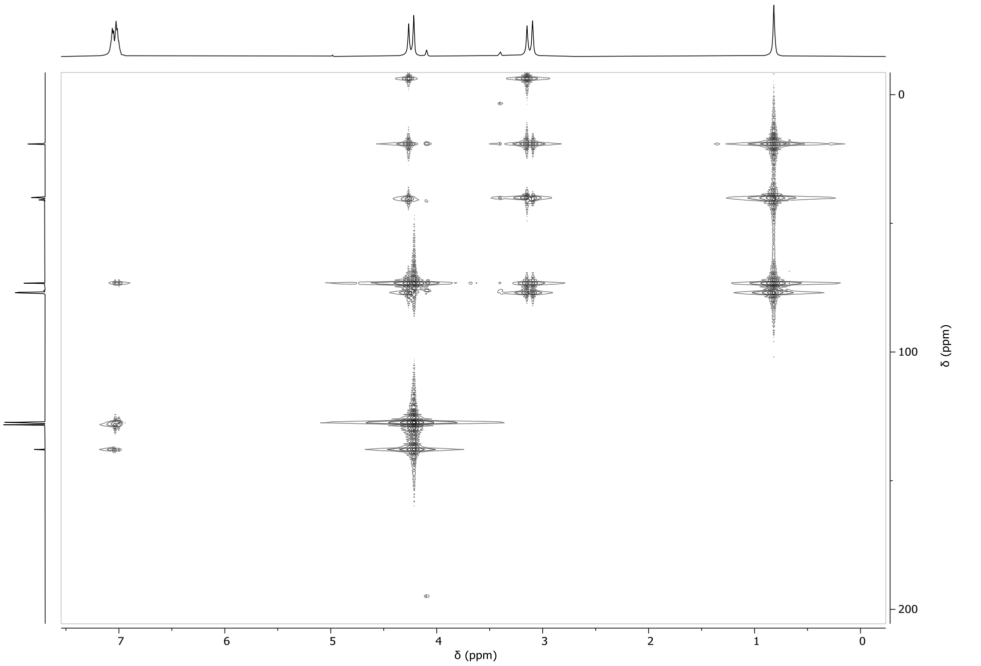

Supplementary Figure 57: ^{1}H-^{13}C HMBC NMR spectrum ($CDCl_3$, 25°C) spectrum of the isolated polymer corresponding to table 3, run #3. Signals above 210 ppm folded.

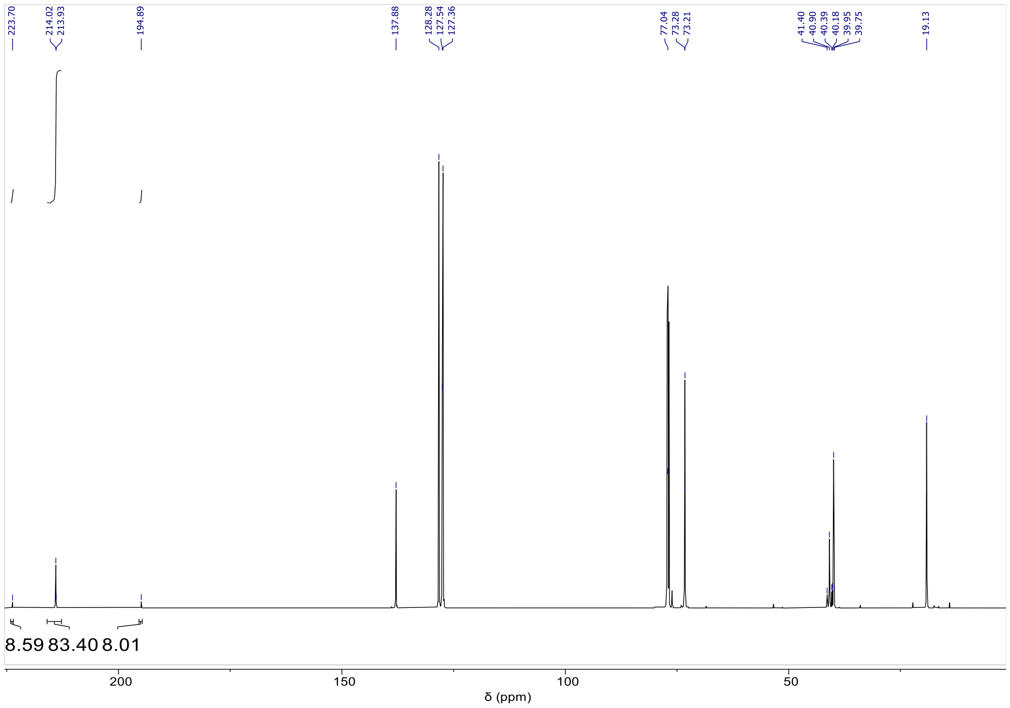

Supplementary Figure 58: $^{13}C\{^1H\}$ NMR spectrum (126 MHz, $CDCl_3$, 25°C) of the isolated polymer corresponding to table 3, run #3. Due to overlapping singals in the ^1H NMR linkage selectivity had to be approximated by the relative ^{13}C NMR integrals of the heterocarbonate resonances.[6]

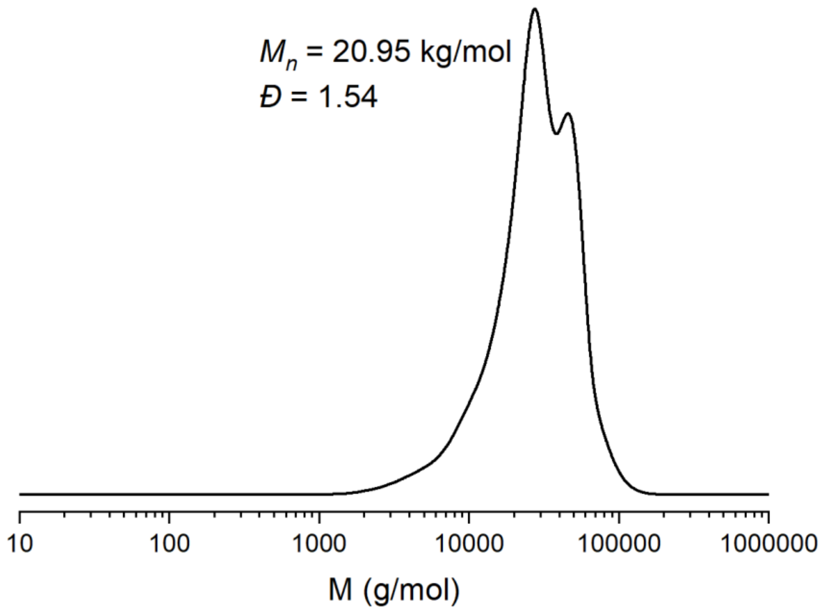

Supplementary Figure 59: GPC trace corresponding to table 3, run #3.

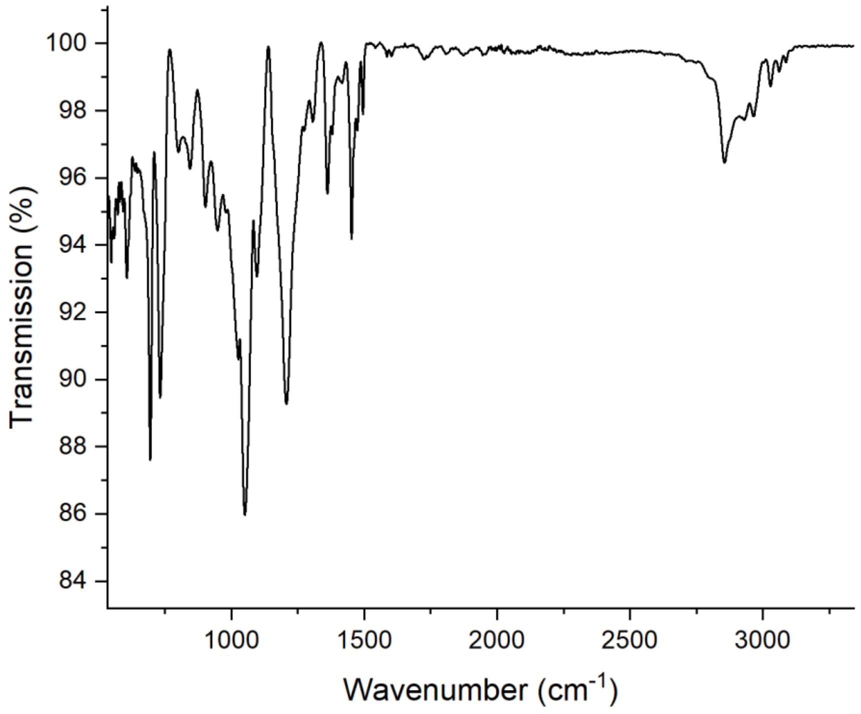

Supplementary Figure 60: IR spectrum of CS_2/OX^{OBn} copolymer.

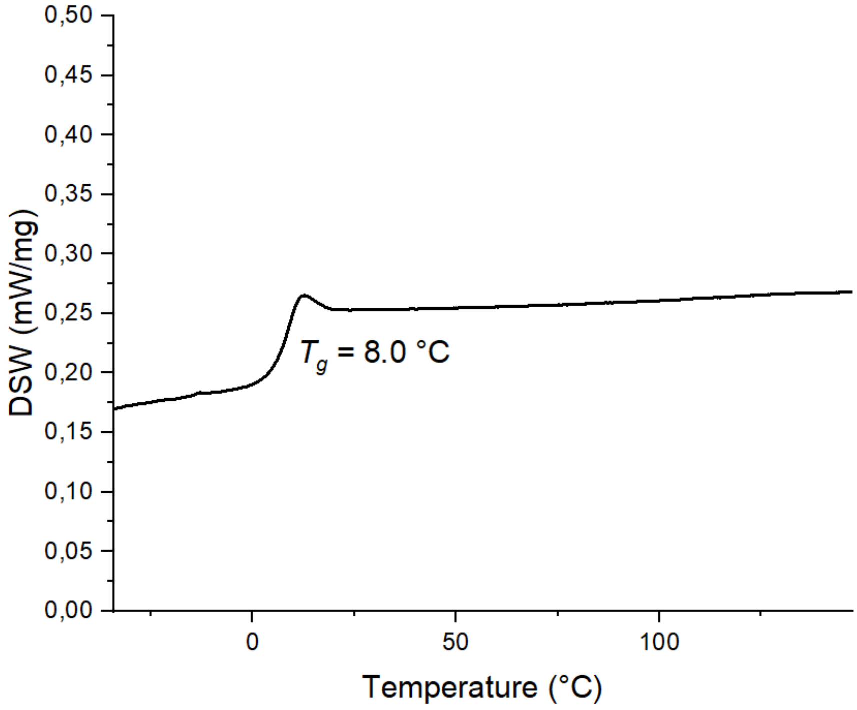

Supplementary Figure 61: DSC data from second heating cycle of copolymer corresponding to table 3, run #3.

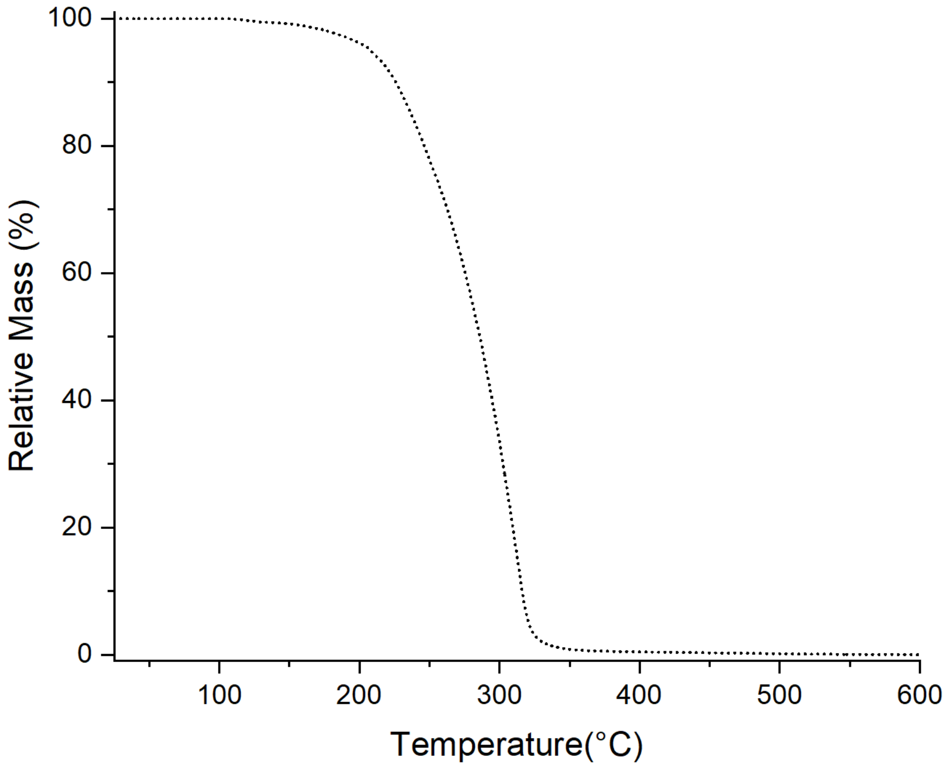

Supplementary Figure 62: TGA data of CS_2/OX^{OBn} copolymer. $T_{d,5\%}$ = 208.0 ºC.

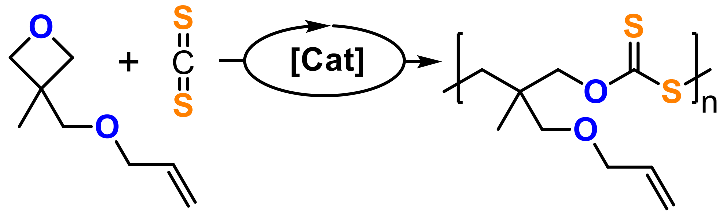

Supplementary Figure 63: Synthesis of CS_2/OX^{OAll} copolymer.

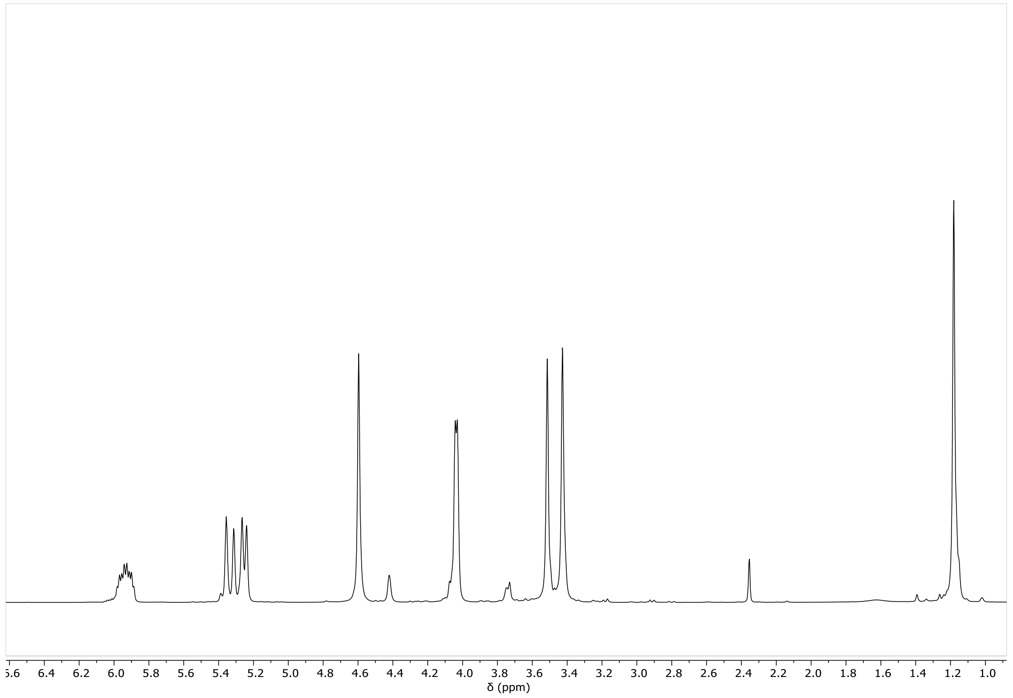

Supplementary Figure 64: ^{1}H NMR spectrum (400 MHz, $CDCl_3$, 25°C) of the crude reaction mixture showing the absence of cyclic carbonate byproducts corresponding to table 3, run #4.

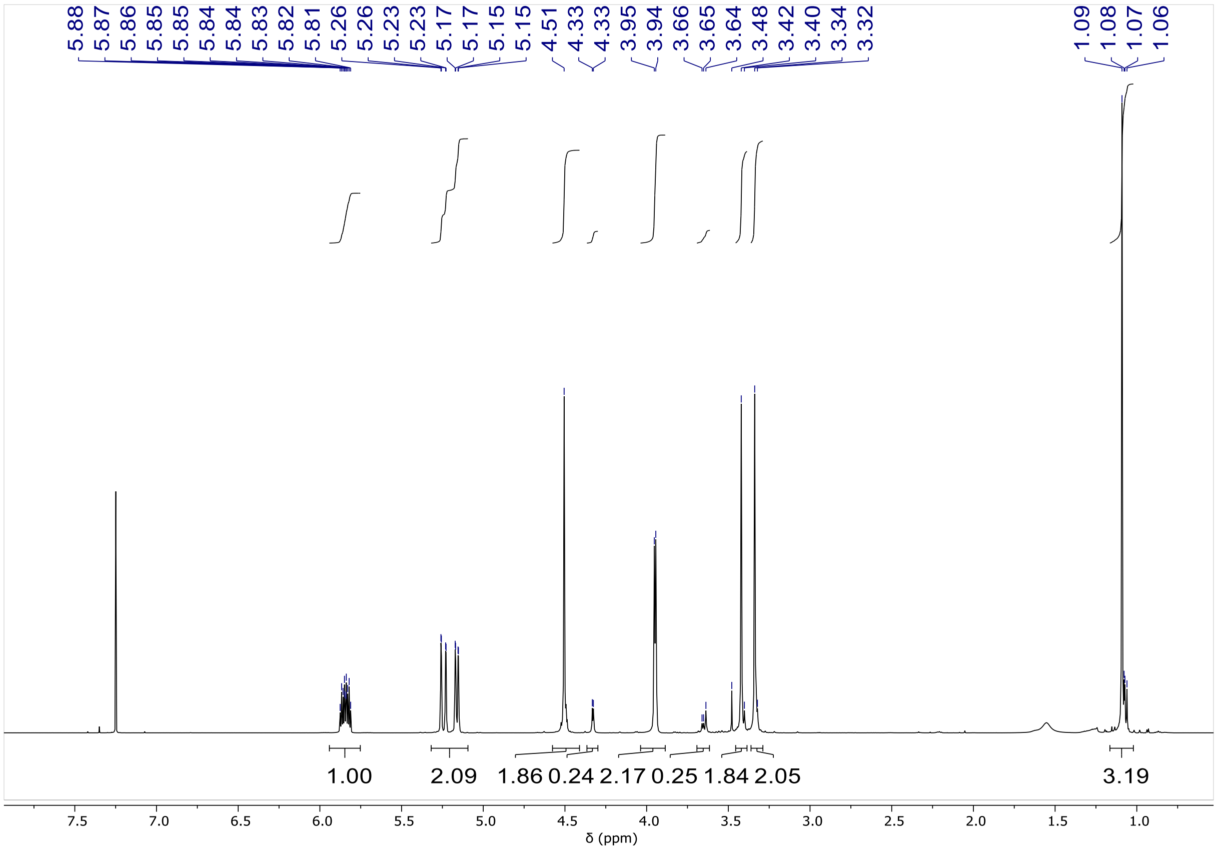

Supplementary Figure 65: ^{1}H NMR spectrum (600 MHz, $CDCl_3$, 25°C) of the isolated polymer corresponding to table 3, run #4.

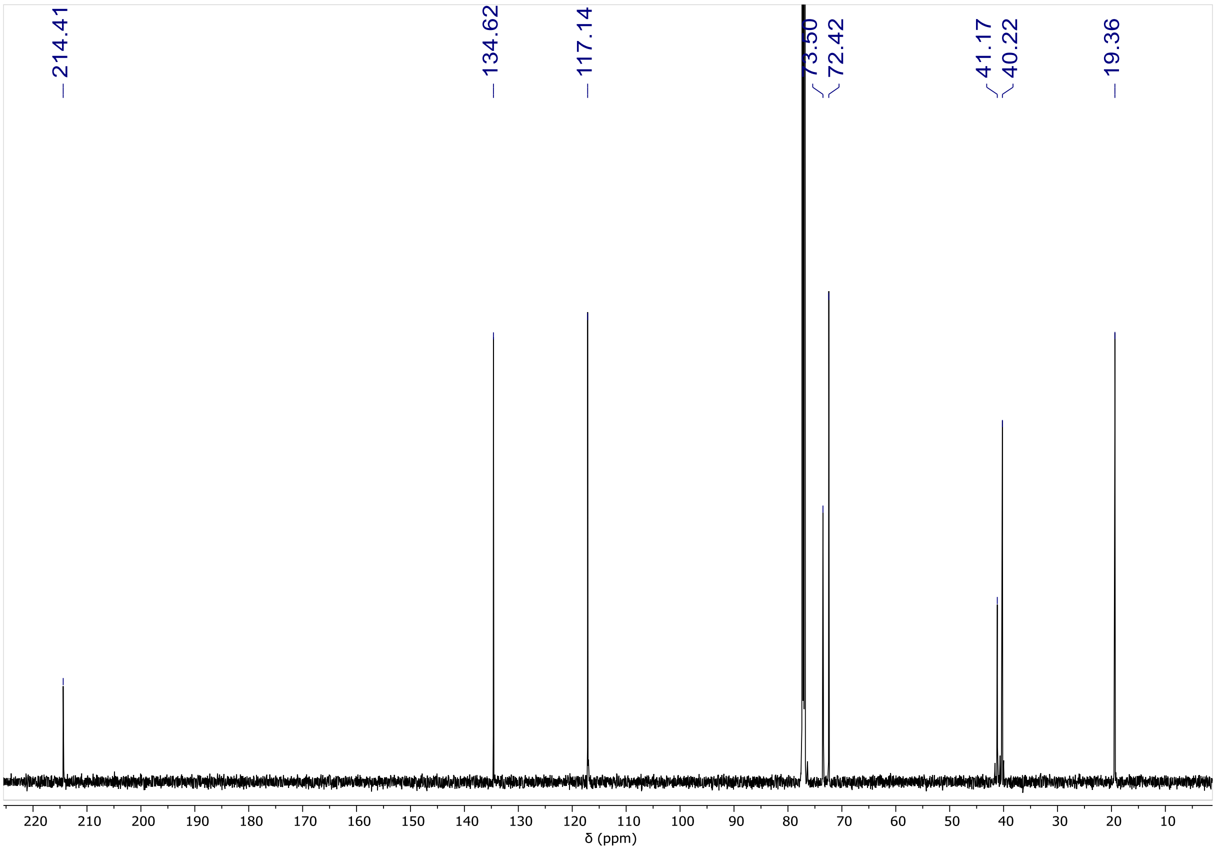

Supplementary Figure 66: $^{13}C\{^{1}H\}$ NMR spectrum (126 MHz, $CDCl_3$, 25°C) of the isolated polymer corresponding to table 3, run #4.

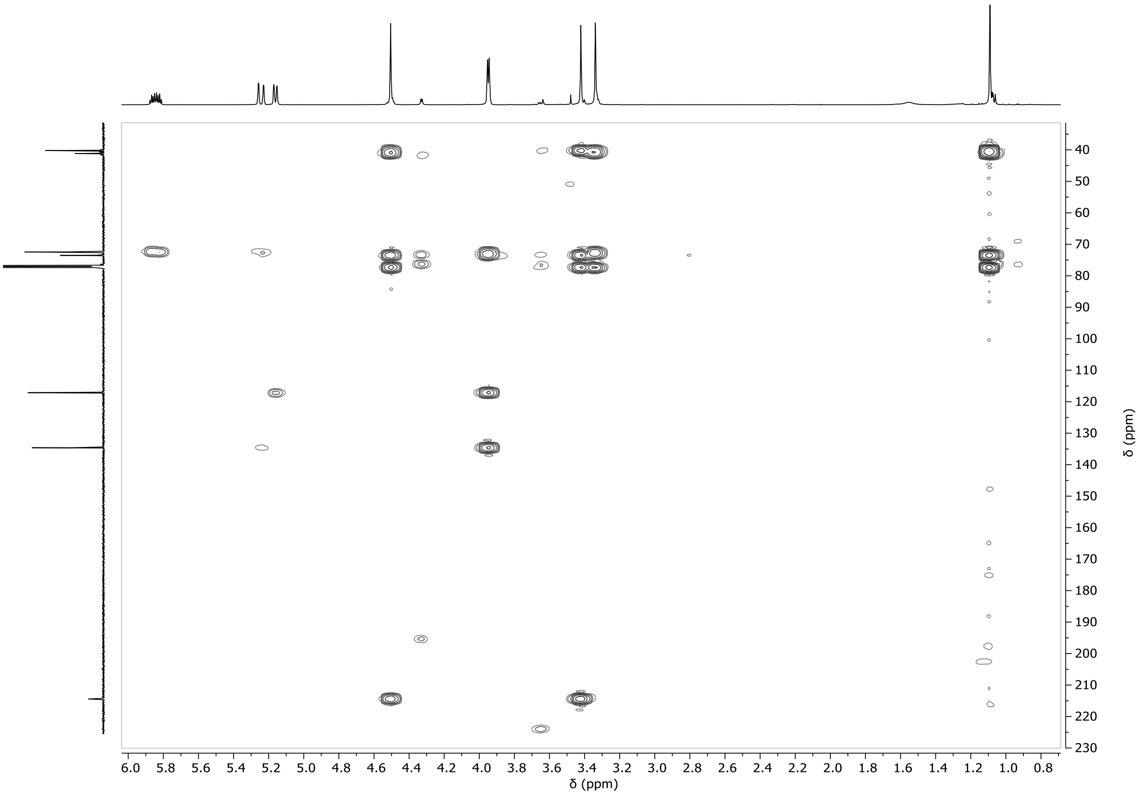

Supplementary Figure 67: ^{1}H-^{13}C HMBC NMR spectrum ($CDCl_3$, 25°C) spectrum of the isolated polymer corresponding to table 3, run #4. Signals above 210 ppm folded.

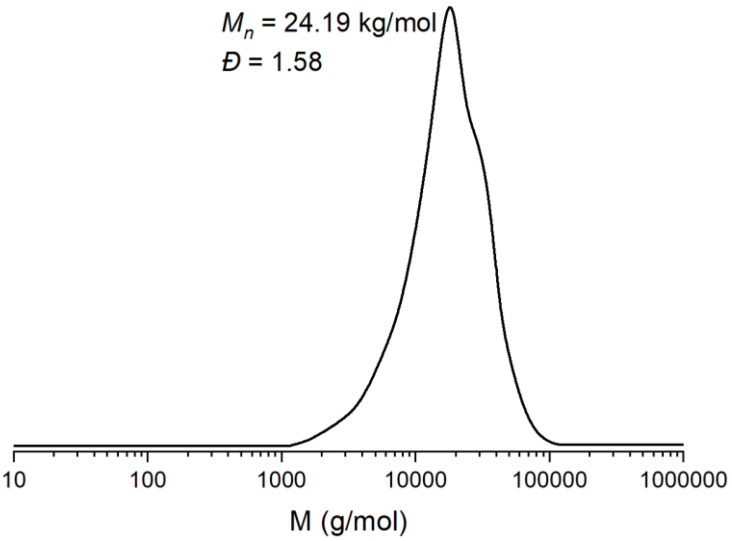

Supplementary Figure 68: GPC trace corresponding to table 3, run #4.

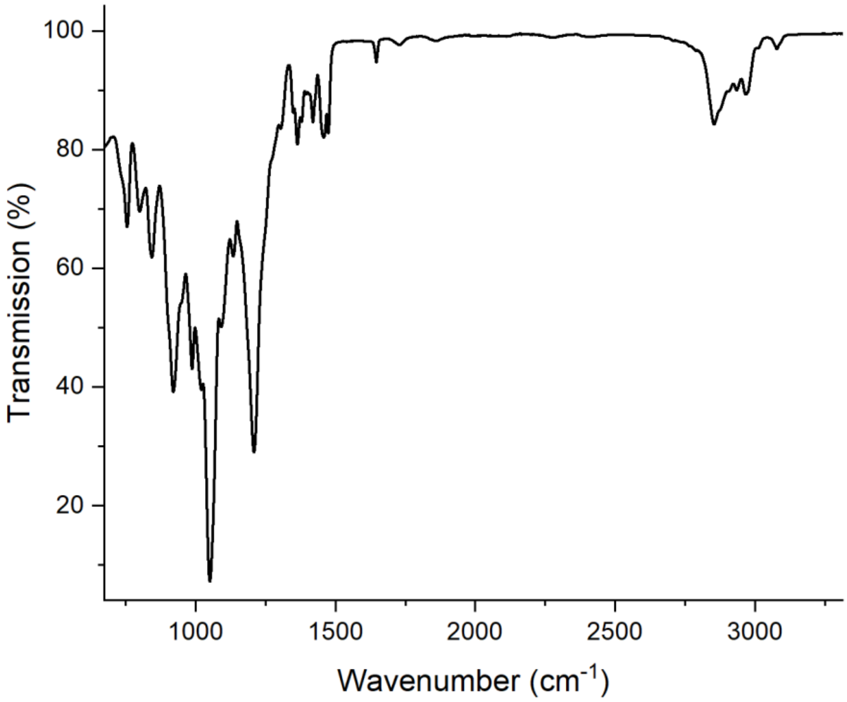

Supplementary Figure 69: IR spectrum corresponding to table 3, run #4.

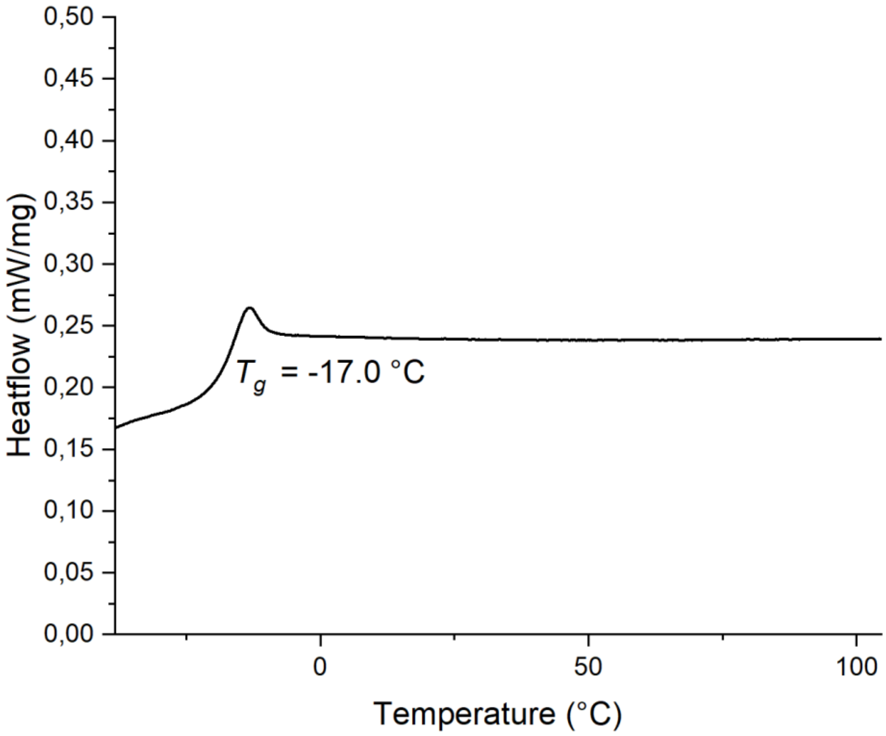

Supplementary Figure 70: DSC data from second heating cycle of copolymer corresponding to table 3, run #4.

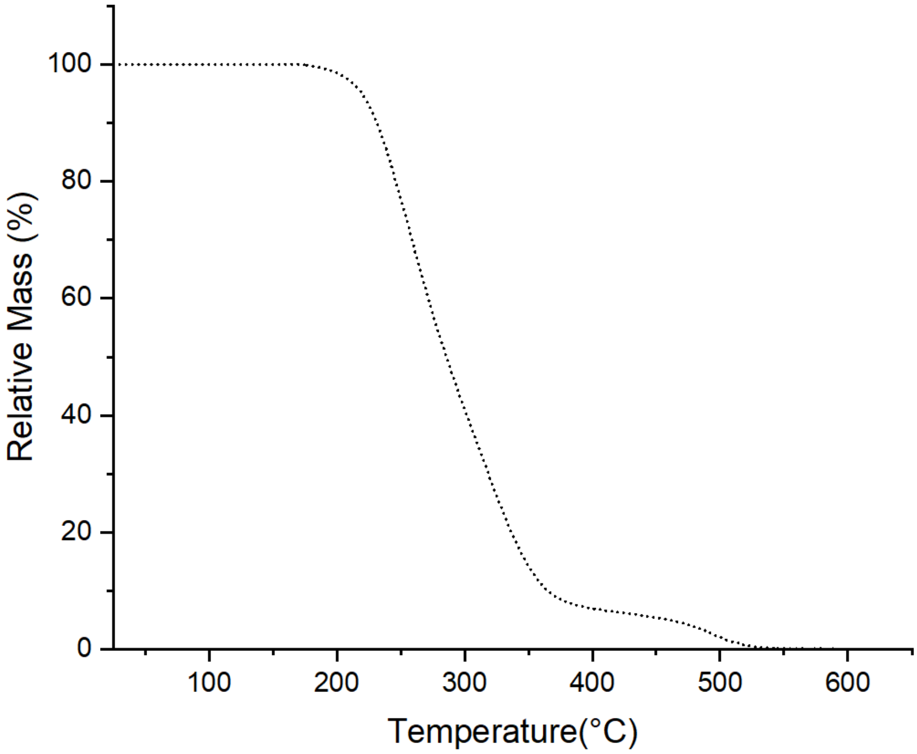

Supplementary Figure 71: TGA data corresponding to table 3, run #4. $T_{d,5\%}$ = 220.0 ºC.

Supplementary Notes 7: CS_2 ROCOP of epoxides with LCrK

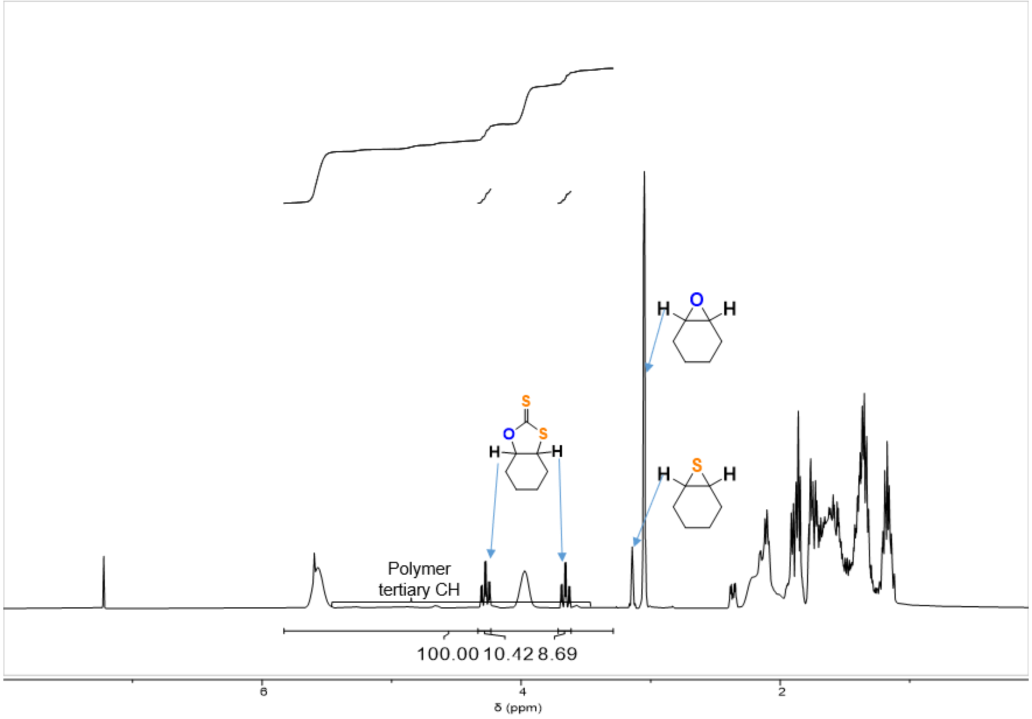

Supplementary Figure 72: ^{1}H NMR spectrum (400 MHz, $CDCl_3$, 25°C) of the crude reaction mixture showing the formation of 19% cyclic carbonate by-products corresponding to table 3, run #5.

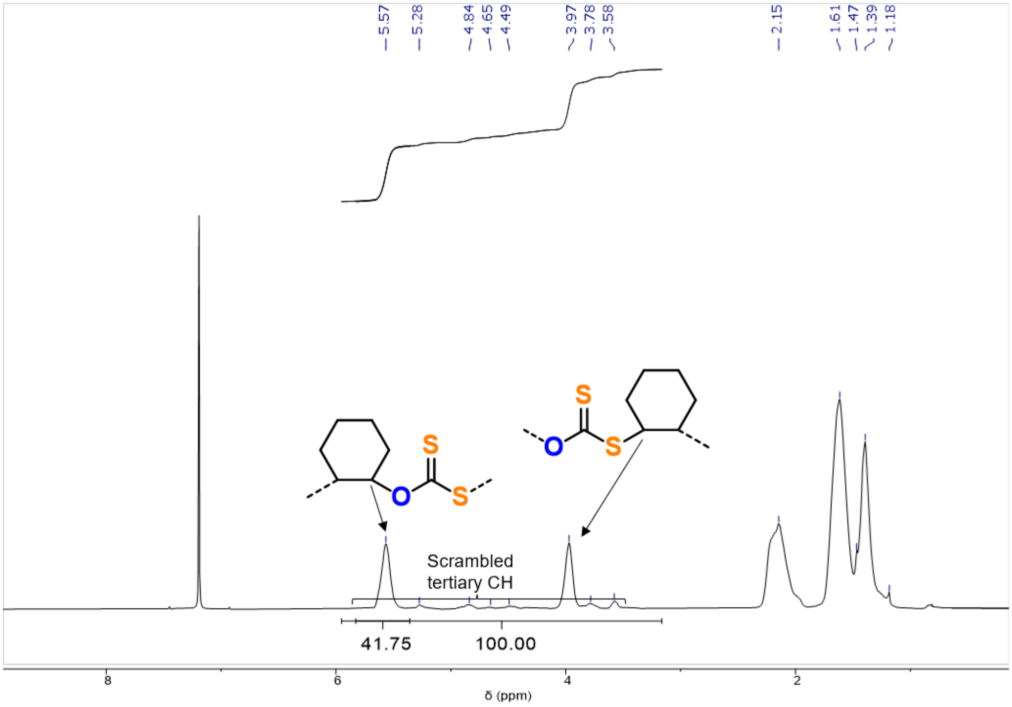

Supplementary Figure 73: ^{1}H NMR spectrum (600 MHz, $CDCl_3$, 25°C) of the isolated polymer corresponding to table 3, run #5.

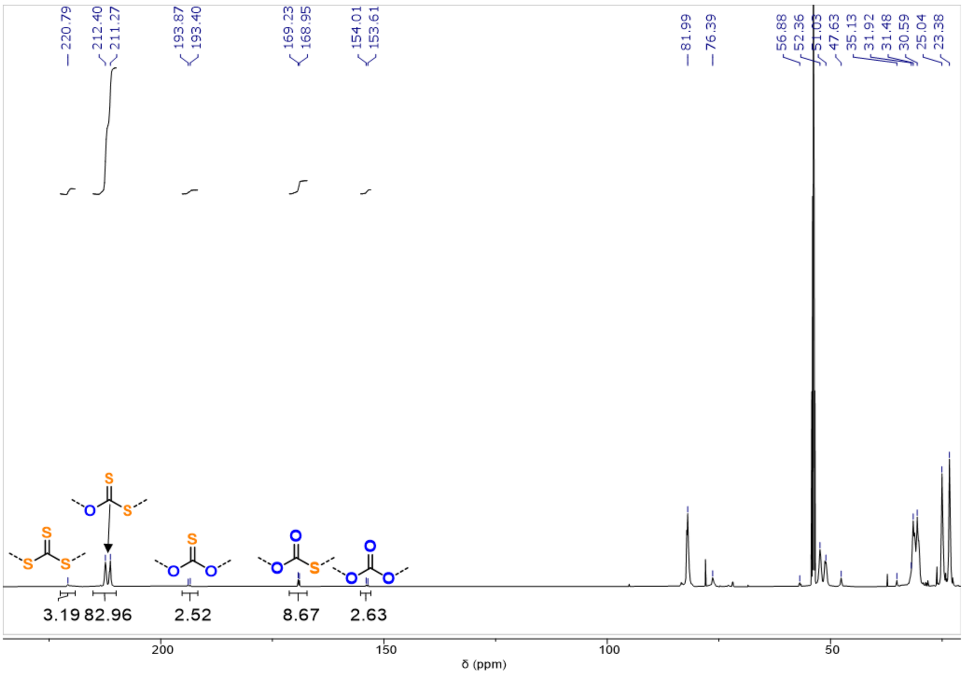

Supplementary Figure 74: $^{13}C\{^{1}H\}$ NMR spectrum (126 MHz, $CDCl_3$, 25°C) of the isolated polymer corresponding to table 3, run #5.

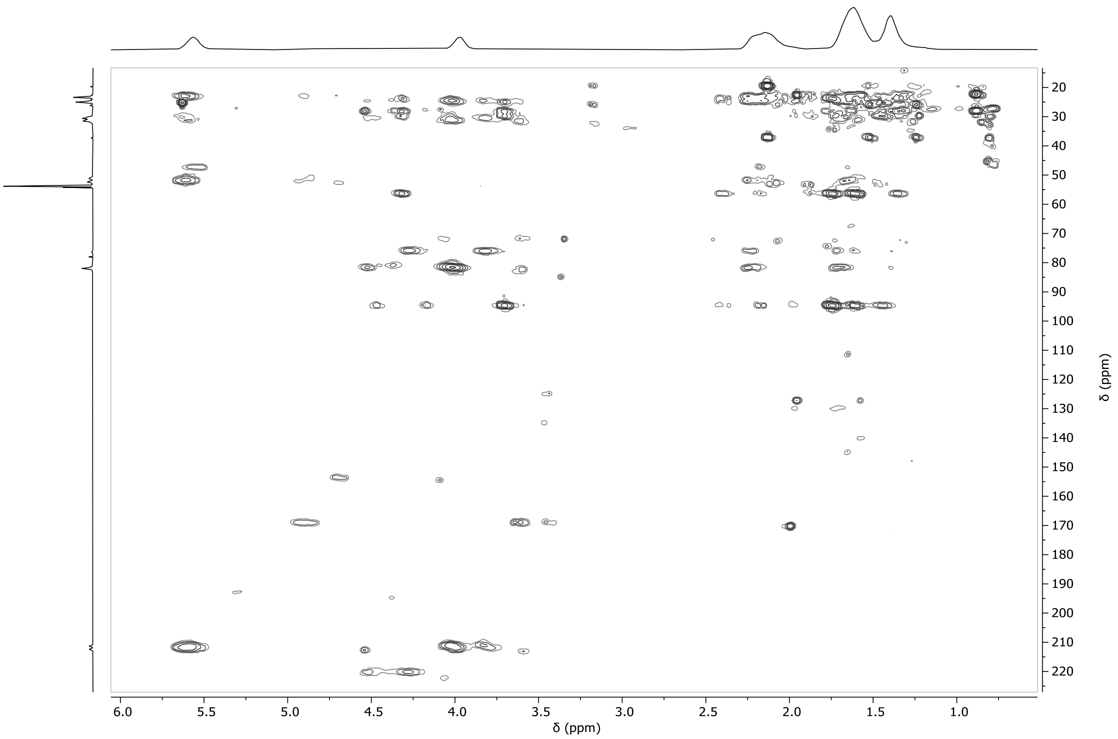

Supplementary Figure 75: ^{1}H-^{13}C HMBC NMR spectrum ($CDCl_3$, 25°C) spectrum of the isolated polymer corresponding to table 3, run #5.

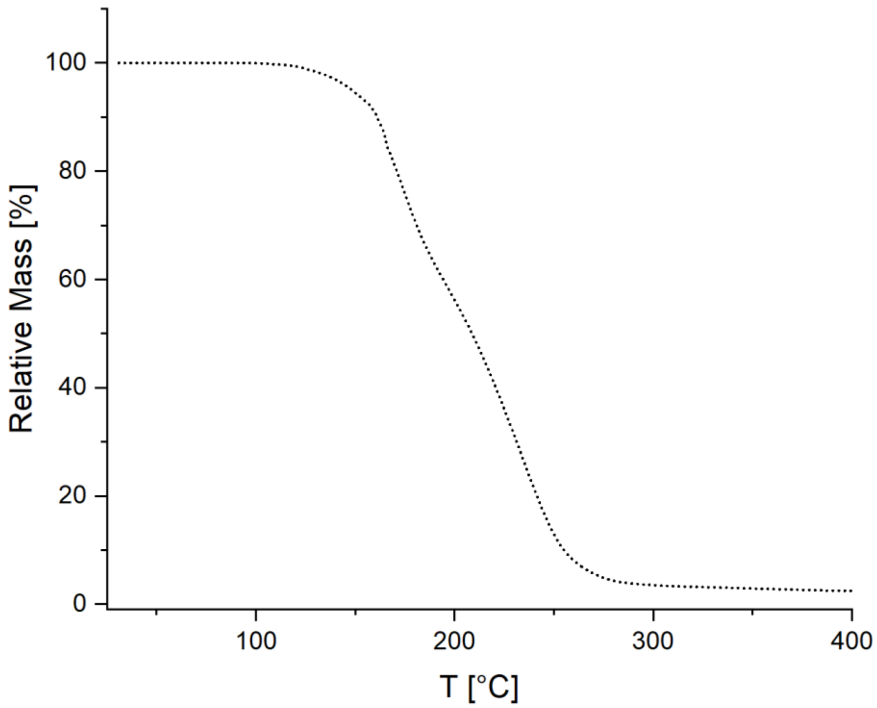

Supplementary Figure 76: TGA data corresponding to table 3, run #5. $T_{d,5\%}$ = 148.3 ºC.

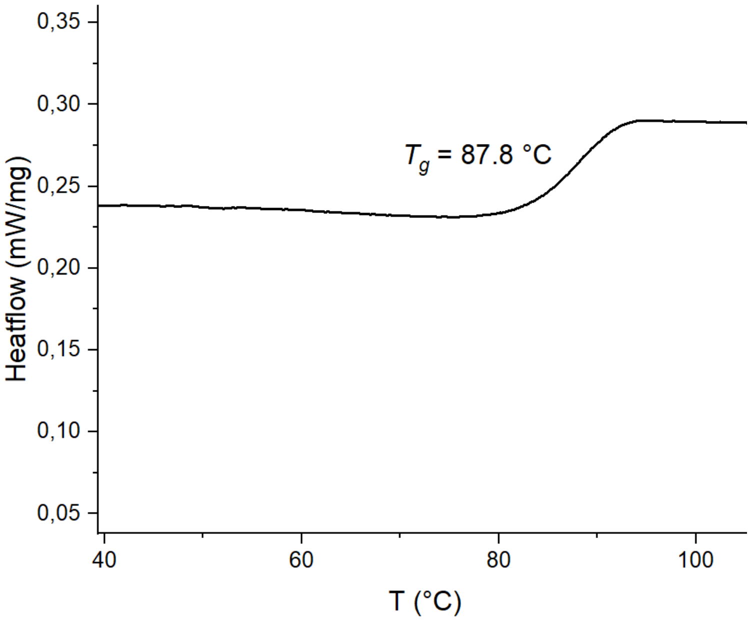

Supplementary Figure 77: DSC data from second heating cycle corresponding to table 3, run #5.

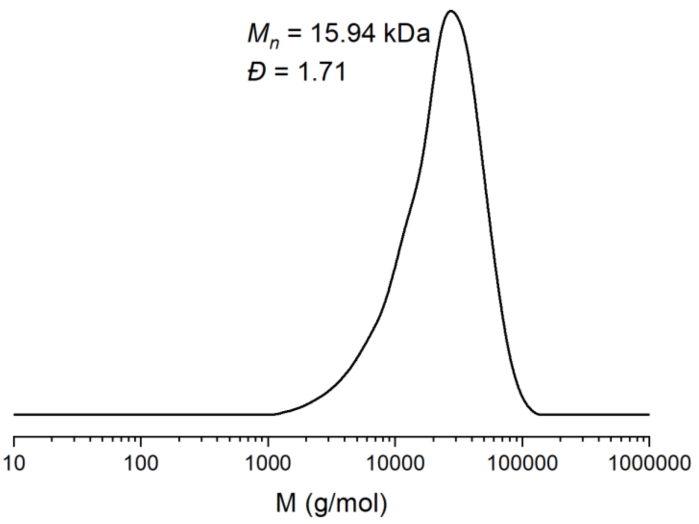

Supplementary Figure 78: GPC trace corresponding to table 3, run #5.

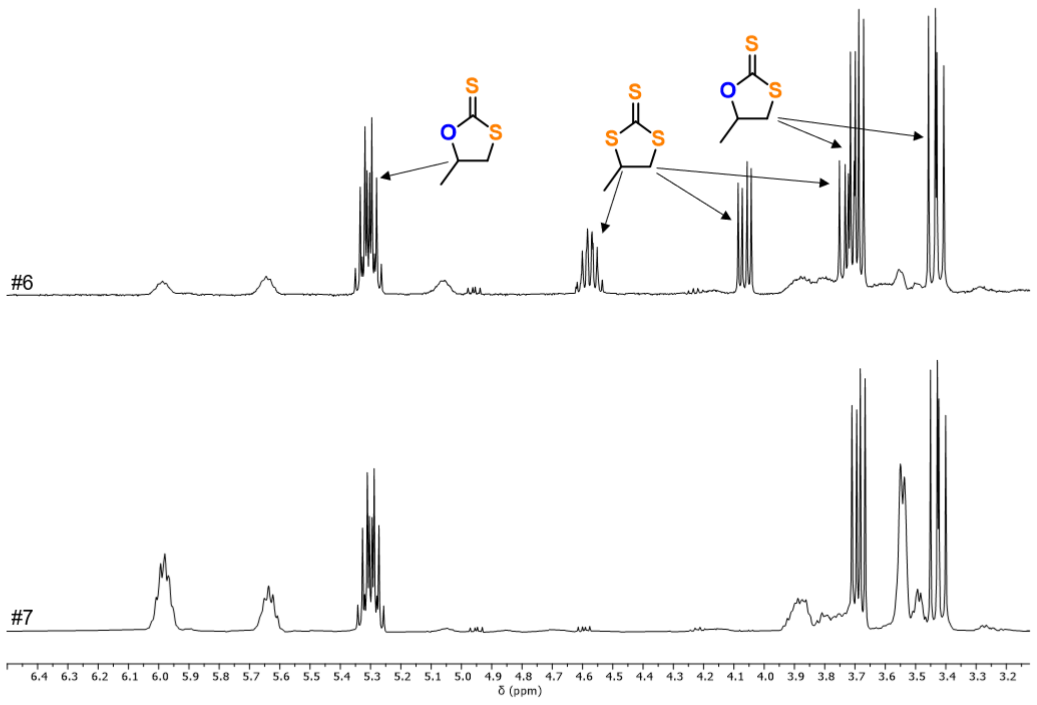

Supplementary Figure 79: Overlaid ^{1}H NMR spectra (400 MHz, $CDCl_3$, 25°C) of the crude reaction mixtures corresponding to table 3, run #6 and #7 showing less cyclic carbonate by-products going from 80ºC (#6) to 50ºC (#7).

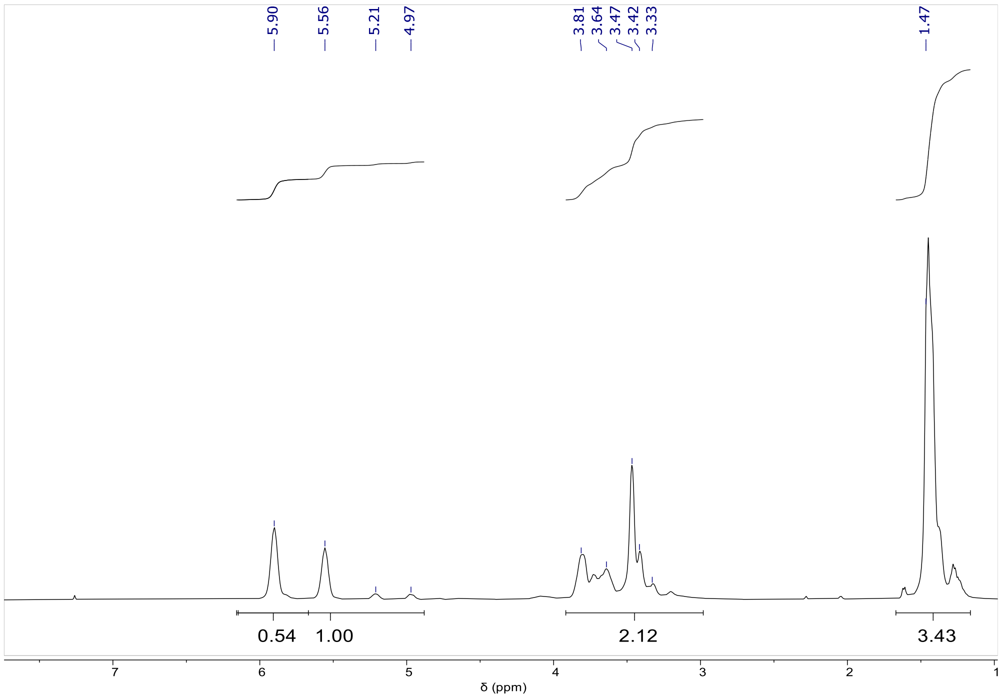

Supplementary Figure 80: ^{1}H NMR spectrum (400 MHz, $CDCl_3$, 25°C) of the isolated polymer corresponding to table 3, run #7.

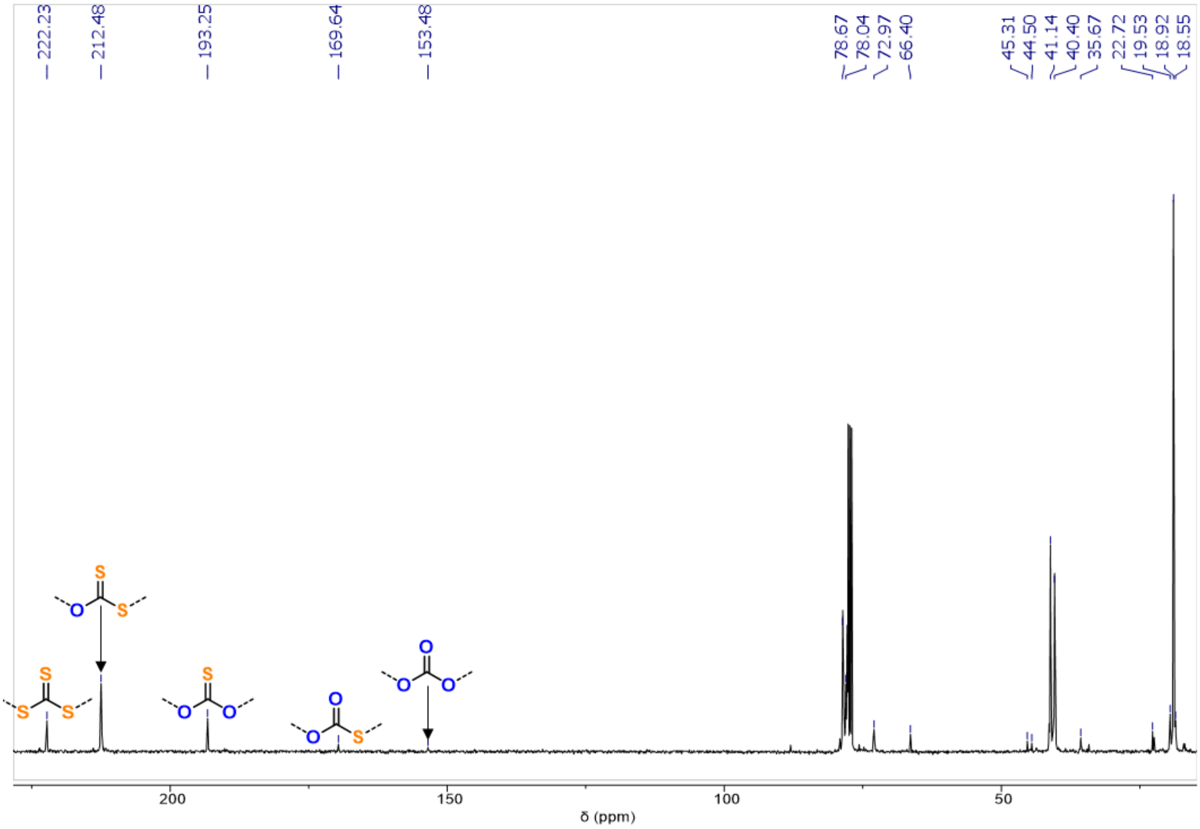

Supplementary Figure 81: ^{13}C{^{1}H} NMR spectrum (126 MHz, $CDCl_3$, 25°C) of the isolated polymer corresponding to table 3, run #7.

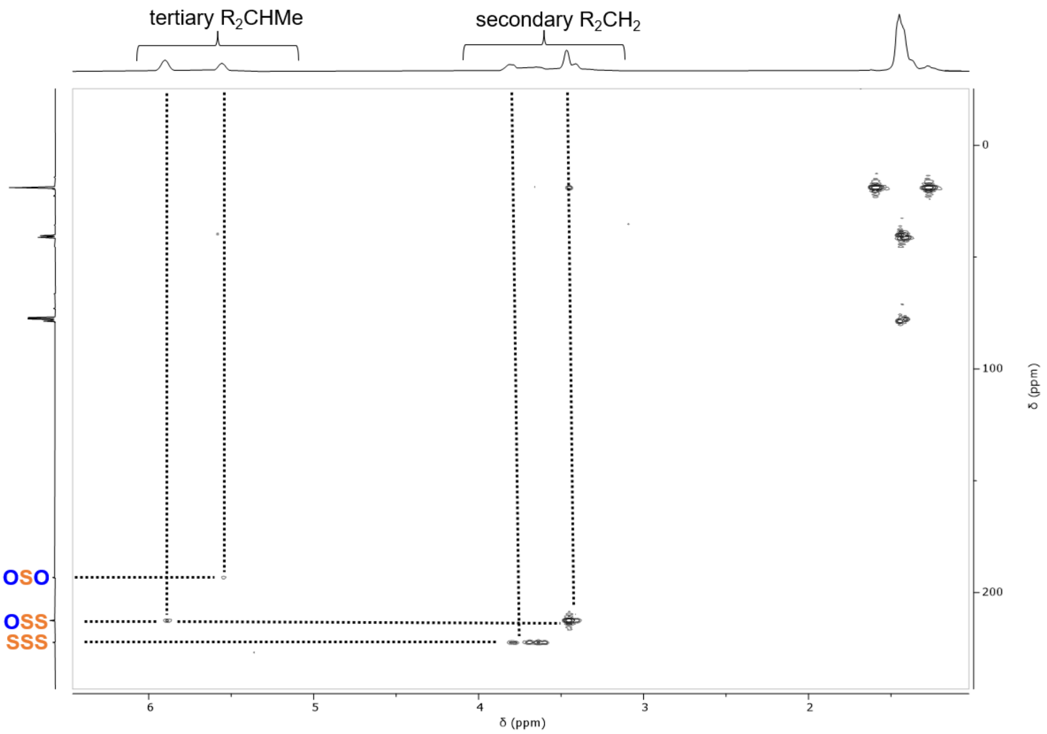

Supplementary Figure 82: ^{1}H-^{13}C HMBC NMR spectrum ($CDCl_3$, 25°C) of the isolated polymer corresponding to table 3, run #7.

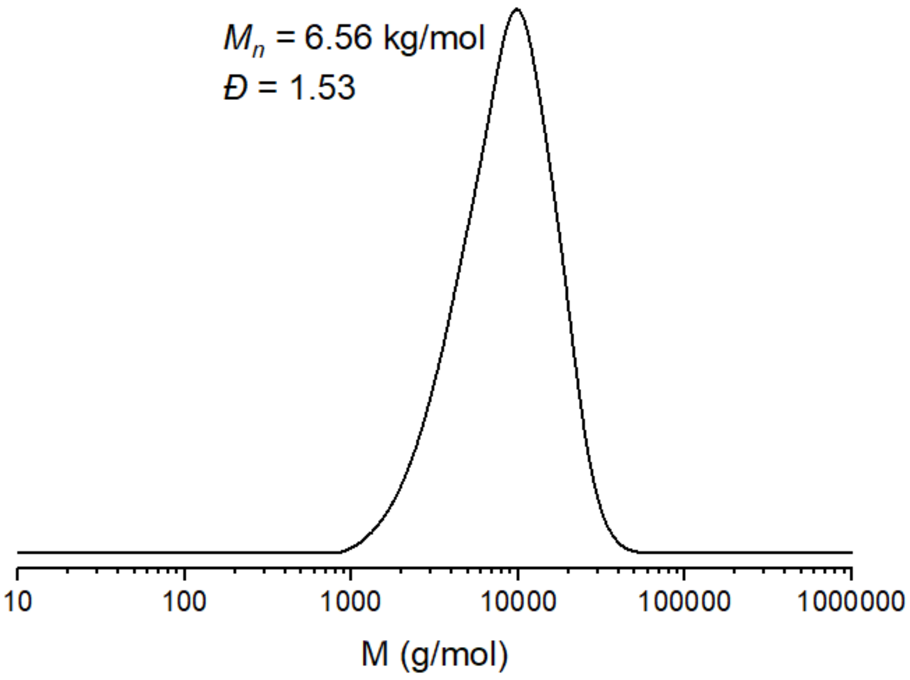

Supplementary Figure 83: GPC trace corresponding to table 3, run #7.

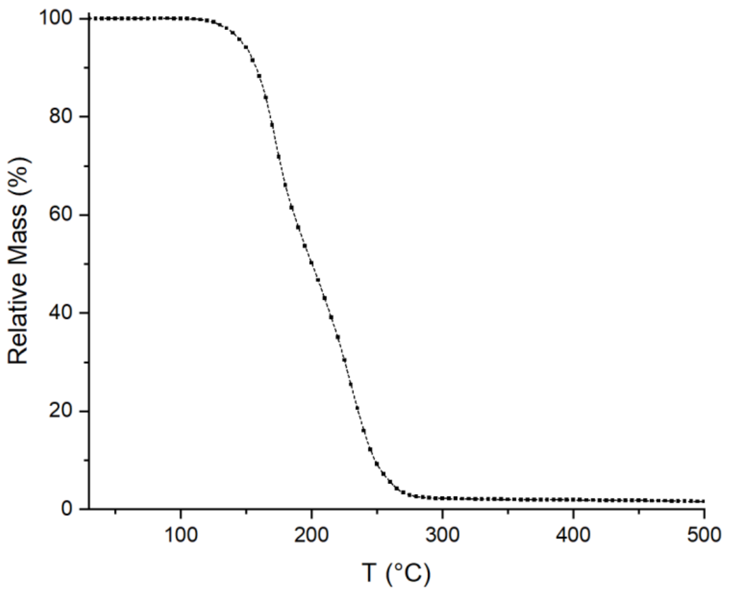

Supplementary Figure 84: TGA data corresponding to table 3, run #7. $T_{d,5\%}$ = 147.3 ºC.

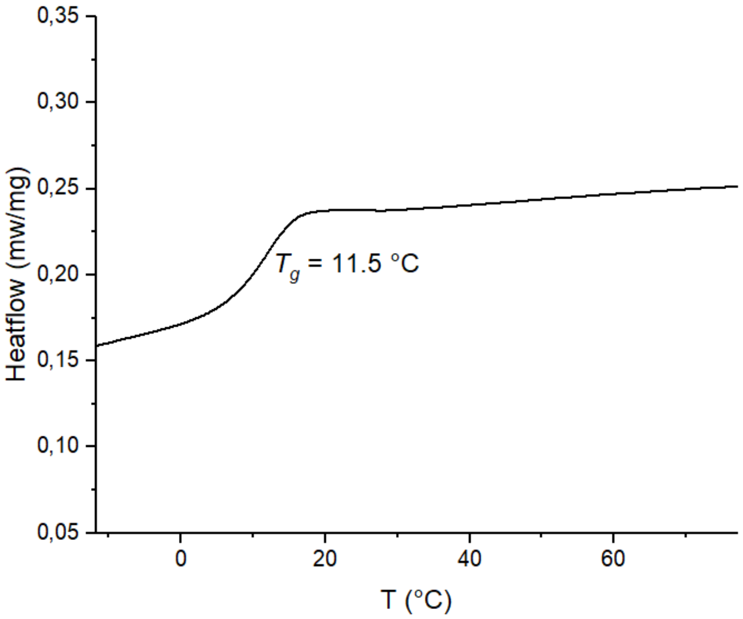

Supplementary Figure 85: DSC data from second heating cycle corresponding to table 3, run #7.

Supplementary Notes 8: De- and repolymerisation experiments

In analogy to reports by *Endo* and co-workers the polymer (1 eq.) was dissolved at 0.45 M concentration (treating the repeat unit as one entity) in THF containing 0.01 eq. KO^{t}Bu and let react at room temperature.[7] The reaction was monitored by ^{1}H NMR analysis of aliquots and stops after completion. Afterwards all solvent was removed in vacuum to obtained the cyclic dithiocarbonates as yellow (semi)solids.

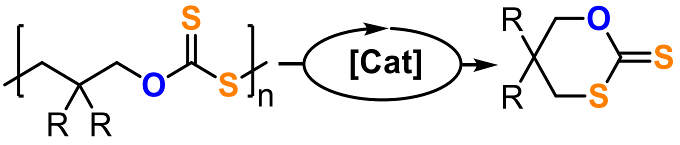

Supplementary Figure 86: Depolymerisation experiments

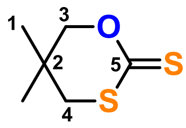

^{1}H NMR (600 MHz, CDCl$_3$, 25ºC) δ[ppm] = 4.16 (s, 2H, H3), 2.84 (s, 2H, H4), 1.17 (s, 6H, H1).^{13}C NMR (151 MHz, CDCl$_3$, 25ºC) δ[ppm] = 209.12 (C5), 81.26, 43.37, 28.11, 24.28. HREI-MS: calculated [M]$^+$ 162.0173. Found 162.0127.

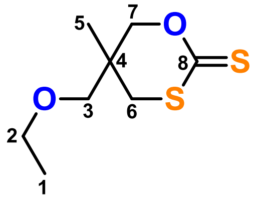

^{1}H NMR (600 MHz, CDCl$_3$, 25ºC) δ[ppm] = 4.41 (d, *J* = 11.3 Hz, 2H, H7), 4.22 (d, *J* = 11.3 Hz, 2H, H7), 3.49 (q, *J* = 7.0 Hz, 3H, 2H, H2), 3.43 – 3.27 (m, 2H, H3), 3.18 (d, *J* = 10.8 Hz, 1H, H6), 2.72 (d, *J* = 13.4 Hz, 1H, H6), 1.38 – 0.98 (m, 6H, H5+H1). ^{13}C NMR (151 MHz, CDCl$_3$, 25ºC) δ[ppm] = 209.52 (C8), 78.57, 73.96, 67.43, 38.99, 32.62, 20.07, 15.22. HREI-MS: calculated [M]$^+$ 218.0435. Found 218.0446.

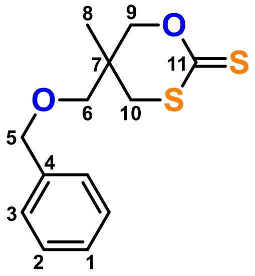

^{1}H NMR (600 MHz, CDCl$_3$, 25ºC) δ[ppm] = 7.53 – 7.27 (m, 5H, H1-H3), 4.53 (s, 2H, H5), 4.43 (dd, *J* = 11.4, 1.2 Hz, 1H, H9), 4.23 (dd, *J* = 11.4, 1.4 Hz, 1H, H9), 3.48 – 3.34 (m, 2H, H6), 3.19 (dd, *J* = 11.9, 1.1 Hz, 1H, H10), 2.74 (dd, *J* = 11.9, 1.5 Hz, 2H, H10), 1.20 (s, 3H, H8). ^{13}C NMR (151 MHz, CDCl$_3$, 25ºC) δ[ppm] = 209.14 (C11), 137.66, 128.65, 128.06, 127.72, 78.26, 73.66, 73.33, 38.82, 32.56, 19.96. HREI-MS: calculated [M]$^+$ 268.0592. Found 218.0651.

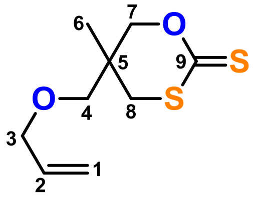

^{1}H NMR (401 MHz, CDCl$_3$, 25ºC) δ[ppm] = 5.86 (ddt, J = 17.2, 10.5, 5.6 Hz, 1H, H2), 5.39 – 4.97 (m, 2H, H1), 4.42 (dd, J = 11.4, 0.9 Hz, 1H, H7), 4.23 (dd, J = 11.4,1.3 Hz, 1H, H7), 3.99 (dt, J = 5.6, 1.4 Hz, 2H, H3), 3.43 – 3.30 (m, 2H, H4), 3.18 (dd, J = 11.9, 1.0 Hz, 1H, H8), 2.74 (d, J = 11.9 Hz, 1H, H8), 1.19 (s, 2H). ^{13}C NMR (151 MHz, CDCl$_3$, 25ºC) δ[ppm] = 209.36 (C9), 134.34, 117.99, 78.46, 73.48, 72.75, 38.99, 32.72, 20.16. HREI-MS: calculated [M]$^+$ 206.0435. Found 206.0477.

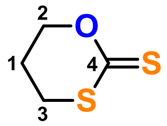

^{1}H NMR (401 MHz, CDCl$_3$, 25ºC) δ[ppm] = 5.06 – 4.23 (m, 2H, H2), 3.38 – 2.93 (m, 2H, H3), 2.47 – 2.13 (m, 2H, H1). ^{13}C NMR (151 MHz, CDCl$_3$, 25ºC) δ[ppm] = 209.12 (C4), 72.58, 30.96, 20.93.HREI-MS: calculated [M]$^+$ 133.9860. Found 133.9866.

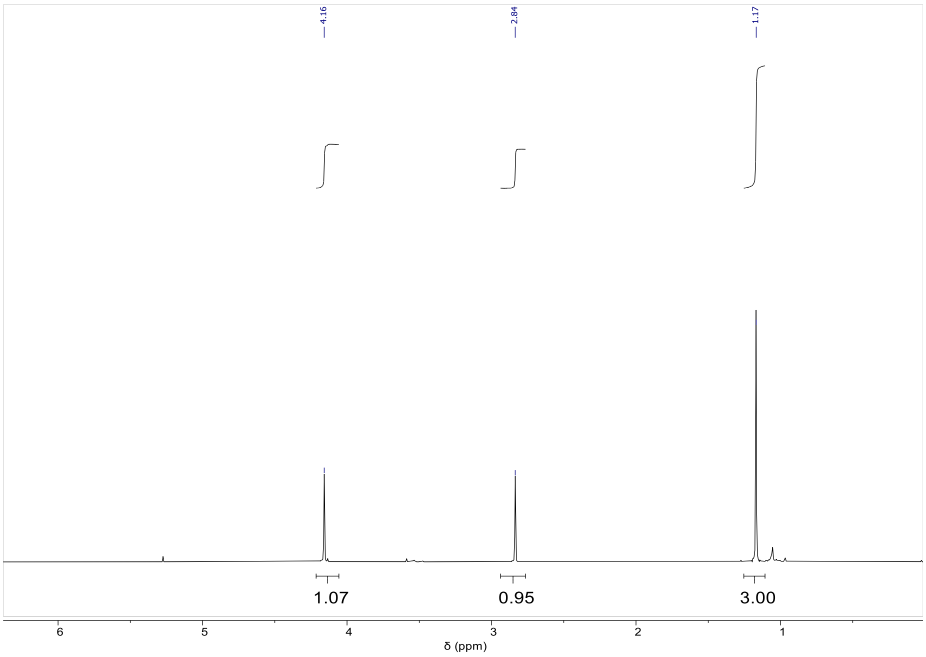

Supplementary Figure 87: ^{1}H NMR spectrum (600 MHz, $CDCl_3$, 25°C) of the depolymerisation product of the polymer corresponding to table 3, run #1.

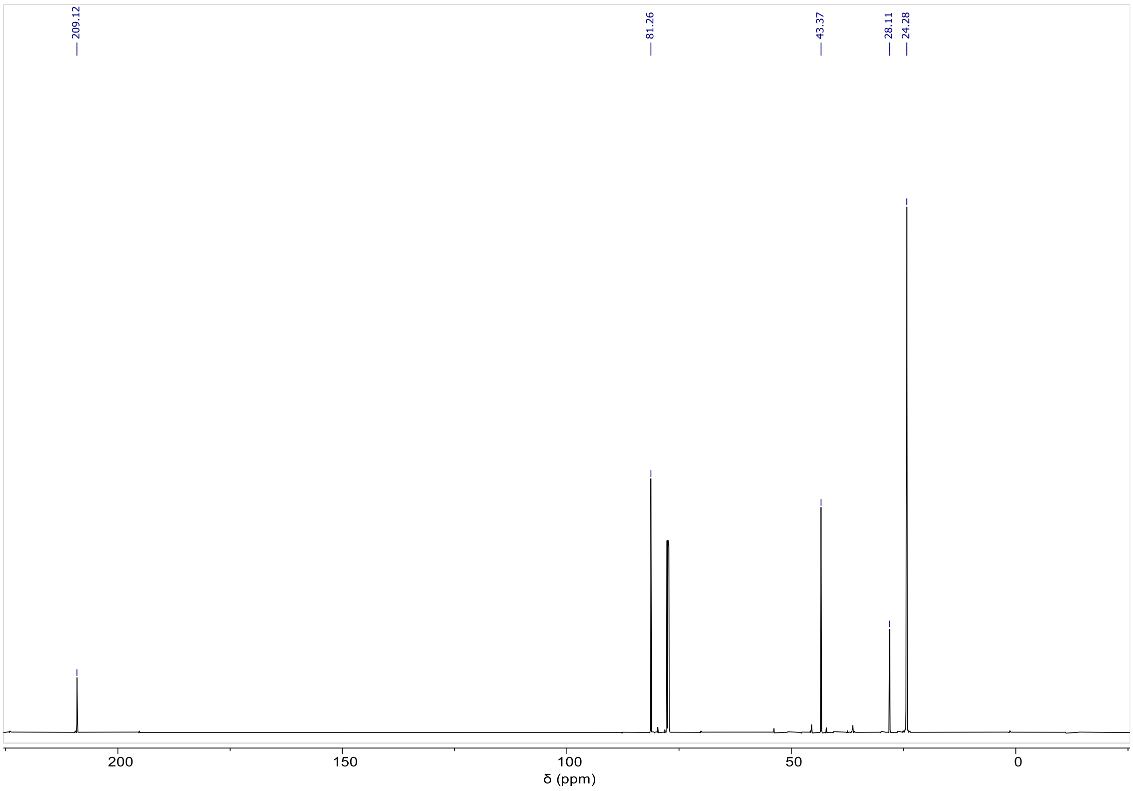

Supplementary Figure 88: $^{13}C\{^{1}H\}$ NMR spectrum (126 MHz, $CDCl_3$, 25°C) spectrum of the depolymerisation product of the polymer corresponding to table *3*, run #1.

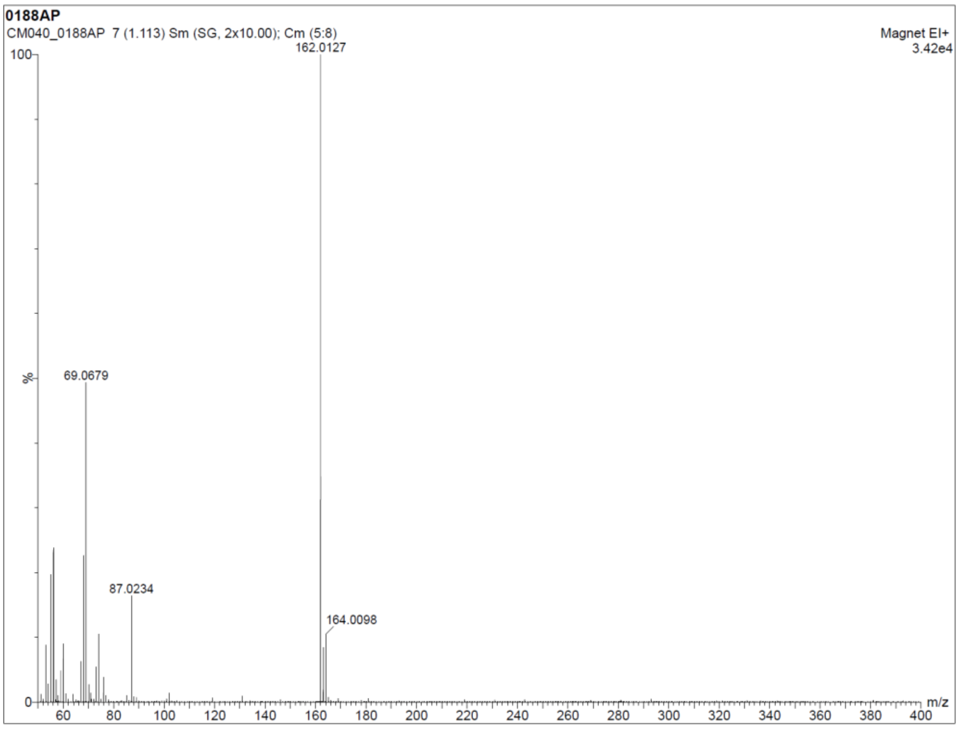

Supplementary Figure 89: EI-MS of the depolymerisation product of the polymer corresponding to table *3*, run #1.

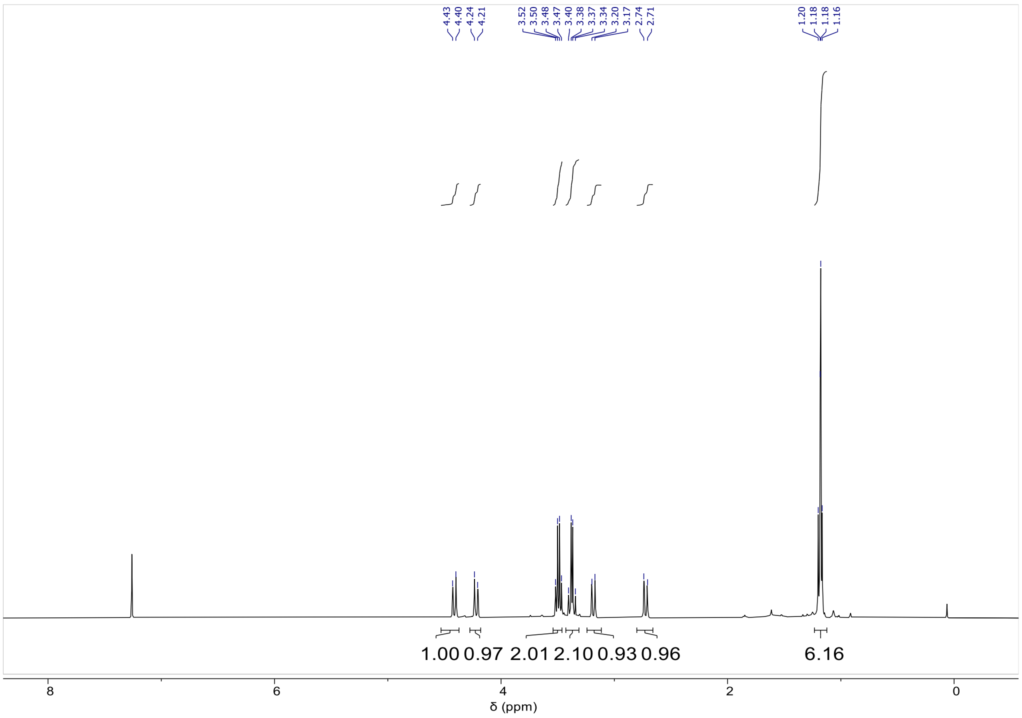

Supplementary Figure 90: ^{1}H NMR spectrum (600 MHz, $CDCl_3$, 25°C) of the depolymerisation product of the polymer corresponding to table 3, run #2.

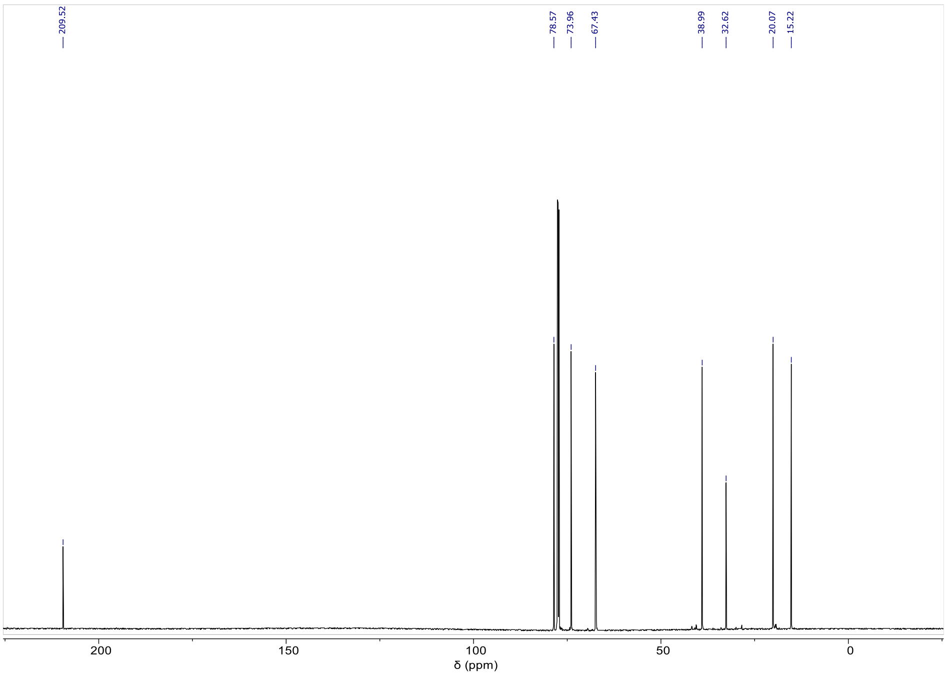

Supplementary Figure 91: $^{13}C\{^{1}H\}$ NMR spectrum (126 MHz, $CDCl_3$, 25°C) spectrum of the depolymerisation product of the polymer corresponding to table *3*, run #*2*.

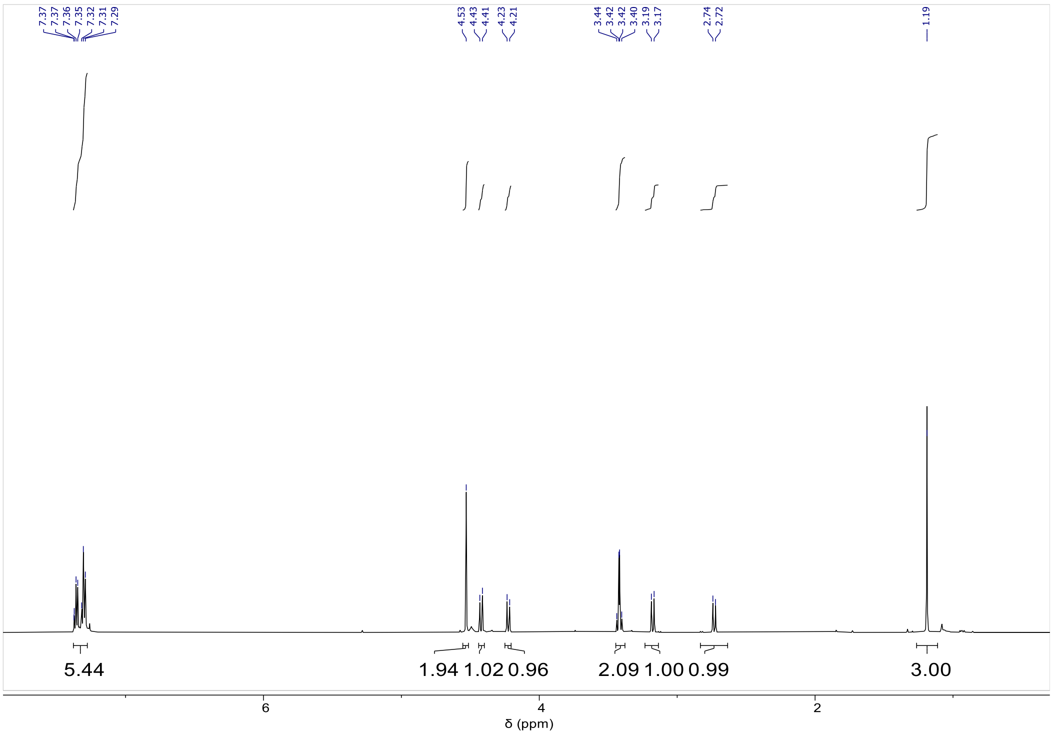

Supplementary Figure 92: ^{1}H NMR spectrum (600 MHz, $CDCl_3$, 25°C) of the depolymerisation product of the polymer corresponding to table 3, run #3.

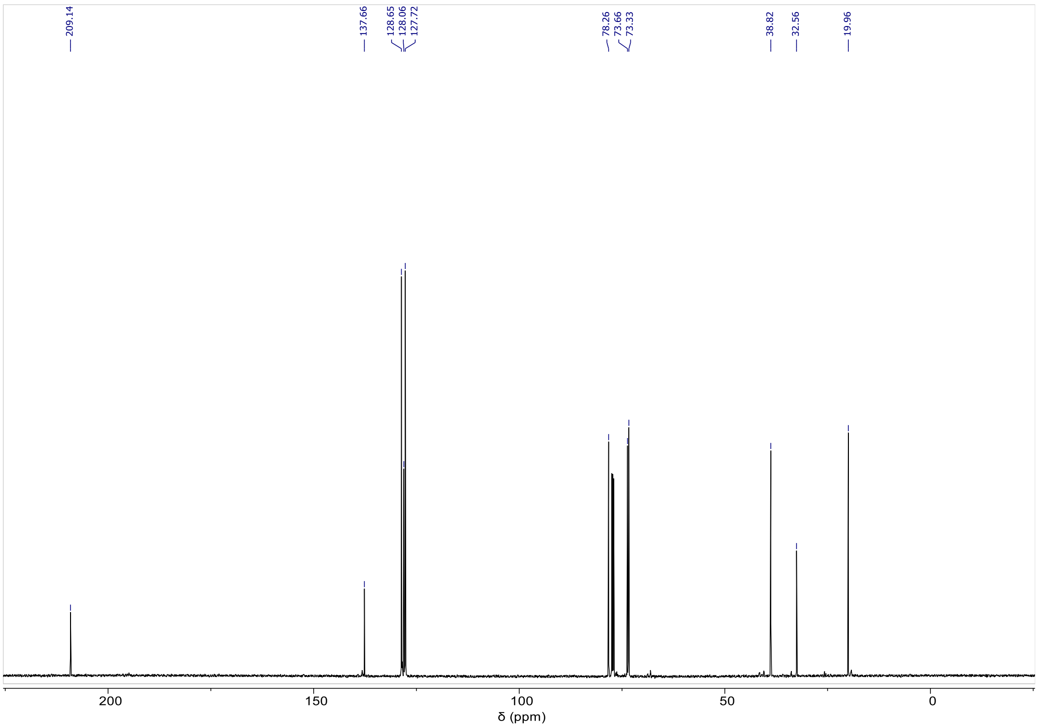

Supplementary Figure 93: $^{13}C\{^{1}H\}$ NMR spectrum (126 MHz, $CDCl_3$, 25°C) spectrum of the depolymerisation product of the polymer corresponding to table *3*, run #*3*.

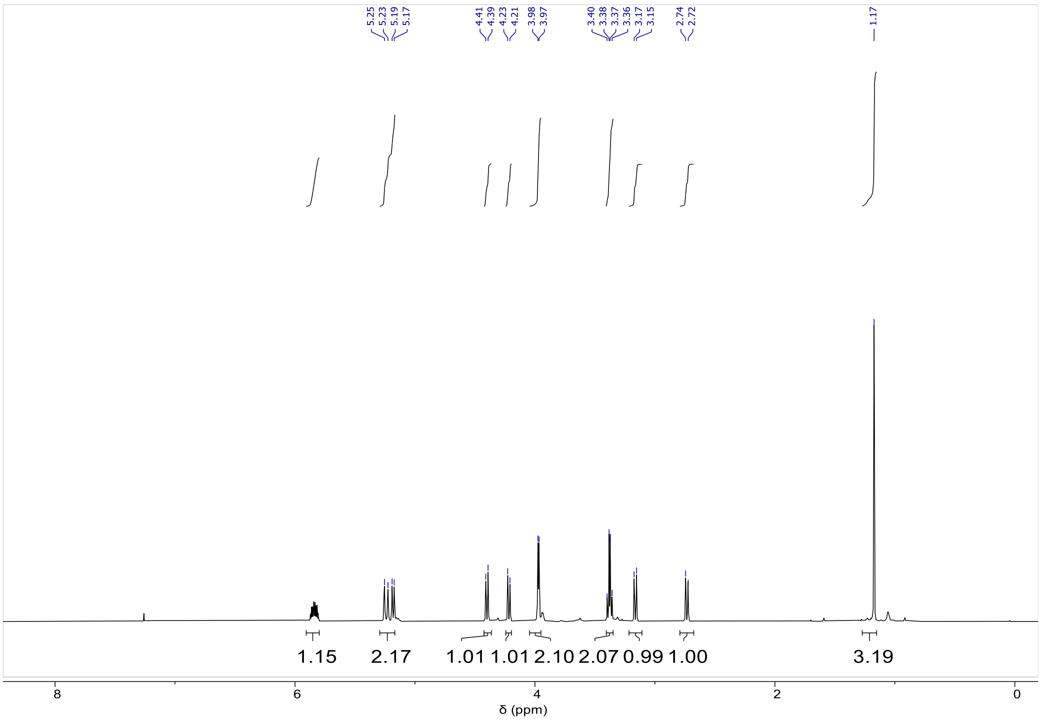

Supplementary Figure 94: ^{1}H NMR spectrum (600 MHz, $CDCl_3$, 25°C) of the depolymerisation product of the polymer corresponding to table 3, run #4.

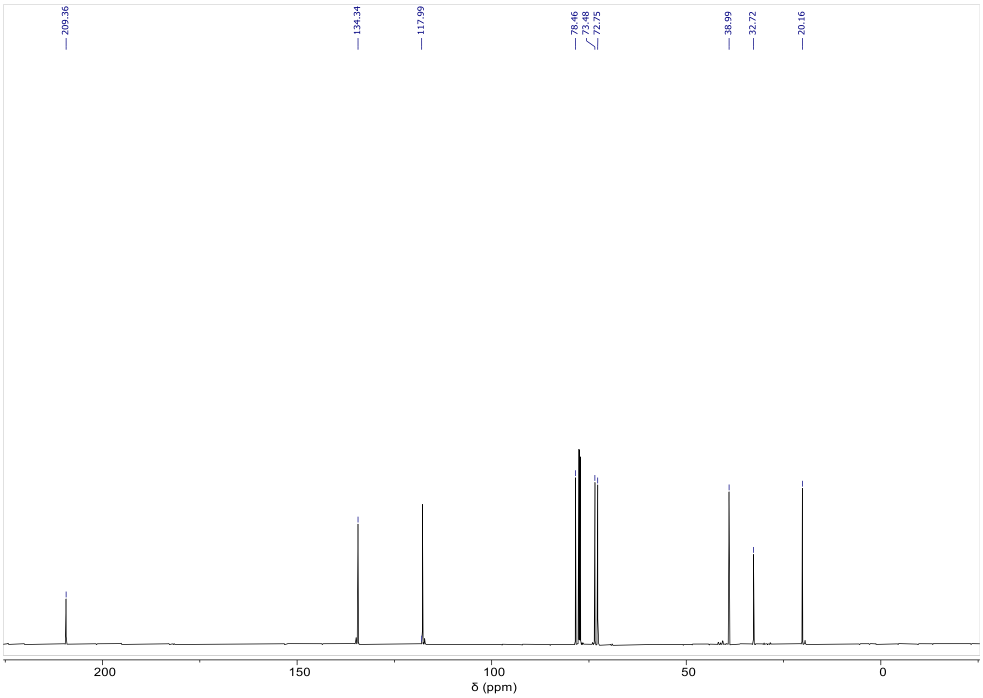

Supplementary Figure 95: $^{13}C\{^{1}H\}$ NMR spectrum (126 MHz, $CDCl_3$, 25°C) spectrum of the depolymerisation product of the polymer corresponding to table *3*, run #4.

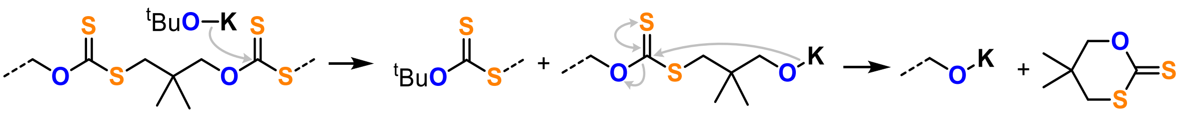

Supplementary Figure 96: Proposed depolymerisation mechanism.

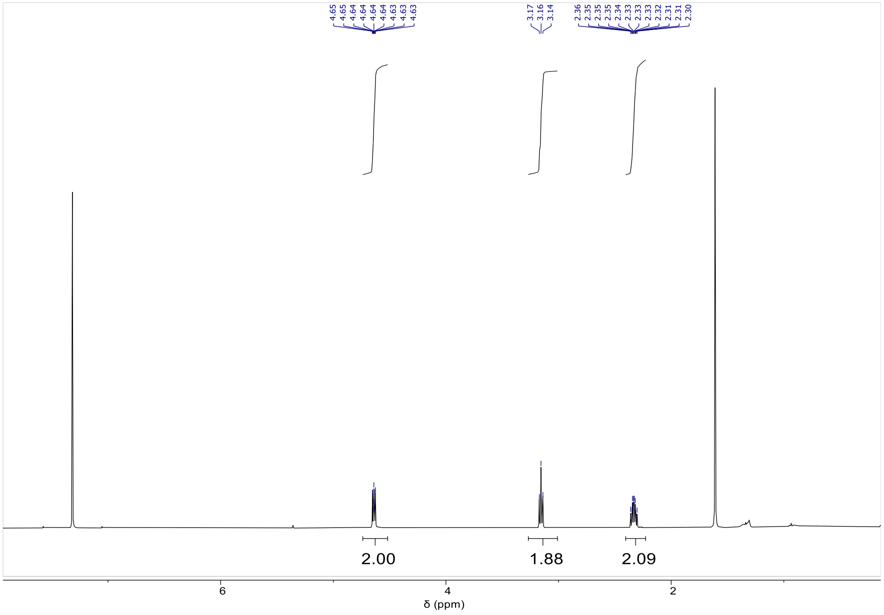

Supplementary Figure 97: ^{1}H NMR spectrum (600 MHz, $CDCl_3$, 25°C) of the depolymerisation product of the polymer corresponding to table 1, run #1.

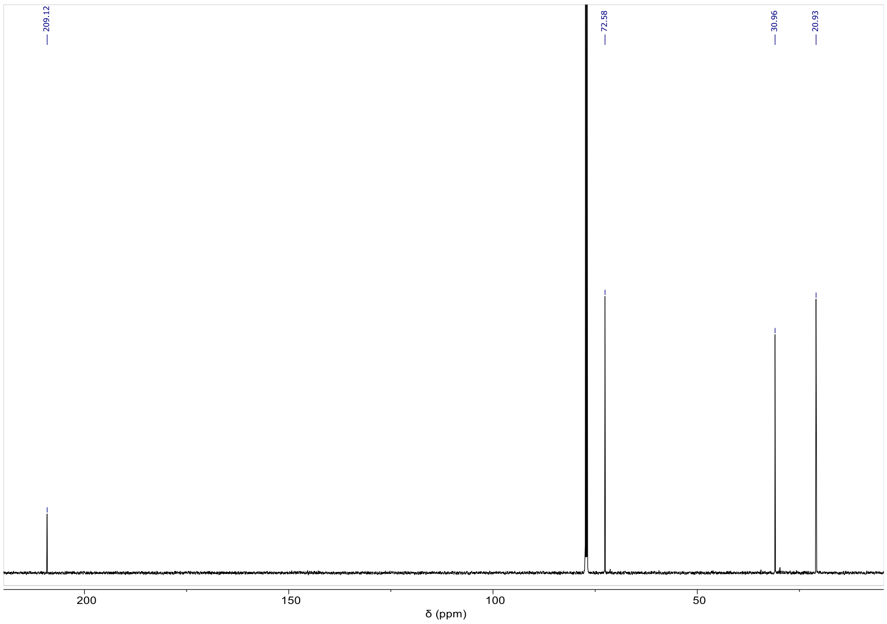

Supplementary Figure 98: $^{13}C\{^1H\}$ NMR spectrum (126 MHz, $CDCl_3$, 25°C) spectrum of the depolymerisation product of the polymer corresponding to table 1, run #1.

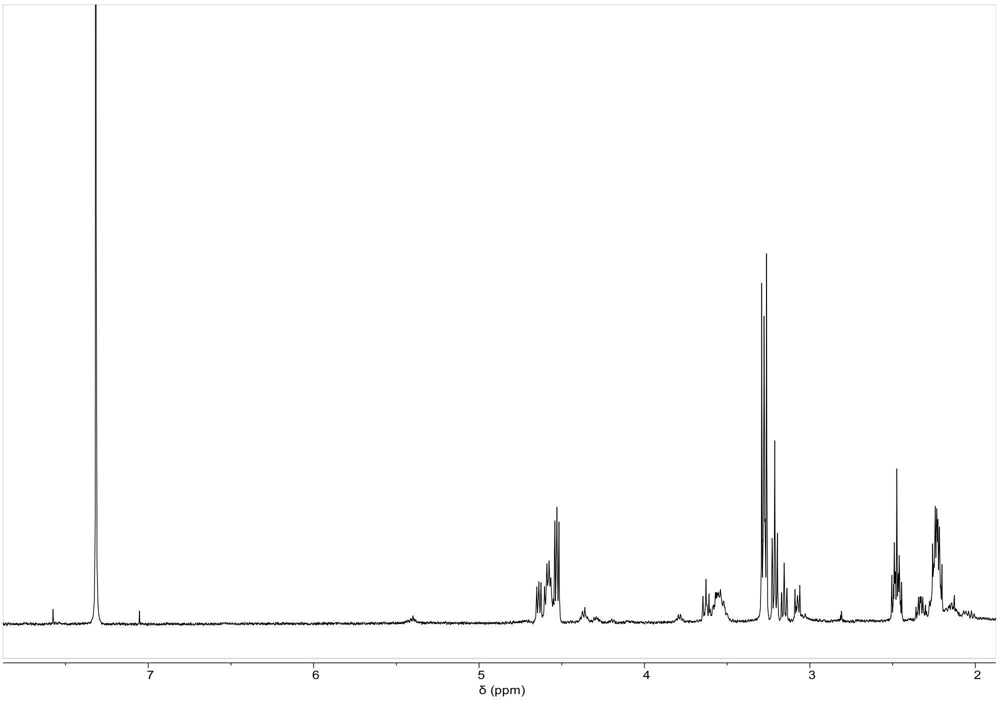

Supplementary Figure 99: ^1H NMR spectrum (600 MHz, $CDCl_3$, 25°C) of the depolymerisation products of the polymer corresponding to table 2, run #7.

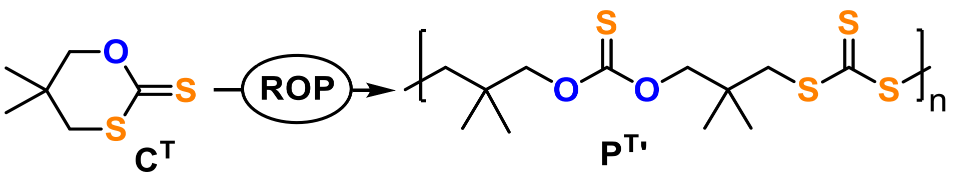

Supplementary Figure 100: Repolymerisation by ROP.

Repolymerisation procedure: **C**^{T} (660.0 mg, 25 eq.) was dissolved at 4 M concentration in dry DCM and benzylalcohol (4.2 µL, 1 eq.) and 1,5,7-triazabicyclo[4.4.0]dec-5-en (5.7 mg, 1 eq.) were added. The resulting mixture was stirred for 16h at room temperature and then added to 45 mL of MeOH acidified with a few drops of aqueous HCl resulting in the precipitation of the product. The product was redissolved in 1mL DCM and precipitated into pentane and dried in a vacuum oven set to 40ºC overnight to yield the polymer as a sticky yellow solid (300 mg, 45% yield).

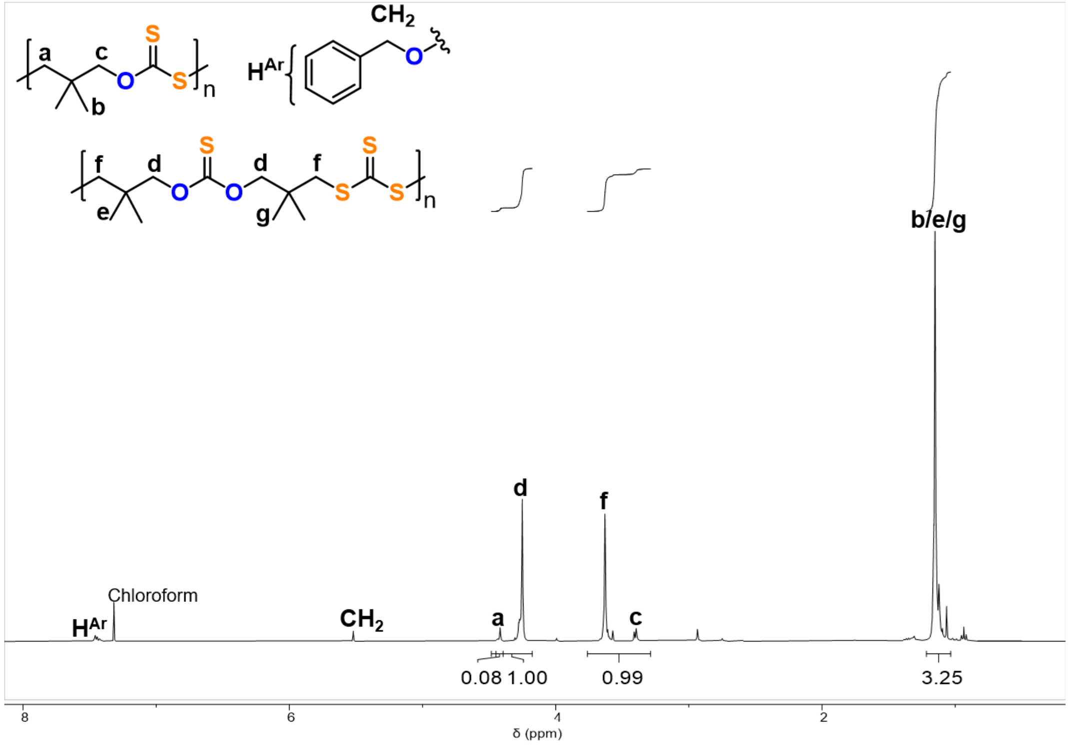

Supplementary Figure 101: ^{1}H NMR spectrum (400 MHz, $CDCl_3$, 25°C) of the polymer obtained from repolymerisation experiment. Assignment according to links observed in CS_2/OX^{Me} copolymer.

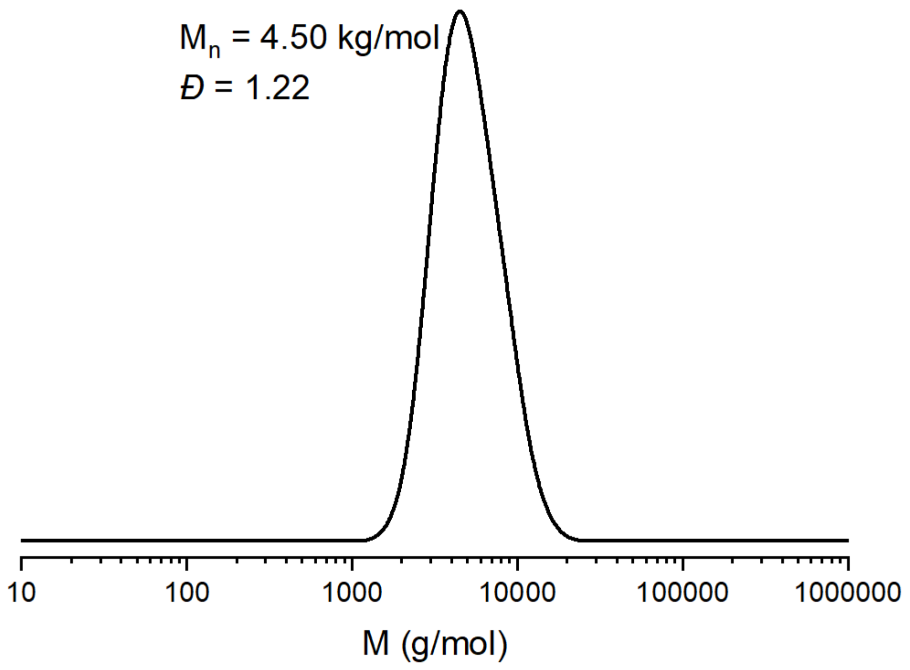

Supplementary Figure 102: GPC trace corresponding to repolymerisation experiment.

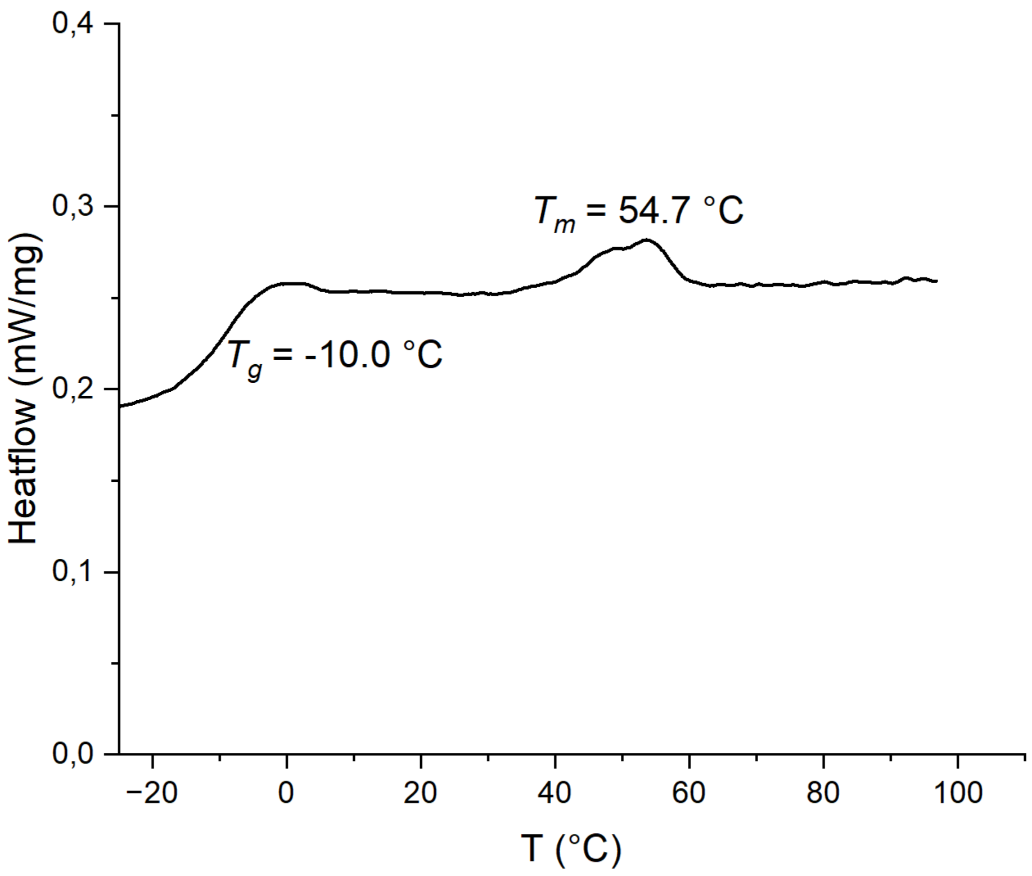

Supplementary Figure 103: DSC data from first heating cycle of copolymer corresponding to repolymerisation experiment.

Supplementary Notes 9: Mechanistic hypothesis explaining links and by-products

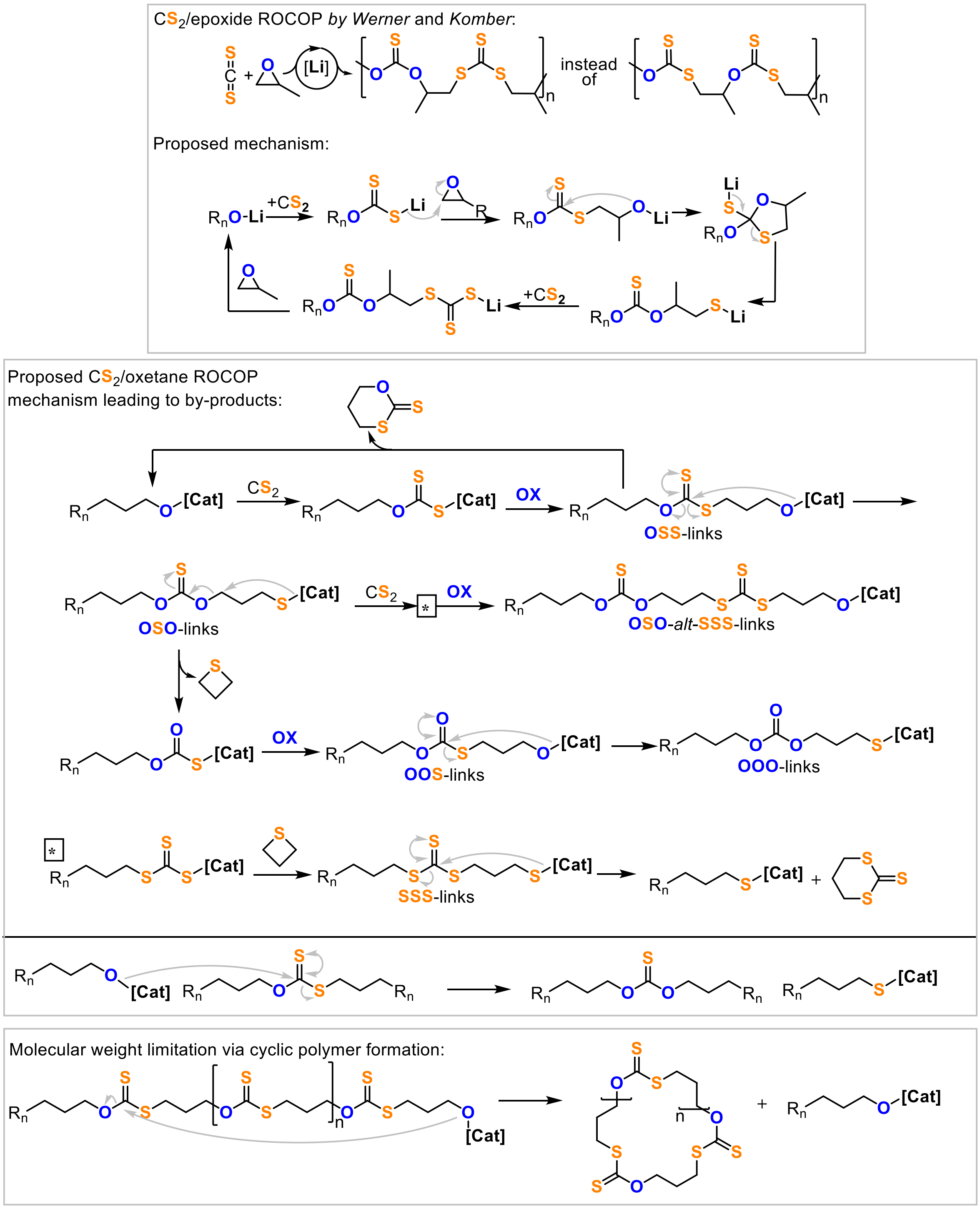

Supplementary Figure 104: (Top) O/S scrambling hypothesis by *Werner* and *Komber.* (Middle) Adapted hypothesis for CS_2/OX ROCOP explaining the formation of scrambled links and cyclic byproducts. (Bottom) Mechanistic suggestion for the formation of cyclic polymer.

Supplementary Notes 10: Density functional theory

The individual association energy per unit in reference to Figure 6 of the main manuscript are:

n	E = S	E = O
0	-33.32 kJ/mol	-31.09 kJ/mol
1	-34.85 kJ/mol	-21.45 kJ/mol
2	-36.45 kJ/mol	-23.21 kJ/mol
3	-37.39 kJ/mol	-23.58 kJ/mol

.

Supplementary References

[1] Y. Song, H. Jing, B. Li, D. Bai, *Chem. Eur. J.* **2011**, *17*, 8731–8738.
[2] C. J. Van Staveren, Johan. Van Eerden, F. C. J. M. Van Veggel, Sybolt. Harkema, D. N. Reinhoudt, *J. Am. Chem. Soc.* **1988**, *110*, 4994–5008.
[3] N. Yu. Kozitsyna, M. V. Martens, I. P. Stolyarov, A. E. Gekhman, M. N. Vargaftik, I. I. Moiseev, *Russ. Chem. Bul.l* **1999**, *48*, 1673–1681.
[4] J. Stephan, M. R. Stühler, S. M. Rupf, S. Neale, A. J. Plajer, ChemRxiv, DOI: 10.26434/chemrxiv-2023-wt5rf
[5] M. Luo, X.-H. Zhang, D. J. Darensbourg, *Macromolecules* **2015**, *48*, 5526–5532.
[6] A. J. Plajer, *ChemCatChem* **2022**, *14*, e2022008.
[7] J. Matsuo, K. Aoki, F. Sanda, T. Endo, *Macromolecules* **1998**, *31*, 4432–4438.
[8] R. Dovesi, A. Erba, R. Orlando, C. M. Zicovich-Wilson, B. Civalleri, L. Maschio, M. Rérat, S. Casassa, J. Baima, S. Salustro, B. Kirtman, *WIREs Comp. Mol. Sci.***2018**, *8*, e1360.
[9] S. Grimme, *J. Comp. Chem.* **2006**, *27*, 1787–1799.
[10] S. Grimme, J. Antony, S. Ehrlich, H. Krieg, *J. Chem. Phys.* **2010**, *132*, 154104.
[11] T. H. Dunning Jr., *J. Chem. Phys.* **1989**, *90*, 1007–1023.
[12] D. E. Woon, T. H. Dunning Jr., *J. Chem. Phys.* **1993**, *98*, 1358–1371.
[13] M. J. Frisch, G. W. Trucks, H. B. Schlegel, G. E. Scuseria, M. A. Robb, J. R. Cheeseman, G. Scalmani, V. Barone, G. A. Petersson, H. Nakatsuji, X. Li, M. Caricato, A. V. Marenich, J. Bloino, B. G. Janesko, R. Gomperts, B. Mennucci, H. P. Hratchian, J. V. Ortiz, A. F. Izmaylov, J. L. Sonnenberg, D. Williams-Young, F. Ding, F. Lipparini, F. Egidi, J. Goings, B. Peng, A. Petrone, T. Henderson, D. Ranasinghe, V. G. Zakrzewski, J. Gao, N. Rega, G. Zheng, W. Liang, M. Hada, M. Ehara, K. Toyota, R. Fukuda, J. Hasegawa, M. Ishida, T. Nakajima, Y. Honda, O. Kitao, H. Nakai, T. Vreven, K. Throssell, J. A. Montgomery, Jr., J. E. Peralta, F. Ogliaro, M. J. Bearpark, J. J. Heyd, E. N. Brothers, K. N. Kudin, V. N. Staroverov, T. A. Keith, R. Kobayashi, J. Normand, K. Raghavachari, A. P. Rendell, J. C. Burant, S. S. Iyengar, J. Tomasi, M. Cossi, J. M. Millam, M. Klene, C. Adamo, R. Cammi, J. W. Ochterski, R. L. Martin, K. Morokuma, O. Farkas, J. B. Foresman, and D. J. Fox, Gaussian 16, Gaussian, Inc., Wallingford CT, 2016.
[14] T. Lu, F. Chen, *J. Comp. Chem.* **2012**, *33*, 580–592.
